# The Determining Effective Testing in Emergency Departments and Care Coordination on Treatment Outcomes (DETECT) for Hepatitis C (Hep C) Screening Trial: rationale and design of a multi-center pragmatic randomized clinical trial of hepatitis C screening in emergency departments

**DOI:** 10.1186/s13063-022-06265-1

**Published:** 2022-04-25

**Authors:** Jason S. Haukoos, Sarah E. Rowan, James W. Galbraith, Richard E. Rothman, Yu-Hsiang Hsieh, Emily Hopkins, Rachel A. Houk, Matthew F. Toerper, Kevin F. Kamis, Jake R. Morgan, Benjamin P. Linas, Alia A. Al-Tayyib, Edward M. Gardner, Michael S. Lyons, Allison L. Sabel, Douglas A. E. White, David L. Wyles, for The DETECT Hep C Trials Investigators, Amy Adler, Musheng Alishahi, Gideon D. Avornu, Alexis Becerra, Erika Becerra-Ashby, Samantha Bot, Alexander J. Boyle, Annetta M. Bracey, Michael Breyer, Claudia Camacho, Alicia Cupelo, Gaby Dashler, Pamela Doyle, Amy Eicher, Heather Gardner, Carrie Anne de Gruiter, Stephanie Gravitz, Sophia Henry, David Higgins, Trevor Hill, Nyah Johnson, Alex Kile, Janet Liebl, Carolynn Lyle, Barbara Maliszewski, Kendall Maliszewski, Robert McGoey, Catherine McKenzie, Matthew S. Minturn, Deanna Myer, Kendra Neumann, Cole Ossian, Rebekah K. Peacock, Danielle Perez, Tannishtha Pramanick, Erin P. Ricketts, Benji Riggan, Sherry Riser, Genie Roosevelt, Mustapha Saheed, Bradley Shy, Scott Simpson, Gil Trest, Madison Unsworth, Laura Waltrous, Brooke Watson

**Affiliations:** 1 Department of Emergency Medicine, Denver Health Medical Center and University of Colorado School of Medicine, 777 Bannock Street, Mail Code 0108, Denver, CO 80204 USA; 2 Department of Epidemiology, Colorado School of Public Health, Aurora, CO USA; 3 Division of Infectious Diseases, Denver Health Medical Center and University of Colorado School of Medicine, Denver, CO USA; 4 Public Health Institute at Denver Health, Denver, CO USA; 5 Department of Emergency Medicine, University of Mississippi Medical Center, Jackson, MS USA; 6 Department of Emergency Medicine, Johns Hopkins University, Baltimore, MD USA; 7 Department of Informational Technology, Denver Health, Denver, CO USA; 8 Department of Health Law, Policy, and Management, Boston University School of Public Health, Boston, MA USA; 9 Center for Health Economics of Treatment Interventions for Substance Use Disorder, HCV, and HIV, Boston, MA USA; 10 Division of Infectious Diseases, Boston University School of Medicine, Boston, MA USA; 11 Department of Emergency Medicine, University of Cincinnati Medical Center, Cincinnati, OH USA; 12 Center for Addiction Research, University of Cincinnati, Cincinnati, OH USA; 13 Department of Patient Safety and Quality, Denver Health, Denver, CO USA; 14 Department of Biostatistics, Colorado School of Public Health, Aurora, CO USA; 15 Department of Emergency Medicine, Highland Hospital, Alameda Health System, Oakland, CA USA

**Keywords:** Hepatitis C, HCV, Screening, Testing, Clinical trial, Pragmatic trial, Randomized trial, Emergency department, Prevention, Comparative effectiveness, Methods, Implementation

## Abstract

**Background:**

Early identification of HCV is a critical health priority, especially now that treatment options are available to limit further transmission and provide cure before long-term sequelae develop. Emergency departments (EDs) are important clinical settings for HCV screening given that EDs serve many at-risk patients who do not access other forms of healthcare. In this article, we describe the rationale and design of The Determining Effective Testing in Emergency Departments and Care Coordination on Treatment Outcomes (DETECT) for Hepatitis C (Hep C) Screening Trial.

**Methods:**

The DETECT Hep C Screening Trial is a multi-center prospective pragmatic randomized two-arm parallel-group superiority trial to test the comparative effectiveness of nontargeted and targeted HCV screening in the ED with a primary hypothesis that nontargeted screening is superior to targeted screening when identifying newly diagnosed HCV. This trial will be performed in the EDs at Denver Health Medical Center (Denver, CO), Johns Hopkins Hospital (Baltimore, MD), and the University of Mississippi Medical Center (Jackson, MS), sites representing approximately 225,000 annual adult visits, and designed using the PRECIS-2 framework for pragmatic trials. When complete, we will have enrolled a minimum of 125,000 randomized patient visits and have performed 13,965 HCV tests. In Denver, the Screening Trial will serve as a conduit for a distinct randomized comparative effectiveness trial to evaluate linkage-to-HCV care strategies. All sites will further contribute to embedded observational studies to assess cost effectiveness, disparities, and social determinants of health in screening, linkage-to-care, and treatment for HCV.

**Discussion:**

When complete, The DETECT Hep C Screening Trial will represent the largest ED-based pragmatic clinical trial to date and all studies, in aggregate, will significantly inform how to best perform ED-based HCV screening.

**Trial registration:**

ClinicalTrials.gov ID: NCT04003454. Registered on 1 July 2019.

**Supplementary Information:**

The online version contains supplementary material available at 10.1186/s13063-022-06265-1.

## Introduction

**Fig. 1:**
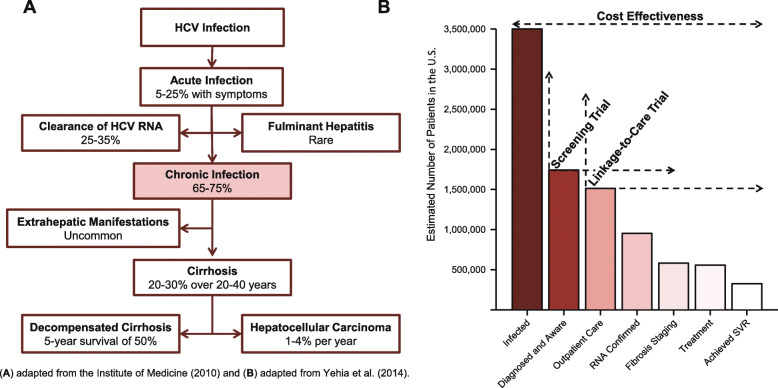
Natural progression of hepatitis C virus (HCV) infection (**A**) and the HCV care continuum (**B**)

Hepatitis C (HCV) is the most common blood-borne infection in the USA, and the burden of disease is substantial and increasing [1]. As of 2018, 2.5 million individuals were estimated to be living with HCV and > 50% remain undiagnosed [2, 3]. Well-tolerated, highly effective, direct-acting antivirals (DAAs) have revolutionized HCV treatment, dramatically reducing morbidity, mortality, and transmission, although significant work remains to improve the HCV care continuum (Fig. 1) [4–7].

Screening for HCV is the crucial initial step for intervening among patients with undiagnosed HCV [7, 8]. The Centers for Disease Control and Prevention (CDC) currently recommends HCV screening for all adults aged 18 years or older at least once in a lifetime and routine periodic screening for individuals with ongoing risk. Despite these recommendations, in many healthcare settings, screening for HCV is driven by risk [9, 10]. However, individuals commonly do not know they are at risk, clinicians may be too busy to perform risk assessments, or patients may be unwilling or unable to provide risk information [11]. Thus, support for the performance of non-risk-based (nontargeted) screening has grown, similar to what was seen with HIV screening [12–14].

Emergency departments (EDs) are important clinical settings for infectious diseases screening, including HCV, given that they serve as our society’s medical safety-net by, in part, filling the gap for those who do not have consistent access to healthcare [15–18]. Over 135 million ED visits occur annually and EDs serve substantial numbers of at-risk patients, providing ample opportunity to identify patients with undiagnosed HCV [19]. Nontargeted versus targeted HCV screening and different linkage-to-care approaches have not been studied in head-to-head fashions in EDs, thus the comparative effectiveness and yield of these strategies remains unknown.

The objectives of The Determining Effective Testing in Emergency Departments and Care Coordination on Treatment Outcomes (DETECT) for Hepatitis C (Hep C) studies are to (1) compare the effectiveness of two forms of HCV screening—nontargeted and targeted HCV screening (Screening Trial); (2) compare the effectiveness of two forms of referral to treatment for ED patients identified with HCV—linkage navigation plus clinician referral versus clinician referral only (Linkage-to-Care Trial); (3) measure and compare programmatic costs and project long-term clinical outcomes, costs, and cost effectiveness of ED-based HCV screening and linkage-to-care (Cost Evaluation); and (4) examine the effects of disparities and social determinants of health on linkage-to-care, treatment initiation, and treatment completion among patients identified with HCV in the ED (Disparities and Social Determinants of Health Evaluation). This article describes the rationale and design of the Screening Trial and is reported in accordance with the SPIRIT Statement for clinical trials [20, 21]. The primary hypothesis of the Screening Trial is that nontargeted HCV screening is significantly associated with increased identification of new HCV diagnoses when compared to targeted HCV screening. The full Protocol (Version 3.3, Date February 17, 2022) is provided in the Supplemental Appendix.

## Methods

The DETECT Hep C Screening Trial was designed using the PRECIS-2 framework for pragmatic clinical trials (Figure S1), has been registered in ClinicalTrials.gov, and will be reported in accordance with CONSORT guidelines [22, 23].

### Trial design

**Fig. 2:**
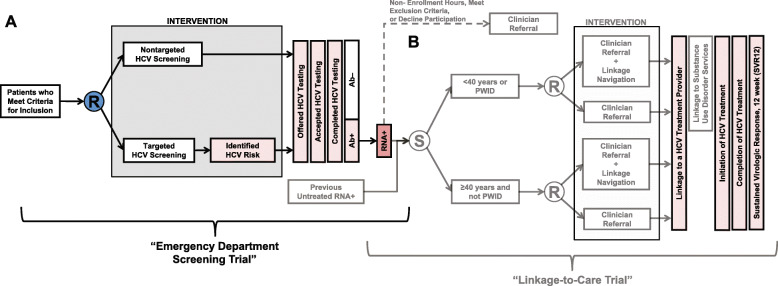
Study schematic for The DETECT Hep C Trials, including the Emergency Department (ED) Screening Trial (**A**) and the ED Linkage-to-Care Trial (**B**). Abbreviations: HCV, hepatitis C; PWID, person who injects drugs

For this trial, we will perform a multi-center prospective pragmatic randomized two-arm parallel-group superiority trial, comparing two HCV screening strategies in the ED (Fig. 2). Allocation will be balanced using a 1:1 patient visit-level random allocation scheme built into the electronic health systems (EHSs) for each ED.

### Study setting

This trial will be performed in the EDs at Denver Health Medical Center (Denver, CO), Johns Hopkins Hospital (Baltimore, MD), and the University of Mississippi Medical Center (Jackson, MS) with a cumulative total of approximately 225,000 annual adult visits (Table 1). These sites were selected because of the heterogeneity of populations served. Each is geographically distinct and serves patient populations that are racially and ethnically diverse, underinsured, and have ongoing local HCV epidemics. Additionally, each ED has investigators that are experts in infectious diseases screening in EDs, which improves the likelihood of successful completion of this trial. Denver Health will serve as the coordinating site for this project.

**Table 1:** Study site characteristics for The DETECT Hep C Emergency Department Screening Trial

Site	Setting	Hospital type	Annual ED census (visits)	Hispanic or non-White race^a^ (%)	Uninsured patients^b^ (%)	Birth cohort^c^ (%)	PWID (%)
Denver Health MC	Urban	L1/C/SN/T	96,000	53%	17%	30%	7%
Johns Hopkins Hospital	Urban	L1/U/T	69,000	77%	15%	31%	6%
University of Mississippi MC	Urban	L1/U/T	65,000	58%	28%	32%	Unknown

*Abbreviations: MC* Medical center, *ED* Emergency department, *L1* Level 1 trauma center, *C* County, *SN* Safety-net, *U* University, *T* Teaching/academic, *PWID* Person who injects drugs

^a^Asian, Black, Hispanic/Latin, American or Alaskan Native, Native Hawaiian, or Non-Hawaiian Pacific Islander

^b^Defined as uninsured or sponsored by a state healthcare discount program. Does not include Medicaid or Medicare

^c^Defined as birth years from 1945 to 1965

### Eligibility criteria

Consecutive ED patients ≥18 years of age will be eligible for inclusion if they are considered clinically stable by screening nurses or physicians and capable of providing consent for medical care. Due to the integrated, pragmatic nature of the study, patients will be enrolled 24 h per day, 7 days per week. Patients will be excluded if they (1) are <18 years of age; (2) are unable to consent for care (e.g., altered mentation, critical illness or injury); (3) have already been tested for HCV as part of the trial; (4) are identified as already living with HCV, either by self-report or through identification of a prior positive HCV result in the electronic medical record; or (5) have an anticipated ED length of stay <60 min.

### Interventions

This trial consists of a comparative evaluation of two HCV screening strategies, nontargeted and targeted screening. Patients who are allocated to nontargeted screening will be notified by the nurse that voluntary free rapid HCV testing will be performed during the ED visit unless declined (i.e., opt-out consent). Patients who are allocated to targeted screening will be presented with a brief risk assessment (Table 2). Those who have an affirmative response to any question will be considered at increased risk for HCV and notified by the nurse that voluntary free rapid HCV testing will be performed during the ED visit unless declined (Figure S3). Patients in the targeted arm who deny all risk will be considered low risk and HCV testing will not be offered.

**Table 2:** Targeted risk assessment for HCV. Any affirmative response is considered at risk

1. Patient’s birth year between 1945 and 1965?	
2. Have you ever injected or snorted drugs?	
3. Do you have a tattoo or body piercing that you received in an unregulated setting?	
4. Have you ever had a blood transfusion or received an organ before July 1992?	

Adapted from the CDC, USPSTF, and AASLD-IDSA guidelines for HCV screening

Our brief risk assessment includes questions adapted from recommendations by the CDC, US Preventive Services Task Force (USPSTF), American Association for the Study of Liver Diseases (AASLD), and Infectious Diseases Society of America (IDSA). The questions ask if the patient was (1) born between 1945 and 1965, (2) ever injected or snorted drugs, (3) ever received a tattoo or piercing in an unregulated setting, or (4) received a blood transfusion or organ transplant before 1992 [9–11, 24]. Risk assessment will be incorporated into electronic medical screening and patient tracking systems in each ED. As such, nurses will use this tool while electronically entering responses to each of the risk questions during screening. Nurses will apply the set of risk questions to all patients who meet criteria for inclusion and are allocated to the targeted screening arm.

All HCV screening will be voluntary with consent for testing obtained in a verbal manner as part of clinical care and separate from consent for general emergency care. All processes will be fully integrated into usual emergency care with HCV antibody (Ab) test results returning during the ED visit. Central laboratory-based HCV Ab assays will be used at all institutions (Denver Health Medical Center: OraQuick Rapid HCV Antibody Test, OraSure Technologies, Bethlehem, PA; Johns Hopkins Hospital: Roche Elecsys® Anti-HCV II, Roche Diagnostics, Indianapolis, IN, and OraQuick Rapid HCV Antibody Test, OraSure Technologies, Bethlehem, PA (point-of-care samples); University of Mississippi Medical Center: ARCHITECT Anti-HCV assay, Abbott Laboratories, Abbott Park, IL), with subsequent HCV ribonucleic acid (RNA) assays for those who test Ab+.

### Allocation

#### Sequence generation, allocation concealment, and implementation

Participants who meet criteria for inclusion will be randomly assigned at the ED visit level in a 1:1 ratio to each study arm using simple randomization incorporated into the EHS (Epic, Epic Systems Corporation, Verona, WI) at each institution. Sequence generation will occur real-time in the background of the EHS using a computer-generated random number algorithm developed and validated at each site. Integration of randomization into the EHS will allow for real-time concealed allocation. As such, patients will be offered HCV testing based on the result of the screening arm to which they are assigned during triage of the ED visit. In the case of the targeted arm, the results of the risk assessment evaluation performed by the triage nurse will dictate HCV testing. All randomization and enrollment procedures will be completely integrated into EHS workflow at each site.

### Blinding

Nurses who perform screening and all other ED staff (e.g., physicians, technicians) will understand the conceptual goals of the trial but will be blinded to study hypotheses, and patients will be completely blinded to the purpose of the study.

### Recruitment

Given the pragmatic nature of the trial and the full integration of all study procedures, recruitment will occur 24 h per day, 7 days a week as part of actual ED care. As such, all patients who meet eligibility criteria will be included and randomized.

### Participant timeline

Primary research activities, including randomization, screening, testing, and referral to care for those who test positive for HCV antibodies (Abs), will occur during the patient’s ED visit. Longitudinal outcomes will be collected using structured retrospective chart review methods for those who test positive for HCV in the ED for a minimum of 6 months but up to 18 months from the time of diagnosis. There is no compensation for trial participation and all post-trial care will be usual care for patients.

### Outcomes

The *primary outcome* for this trial will be confirmed cases of newly diagnosed HCV, defined as patients who test positive for HCV Ab and HCV RNA. *Secondary outcomes* will include *all* patients identified with HCV in anticipation of testing those with previously diagnosed HCV (i.e., those who do not identify as having been previously diagnosed and who are re-diagnosed during this trial), as well as HCV test acceptance and completion, and progression through the HCV care continuum (i.e., receipt of RNA results, evaluation by a HCV treatment expert, treatment with DAAs, and sustained virologic response at 12 weeks after treatment completion [SVR12]).

### Sample size

#### Screening Trial—original sample size estimation

**Fig. 3:**
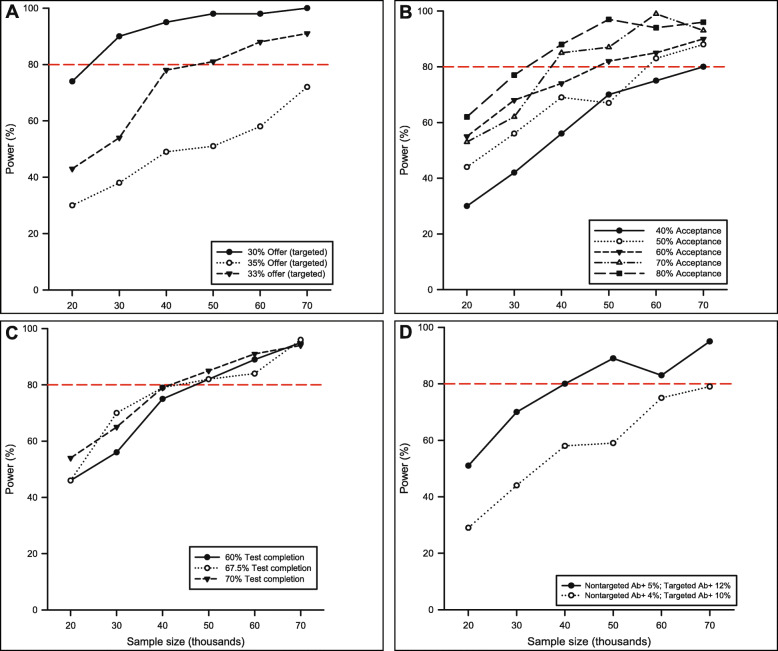
Original power simulations of test offer (**A**), test acceptance (**B**), completion (**C**), and hepatitis C (HCV) antibody positive prevalence (**D**). Each point from each panel represents 1000 Monte Carlo simulated trials of total randomized patient visits with all other assumptions held constant

All sample size estimates for this trial were performed using Monte Carlo simulation in SAS Enterprise Guide Version 7.1 (SAS Institute, Inc., Cary, NC). Informed by medical literature, past experiences with HIV screening in EDs, and pilot data, we made the following assumptions: (a) nontargeted screening, 100% test offer, 60% test acceptance, 70% test completion, 5% Ab+ prevalence, and 65% RNA+ prevalence among those who test HCV Ab+ and (b) targeted screening, 33% test offer, 60% test acceptance, 70% test completion, 10% Ab+ prevalence, and 65% RNA+ prevalence among those who test HCV Ab+. The original sample size estimate was performed using series of 1000 simulated trials and a hypothesized effect of a 25% increase in the number of new HCV diagnoses (i.e., risk ratio [RR] = 1.25), resulting in a minimum of 50,000 visits across all sites to achieve a power >80% (*α* = 0.05). This was estimated to result in 13,965 completed HCV tests and 611 confirmed newly diagnosed patients (Fig. 3).

Accounting for the multi-site nature of the trial, and using preliminary data from a similar trial of HIV screening in EDs [25], we estimate the intra-cluster correlation (ICC) to range from 0.005 to 0.01, resulting in a design effect of 1.01–1.03, or a required 1–3% inflation of the sample size. Given the small number of clusters (3 sites) and the relatively large number of patients enrolled at each site, we anticipate the effect of clustering to be negligible; as such, we did not specifically modify the sample size for clustering but will account for it in the primary analysis. Finally, enrollment is planned to perform equal numbers of HCV tests across sites, resulting in a minimum of 16,667 randomized patient visits per site.

#### Screening Trial—blinded sample size re-estimation

In early April 2021, this trial crossed the 50% enrollment target and the final site, the University of Mississippi Medical Center, initiated enrollment. While performing general surveillance and trial monitoring, several assumptions used for the original sample size estimation were identified as being different from what was being observed during actual trial performance (Table 3). As such, a blinded sample size re-estimation (SSR) was undertaken using Monte Carlo simulation in SAS [26].

**Table 3:** Original assumptions used to estimate sample size and weighted and inverse probability weighted estimates from observed trial performance through April 27, 2021, and 58.6% of target enrollment

	**Nontargeted screening**			**Targeted screening**		
	**Original assumptions**	**Weighted estimates**	**IPW estimates**	**Original assumptions**	**Weighted estimates**	**IPW estimates**
	**%**	**%**	**%**	**%**	**%**	**%**
**Test offer**	100	93.8	82.6	33	36.1	33.0
**Test accept**	60	26.4	33.0	60	34.0	47.0
**Test complete**	70	62.9	56.9	70	69.3	65.2
		**HCV testing**				
		**Original assumptions**	**Aggregate weighted estimates**	**Aggregate IPW estimates**	**Original assumptions**	
		**%**	**%**	**%**	**%**	
**HCV Ab+**		5	5.9	4.7	10	
**HCV RNA+**^a^		65	42.4	31.1	65	

*Abbreviations: HCV* Hepatitis C, *IPW* Inverse probability weighted

^a^Proportion of those who test HCV Ab+

As of April 27, 2021, the Screening Trial was at 58.6% enrolled and using these updated enrollment data, we project randomizing 129,663 visits across the three sites to complete 13,965 HCV antibody tests, as originally estimated. This increase in number of visits randomized reflects a 2.6-fold increase over the number originally estimated to be randomized (i.e., 50,000). Moreover, test acceptance (i.e., actual: 26% for nontargeted and 34% for targeted vs original: 60%) and RNA+ prevalence were significantly lower than originally estimated (i.e., actual: 42% vs original: 65%). Thus, we performed a series of additional simulations using methods described below to estimate the power to test the primary study hypothesis (Tables S1 and S2). Using both weighted and inverse probability weighted estimates to account for individual site contributions to total tests offered, tests accepted, and tests completed by study arm, while also using a two-way data table (Table S3) to model potential combinations of Ab+ prevalences by study arm using known aggregate Ab+ prevalence (and assuming RNA+ prevalence would not differ by study arm), we performed a series of additional simulations to estimate the power to test the primary study hypothesis.

**Fig. 4:**
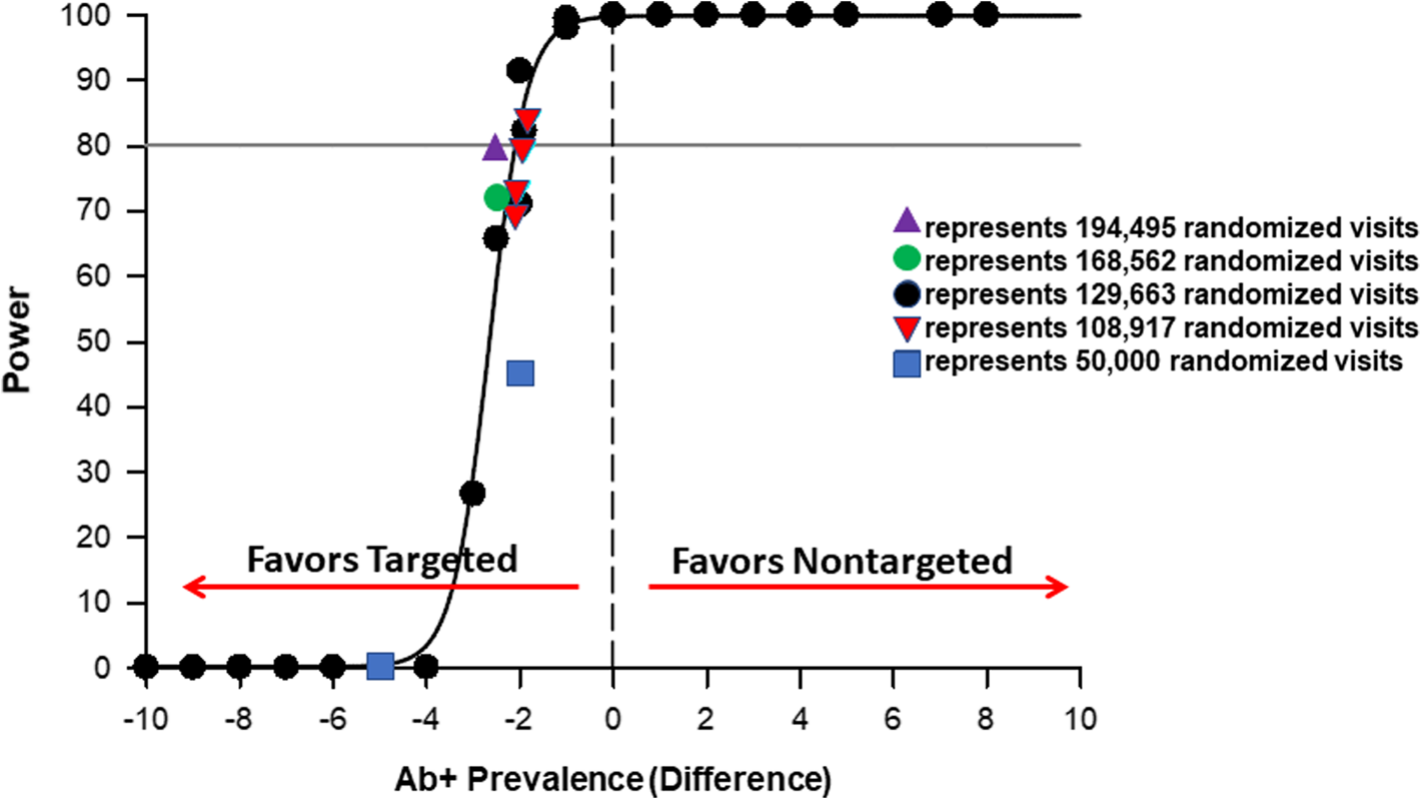
Sample size re-estimations using 1000 Monte Carlo simulations using weighted enrollment estimates and aggregate antibody positive (Ab+) of 5.9% to estimate power by Ab+ difference between nontargeted and targeted hepatitis C (HCV) screening

**Fig. 5:**
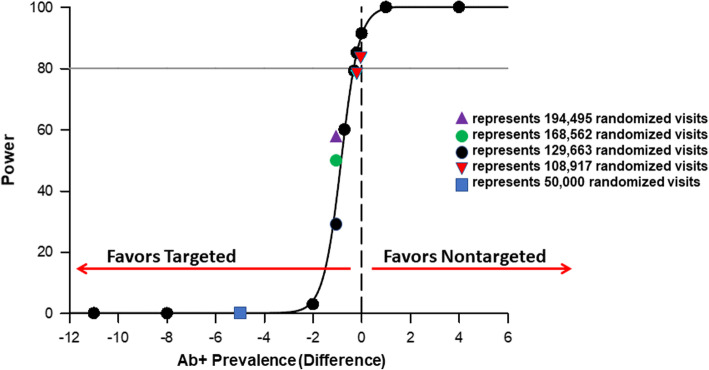
Sample size re-estimations using 1000 Monte Carlo simulations using inverse probability weighted enrollment estimates and aggregate antibody positive (Ab+) of 4.7% to estimate power by Ab+ difference between nontargeted and targeted hepatitis C (HCV) screening

Simulations using original assumptions and the originally planned 50,000 randomized visits, which the trial has already exceeded, resulted in significantly fewer total tests performed (*n* = 5,969) and power of only 0.3% (weighted and inverse probability weighted). Using the projected 129,663 randomized visits, estimates from current trial enrollment, and our original hypothesized effect estimate (i.e., RR = 1.25), simulations resulted in trials with totals of 15,452–15,623 (weighted) or 16,602–16,616 (inverse probability weighted) HCV Ab tests performed (exceeding our original estimation), while achieving powers of 65.7% (weighted) and 42.9% (inverse probability weighted). Fixing the RR at 1.25, simulations of 10% increased sample sizes (N = 142,629) resulted in  powers of 70.7% (weighted) and 43.0% (inverse probability weighted). Simulations of 30% increased sample sizes (N = 168,562) resulted in powers of 73.9% (weighted) and 52.8% (inverse probability weighted). Simulations of 50% increased sample sizes (N = 194,495) resulted in powers of 79.8% (weighted) and 57.7% (inverse probability weighted). Simulations with 129,663 randomized visits with varying Ab+ prevalences by study arm, constrained by the aggregate Ab+ prevalences (5.9%, weighted; 4.7% inverse probability weighted) and their respective 95% lower confidence limits (LCLs) (5.1% and 3.3%, respectively), resulted in RRs of 1.35 (weighted) and 1.45 (inverse probability weighted) to achieve a power >80% (Figs. 4 and 5). Given the steep slope of the power curve between simulated trials of 0% and 100%, it was noted that small changes in the Ab+ prevalence difference would result in relatively large changes in trial power. As such, the decision was made to not change the planned enrollment target from the original calculations.

### Data collection methods

We will collect the following data for all eligible patients: (1) patient ED visit information (unique patient identifier, acuity level, mode of arrival, date/time of the visit); (2) demographics (age, gender, race, ethnicity, preferred language); (3) payer information (commercial, Medicare, Medicaid, self, or state assistance program); (4) details of randomization, including the intervention assigned and results of risk screening, if applicable; (5) whether a patient was offered, accepted, and completed rapid HCV testing; (6) results from all HCV Ab and RNA testing, if completed; (7) whether patients with detectable HCV RNA levels were successfully linked into care; and (8) components of the HCV care continuum. Data from (1) through (6) will be collected prospectively using methods developed by our team from each institution’s electronic screening, patient tracking, and laboratory reporting systems. We have long-standing and extensive experience interfacing with such systems across different institutions and obtaining large amounts of valid patient-level data [25]. Data from (7) and (8) will be retrospectively obtained using trained personnel and structured procedures.

As part of a sub-study to estimate risk among a representative sample of patients randomized to nontargeted screening, research assistants we will conduct surveys, using an electronic closed-response data collection instrument (REDCap, Vanderbilt University, TN), of patients enrolled into this specific study arm. No data or specimens will be collected for the current study or for future evaluation, including storage of biological specimens for genetic or molecular analyses.

### Data management

Denver Health will serve as the Data Coordinating Center (DCC) and data from non-Denver institutions will be transferred to the DCC using a Secure File Transfer Protocol (SFTP). Data will be extracted from each institution’s EHS and cleaned so that variables are consistent across sites. The cleaned and de-identified dataset will be sent via SFTP to the DCC for final data concatenation, cleaning, and analyses, performed using the most current version of SAS (SAS Institute, Inc., Cary, NC) (Figure S2).

### Statistical methods

After cleaning and locking the dataset, the primary analysis will be performed using the intention-to-treat principle while blinded to allocation. Continuous data will be reported as medians with interquartile ranges (IQRs) and categorical data as counts, proportions, and/or percentages with 95% confidence intervals (CIs). Bivariate statistical tests (e.g., Wilcoxon rank-sum test, Fisher’s exact test, etc.) will be used to compare variables between study groups. Patient-level data will be reported for all patient-level variables (e.g., age, sex/gender, race, ethnicity), although the primary unit of analysis will be patient visits. No interim analyses are planned given the pragmatic trial approach and minimal risk to subjects. Given the randomized design, the primary comparison will include an unadjusted RR for newly-identified HCV patients (primary outcome) with 95% CIs, specifically comparing nontargeted HCV screening to targeted HCV screening (primary hypothesis), while using a random effect hierarchical model to account for institution-level clustering, if present [26–28]. Sensitivity analyses will be performed to account for patients who were identified as Ab+ but RNA-, and subsequently determined to have been previously treated for HCV. Secondary comparisons will include all other outcomes by study arm, stratified by age, sex/gender, race, ethnicity, and institution. Statistical significance for the primary analysis will be defined as *p* <0.05 based on two-tailed statistical testing, which includes a lower 95% confidence limit of the RR >1.0. Although we do not anticipate significant missing data, multiple imputation will be used in instances where variables have >5% missingness. Although no formal interim analyses are planned for this trial, the study team may perform preliminary analyses for purposes of presentation at scientific meetings; these instances, if they occur, will be explicitly qualified as such and described as preliminary.

### Trial and data monitoring

In accordance with guidelines from the National Institutes of Health (NIH) and the primary funding agency, the National Institute on Drug Abuse (NIDA), we have developed a Data and Safety Monitoring (DSM) plan, and the principal investigators and core Denver-based research team will maintain appropriate oversight and monitoring of the trial’s conduct in its entirety, but in conjunction with site investigators. As this trial was not a phase 3 trial and that it was determined to be minimal risk to participants, no Data Safety Monitoring Board (DSMB) was required. The DSM plan was approved by the Colorado Multiple Institutional Review Board (COMIRB) and NIDA prior to initiating enrollment.

Data monitoring will be performed from the inception of participant enrollment at each site using data extracted from the EHS and transferred to the DCC using SFTP. The data manager will organize all data to understand total patient visits; inclusion and exclusion criteria; performance of Best Practice Advisories (BPAs) in Epic that were developed to facilitate enrollment and HCV screening; reasons for nursing actions related to BPAs; numbers related to HCV test offer, acceptance, and test completion by study arm; and aggregated test Ab and RNA results to ensure blinding. At the outset of enrollment, monitoring will occur nearly every day to ensure appropriate processes are occurring and to help guide trial performance. As our understanding of performance stabilizes, monitoring will be scaled back to a minimum of once weekly with frequent communication between the coordinating center and each site. Enrollment figures (e.g., modified CONSORT flow diagram, stacked bar graphs of enrollment by week of enrollment, stacked bar graphs of tests performed by study arm by week of enrollment, projected enrollment based on numbers of tests performed and estimated numbers of patients randomized, and trends of test acceptance by study arm) will be used to inform the study team’s understanding of trial performance and to inform any needed modifications while enrollment is occurring (Sample Monitoring Figures, Supplemental Appendix).

### Special consideration during the COVID-19 pandemic

This trial is fully integrated into standard emergency medical care and does not require adjunctive research personnel to facilitate enrollment. As such, no modifications to enrollment have occurred in the context of the COVID-19 pandemic. Ancillary data collection, including risk assessment of patients allocated to the nontargeted screening arm and time motion data collection (as part of the Cost Evaluation), were paused from March 13, 2020, through August 24, 2020, then again on December 2, 2020, through January 29, 2021, after which data collection resumed.

### Research ethics approval, consent, and confidentiality

For the Screening Trial and to enhance and streamline the Institutional Review Board (IRB) process, COMIRB, the primary IRB for Denver Health, serves as the central IRB for all sites, including Denver Health Medical Center, Johns Hopkins University, and the University of Mississippi Medical Center, with the latter two sites ceding to COMIRB. Institutional review board approval was originally granted on December 17, 2017.

Patients who present to the ED during trial performance will receive standard-of-care medical evaluation and treatment, may be asked questions about their risk for HCV, and may be offered, as voluntary and routine practice, rapid HCV testing using an opt-out consent mechanism. Consent for HCV testing will be integrated into routine emergency medical care as is currently standard. The medical care for those patients who do not receive HCV testing will not vary from those who do, except that among those who are tested the medical care team will know the patient’s HCV antibody test result and may alter the medical evaluation based on this additional information. Because HCV testing is a standard of care and because this trial will be evaluating two processes for performing HCV screening in this clinical setting (i.e., nontargeted vs. targeted screening), this potential change in care is consistent with current medical practice. We requested, and have obtained, a waiver of consent for everyone included in this trial based on 45 CFR 46.116(d)(1-4). We further obtained approval to obtain verbal consent for the risk assessment of a subgroup of patients randomized to the nontargeted study arm, and a non-human subject designation for time motion and cost data collected as part of the cost effectiveness evaluation. Thus, no specific consent forms exist for this trial.

We obtained a waiver of HIPAA authorization for all patients included in this study. Data collected as part of this project poses no more than a minimal risk of harm to privacy, and HIPAA authorization cannot be practicably carried out without a waiver due to the large number of patients planned for inclusion and because its requirement may bias participation. In addition, this research cannot be performed without specific requested protected health information (PHI). This project meets the requirements for a waiver of HIPAA authorization for the same reasons that requirements for a waiver of consent are met.

### Potential harms

Given the pragmatic nature of this trial and that all research components will be fully integrated into standard emergency care practice, the primary risks to patients included in this trial will be breach of confidentiality as all study procedures will be performed as routine medical care in the EDs or as follow-up for those who test positive for HCV Abs. Results of all HCV tests will be recorded in the patients’ medical records as part of standard medical care. Consent for the performance of rapid HCV testing will be integrated into the general ED medical consent for evaluation and treatment.

### Protocol amendments

Through February 17, 2022, 14 protocol amendments have occurred (Protocol, Supplemental Appendix).

### Access to data and dissemination policy

The primary results of this trial will be reported in accordance with CONSORT guidelines and published in a peer-reviewed journal. Authorship will conform to International Committee of Medical Journal Editors (ICMJE) guidelines. De-identified participant-level data and statistical code will be made available upon request to investigators outside the study team after standard data use agreements have been executed.

## Discussion

When complete, The DETECT Hep C Screening Trial will represent one of the largest pragmatic randomized clinical trials ever performed in EDs and will inform our understanding of the comparative effectiveness of two forms of HCV screening—nontargeted and targeted screening—in an emergency care environment when fully integrated into clinical care. The ultimate goal of this trial is to inform national policy related to HCV screening and by performing this trial in high volume, geographically diverse, urban EDs with likely varying HCV epidemiology, we have optimized generalizability to inform policy.

Rationale for the head-to-head comparison performed in this trial resulted from the prior recommendations from the CDC and USPSTF to perform targeted HCV screening [9, 10, 17]. However, pilot data and previous experience with nontargeted HIV screening has supported the potential effectiveness of nontargeted HCV screening [13]. Unfortunately, until now, no prospective comparative evaluation of nontargeted and targeted screening has been performed, particularly in an ED environment, and the findings will have important implications for practice and policy (i.e., national guidance for HCV screening in EDs).

All three institutions use Epic as their EHS, and this trial will use a functionality in Epic to perform real-time patient-level randomization and concealed allocation, a process that was successfully implemented in a similar multi-center pragmatic clinical trial comparing different forms of HIV screening in EDs [25]. This functionality is critical to successfully performing randomization on a 24-h basis by clinical staff in busy clinical environments as part of a pragmatic clinical trial.

As screening for HCV represents the intervention in this trial, all other processes leading to successful HCV testing will be fully integrated into clinical care. As such, we expect that numbers of test offers, acceptance, and completion may vary across sites and will vary within sites based on time of day, day of week, and surges of acutely ill or injured patients. The latter issue is a common challenge of conducting preventative screening in an ED setting, although its overall importance remains significant and further reinforces the importance of this trial.

Partnership with nurse leadership and nurse educators is critical to the implementation of any form of screening in the ED and is particularly important as it relates to implementation of screening as part of a clinical trial. Given the pragmatic nature of the study, however, it will be critical to provide sufficient education and oversight, while allowing processes to function, and possibly evolve during the course of enrollment, on their own. Striking this balance is critical to ensuring the results and inferences of the trial reflect actual practice to the extent possible. Enrollment monitoring will be critical to the performance of this trial to ensure optimal ongoing oversight while allowing both forms of screening to occur as they would in actual practice.

Potential limitations of this trial include its conduct at three academic medical centers with experience performing infectious diseases screening in the ED, although two of the centers were naïve to HCV screening at the outset. Furthermore, secular trends related to the diagnosis and treatment of HCV may influence numbers of patients identified with active HCV. Since planning and initiating enrollment in this trial, treatment of HCV has become more commonplace, less cost prohibitive, and more accessible. Finally, this trial began prior to the COVID-19 pandemic but has continued through the various waves, which may influence patient volumes and ED care.

## Trial status

This trial is ongoing with recruitment beginning on November 20, 2019, and continuing into 2023.

## Supplementary Information

**Additional file 1: Table S1.** Simulated results to estimate sample size and power using summary estimates weighted by contributions to enrollment form each site from the trial at 58.6% of target enrollment. Each simulation represents 1,000 simulated trials using Monte Carlo methods. **Table S2.** Simulated results to estimate sample size and power using summary estimates inverse probability weighted by contributions to enrollment from each site from the trial at 58.6% of target enrollment. Each simulation represents 1,000 simulated trials using Monte Carlo methods. **Table S3**. Two-way data tables of nontargeted versus targeted HCV antibody positive prevalence based on relative proportions of HCV tests performed in each of the two study arms. Light gray shaded values indicate combinations that contribute to an aggregate prevalence ranging from 5.1% to 6.8%, the 95% confidence intervals of the weighted aggregate Ab+ prevalence estimate. Dark gray shaded values indicate combinations that contribute to an aggregate prevalence of 4.7%, based on inverse probability weighting. **Figure S1.** Pragmatic-Explanatory Continuum Indicator Summary (PRECIS)-2 wheel diagram for The DETECT Hep C Screening Trial. **Figure S2.** Data components and flow of data for The DETECT Hep C Screening Trial. **Figure S3. A.** Eligibility Best Practice Advisory (BPA). **B.** Nontargeted HCV test offer BPA. **C.** Targeted HCV screening questions BPA. **D.** Targeted HCV test offer BPA. **Sample Monitoring Figures**. Protocol (Version 3.3, Date February 17, 2022).

## Data Availability

The investigators will have access to the final trial dataset, and the principal investigators, Drs. Haukoos and Rowan, take responsibility for the trial as a whole. Denver Health serves as the Clinical Coordinating Center and Data Coordinating Center.
