# Supplementary material for: The Determining Effective Testing in Emergency Departments and Care Coordination on Treatment Outcomes (DETECT) for Hepatitis C (Hep C) Screening Trial: rationale and design of a multi-center pragmatic randomized clinical trial of hepatitis C screening in emergency departments
Source: Trials. 2022 Apr 25;23:354. doi: 10.1186/s13063-022-06265-1 (PMC9036509; doi:10.1186/s13063-022-06265-1)
Supplement: Supplementary file 1 — Additional file 1: Table S1. Simulated results to estimate sample size and power using summary estimates weighted by contributions to enrollment form each site from the trial at 58.6% of target enrollment. Each simulation represents 1,000 simulated trials using Monte Carlo methods. Table S2. Simulated results to estimate sample size and power using summary estimates inverse probability weighted by contributions to enrollment from each site from the trial at 58.6% of target enrollment. Each simulation represents 1,000 simulated trials using Monte Carlo methods. Table S3. Two-way data tables of nontargeted versus targeted HCV antibody positive prevalence based on relative proportions of HCV tests performed in each of the two study arms. Light gray shaded values indicate combinations that contribute to an aggregate prevalence ranging from 5.1% to 6.8%, the 95% confidence intervals of the weighted aggregate Ab+ prevalence estimate. Dark gray shaded values indicate combinations that contribute to an aggregate prevalence of 4.7%, based on inverse probability weighting. Figure S1. Pragmatic-Explanatory Continuum Indicator Summary (PRECIS)-2 wheel diagram for The DETECT Hep C Screening Trial. Figure S2. Data components and flow of data for The DETECT Hep C Screening Trial. Figure S3. A. Eligibility Best Practice Advisory (BPA). B. Nontargeted HCV test offer BPA. C. Targeted HCV screening questions BPA. D. Targeted HCV test offer BPA. Sample Monitoring Figures. Protocol (Version 3.3, Date February 17, 2022). [file 13063_2022_6265_MOESM1_ESM.pdf]

SUPPLEMENTAL APPENDIX

The Determining Effective Testing in Emergency Departments and Care Coordination on Treatment Outcomes (DETECT) for Hepatitis C (Hep C) Screening Trial

Rationale and Design of a Multi-Center Pragmatic Randomized Clinical Trial of Hepatitis C Screening in Emergency Departments

Haukoos JS, Rowan RE, Galbraith JW, et al. for The DETECT Hep C Trials Investigators

1. **eTable 1:** Simulated results to estimate sample size and power using summary estimates weighted by contributions to enrollment form each site from the trial at 58.6% of target enrollment. Each simulation represents 1,000 simulated trials using Monte Carlo methods.
2. **eTable 2:** Simulated results to estimate sample size and power using summary estimates inverse probability weighted by contributions to enrollment from each site from the trial at 58.6% of target enrollment. Each simulation represents 1,000 simulated trials using Monte Carlo methods.
3. **eTable 3:** Two-way data tables of nontargeted versus targeted HCV antibody positive prevalence based on relative proportions of HCV tests performed in each of the two study arms. Light gray shaded values indicate combinations that contribute to an aggregate prevalence ranging from 5.1% to 6.8%, the 95% confidence intervals of the weighted aggregate Ab+ prevalence estimate. Dark gray shaded values indicate combinations that contribute to an aggregate prevalence of 4.7%, based on inverse probability weighting.
4. **eFigure 1:** Pragmatic-Explanatory Continuum Indicator Summary (PRECIS)-2 wheel diagram for The DETECT Hep C Screening Trial.
5. **eFigure 2:** Data components and flow of data for The DETECT Hep C Screening Trial.
6. **eFigure 3: A.** Eligibility Best Practice Advisory (BPA). **B.** Nontargeted HCV test offer BPA. **C.** Targeted HCV screening questions BPA. **D.** Targeted HCV test offer BPA.
7. **Sample Monitoring Figures**
8. **Protocol** (Version 3.3, Date February 17, 2022)

eTable 1. Simulated results to estimate sample size and power using summary estimates weighted by contributions to enrollment from each site from the trial at 58.6% of target enrollment. Each simulation represents 1,000 simulated trials using Monte Carlo methods.

Simulation	Nontargeted Ab+ Prevalence (%)	Targeted Ab+ Prevalence (%)	Δ (%)	Aggregate Ab+ Prevalence (%)	Randomized Visits	Median RR	Median NT Tests	Median T Tests	Median Total Tests	Power* (%)
Original	5	10	-5	6.7	50,000	0.91	3,839	2,130	5,969	0.3
1	9	1	+8	6.4	129,663	16.40	9,977	5,475	15,452	*100*
2	8	1	+7	5.7	129,663					
3	9	2	+7	6.7	129,663					
4	8	2	+6	6.0	129,663	7.35	10,091	5,511	15,602	*100*
5	7	2	+5	5.3	129,663					
6	8	3	+5	6.3	129,663					
7	7	3	+4	5.7	129,663	4.27	10,101	5,518	15,619	*100*
8	8	4	+4	6.7	129,663					
9	7	4	+3	6.0	129,663					
10	6	4	+2	5.3	129,663	2.73	10,100	5,514	15,614	*100*
11	7	5	+2	6.3	129,663					
12	6	5	+1	5.7	129,663					
13	7	6	+1	6.7	129,663	2.13	10,100	5,509	15,609	*100*
14	6	6	0	6.0	129,663	1.82	9,981	5,472	15,453	*100*
15	5	6	-1	5.3	129,663	1.53	10,101	5,515	15,616	*98.1*
16	6	7	-1	6.3	129,663	1.57	10,098	5,514	15,612	*99.5*
16a	5.3	7.2	-1.9	5.9	129,663	1.44	10,094	5,520	15,614	*82.3*
17	5	7	-2	5.7	129,663	1.31	10,099	5,517	15,616	71.0
18	6	8	-2	6.7	129,663	1.37	10,105	5,517	15,622	*91.4*
18a	5.5	8.0	-1.5	6.3	*194,495*	1.26	15,153	8,270	23,423	*79.8*
18b	5.5	8.0	-1.5	6.3	*168,562*	1.26	13,128	7,165	20,293	73.9
18c	5.5	8.0	-1.5	6.3	*142,623*	1.26	11,107	6,067	17,174	70.7
18d	5.5	8.0	-1.5	6.3	129,663	1.26	10,105	5,512	15,617	65.7
19	5	8	-3	6.0	129,663	1.14	10,091	5,512	15,603	26.6
20	4	8	-4	5.3	129,663	0.92	10,100	5,516	15,616	0.8
21	5	9	-4	6.3	129,663	1.02	10,100	5,517	15,617	4.0
22	4	9	-5	5.7	129,663	0.81	9,985	5,478	15,463	0
23	5	10	-5	6.7	129,663	0.92	10,104	5,519	15,623	0.1
24	4	10	-6	6.0	129,663					
25	3	10	-7	5.3	129,663					
26	4	11	-7	6.3	129,663					
27	3	11	-8	5.6	129,663					
28	4	12	-8	6.6	129,663					
29	3	12	-9	6.0	129,663					
30	2	12	-10	5.3	129,663	0.30	9,986	5,476	15,462	0

Abbreviations: HCV = hepatitis C; RR = risk ratio; NT = nontargeted; T = targeted

*Percentage of simulated trials where the lower 95% confidence limit of the RR was >1 (indicating statistical significance) when calculating the association between nontargeted HCV screening and new diagnoses when compared to targeted HCV screening.

eTable 2. Simulated results to estimate sample size and power using summary estimates inverse probability weighted by contributions to enrollment from each site from the trial at 58.6% of target enrollment. Each simulation represents 1,000 simulated trials using Monte Carlo methods.

Simulation	Nontargeted Ab+ Prevalence (%)	Targeted Ab+ Prevalence (%)	Δ (%)	Aggregate Ab+ Prevalence (%)	Randomized Visits	Median RR	Median NT Tests	Median T Tests	Median Total Tests	Power* (%)
Original	5	10	-5	6.7	50,000	0.91	3,839	2,130	5,969	0.3
1	6	2	+4	4.7	129,663	4.55	10,057	6,556	16,613	*100*
2	5	4	+1	4.7	129,663					
3	4.7	4.7	0	4.7	129,663	1.52	10,054	6,556	16,610	*91.4*
3a	4.7	4.7	0	4.7	*108,917*	1.54	8,450	5,505	13,955	*84.0*
4	4.6	4.8	-0.2	4.7	129,663	1.47	10,052	6,555	16,607	*85.1*
4a	4.6	4.8	-0.2	4.7	*108,917*	1.48	8,443	5,505	13,948	78.3
5	4.6	4.9	-0.3	4.7	129,663	1.44	10,061	6,552	16,613	79.2
6	4.5	5.2	-0.7	4.7	129,663	1.33	10,050	6,552	16,602	60.0
7	4.4	5.3	-0.9	4.7	*194,495*	1.25	15,053	8,523	23,606	
8	4.4	5.4	-1	4.7	*168,562*	1.25	13,074	7,213	20,287	57.7
8a	4.4	5.4	-1	4.7	*142,629*	1.25	10,060	9,830	19,890	52.8
8b	4.4	5.4	-1	4.7	129,663	1.25	11,059	6,558	17,617	43.0
9	4.3	5.5	-1.2	4.7	129,663	1.20	10,059	6,559	16,618	29.2
10	4	6	-2	4.7	129,663	1.02	10,055	6,561	16,616	2.9
11	3	8	-5	4.7	129,663					
12	2	10	-8	4.6	129,663					
13	1	12	-11	4.6	129,663	0.13	10,056	6,549	15,605	0

Abbreviations: HCV = hepatitis C; RR = risk ratio; NT = nontargeted; T = targeted

*Percentage of simulated trials where the lower 95% confidence limit of the RR was >1 (indicating statistical significance) when calculating the association between nontargeted HCV screening and new diagnoses when compared to targeted HCV screening.

eTable 3. Two-way data tables of nontargeted versus targeted HCV antibody positive prevalence based on relative proportions of HCV tests performed in each of the two study arms. Light gray shaded values indicate combinations that contribute to an aggregate prevalence ranging from 5.1% to 6.8%, the 95% confidence intervals of the weighted aggregate Ab+ prevalence estimate. Dark gray shaded values indicate combinations that contribute to an aggregate prevalence of 4.7%, based on inverse probability weighting.

		Targeted Prevalence											
		1%	2%	3%	4%	5%	6%	7%	8%	9%	10%	11%	12%
Nontargeted Prevalence	1%	1.0%	1.3%	1.7%	2.0%	2.3%	2.7%	3.0%	3.3%	3.6%	4.0%	4.3%	4.6%
	2%	1.7%	2.0%	2.3%	2.7%	3.0%	3.3%	3.7%	4.0%	4.3%	4.6%	5.0%	5.3%
	3%	2.3%	2.7%	3.0%	3.3%	3.7%	4.0%	4.3%	4.7%	5.0%	5.3%	5.6%	6.0%
	4%	3.0%	3.3%	3.7%	4.0%	4.3%	4.7%	5.0%	5.3%	5.7%	6.0%	6.3%	6.6%
	5%	3.7%	4.0%	4.3%	4.7%	5.0%	5.3%	5.7%	6.0%	6.3%	6.7%	7.0%	7.3%
	6%	4.3%	4.7%	5.0%	5.3%	5.7%	6.0%	6.3%	6.7%	7.0%	7.3%	7.7%	8.0%
	7%	5.0%	5.3%	5.7%	6.0%	6.3%	6.7%	7.0%	7.3%	7.7%	8.0%	8.3%	8.7%
	8%	5.7%	6.0%	6.3%	6.7%	7.0%	7.3%	7.7%	8.0%	8.3%	8.7%	9.0%	9.3%
	9%	6.4%	6.7%	7.0%	7.3%	7.7%	8.0%	8.3%	8.7%	9.0%	9.3%	9.7%	10.0%
	10%	7.0%	7.4%	7.7%	8.0%	8.3%	8.7%	9.0%	9.3%	9.7%	10.0%	10.3%	10.7%
	11%	7.7%	8.0%	8.4%	8.7%	9.0%	9.3%	9.7%	10.0%	10.3%	10.7%	11.0%	11.3%
	12%	8.4%	8.7%	9.0%	9.4%	9.7%	10.0%	10.3%	10.7%	11.0%	11.3%	11.7%	12.0%

Abbreviation: HCV = hepatitis C.

eFigure 1. Pragmatic-Explanatory Continuum Indicator Summary (PRECIS)-2 wheel diagram for The DETECT Hep C Screening Trial.

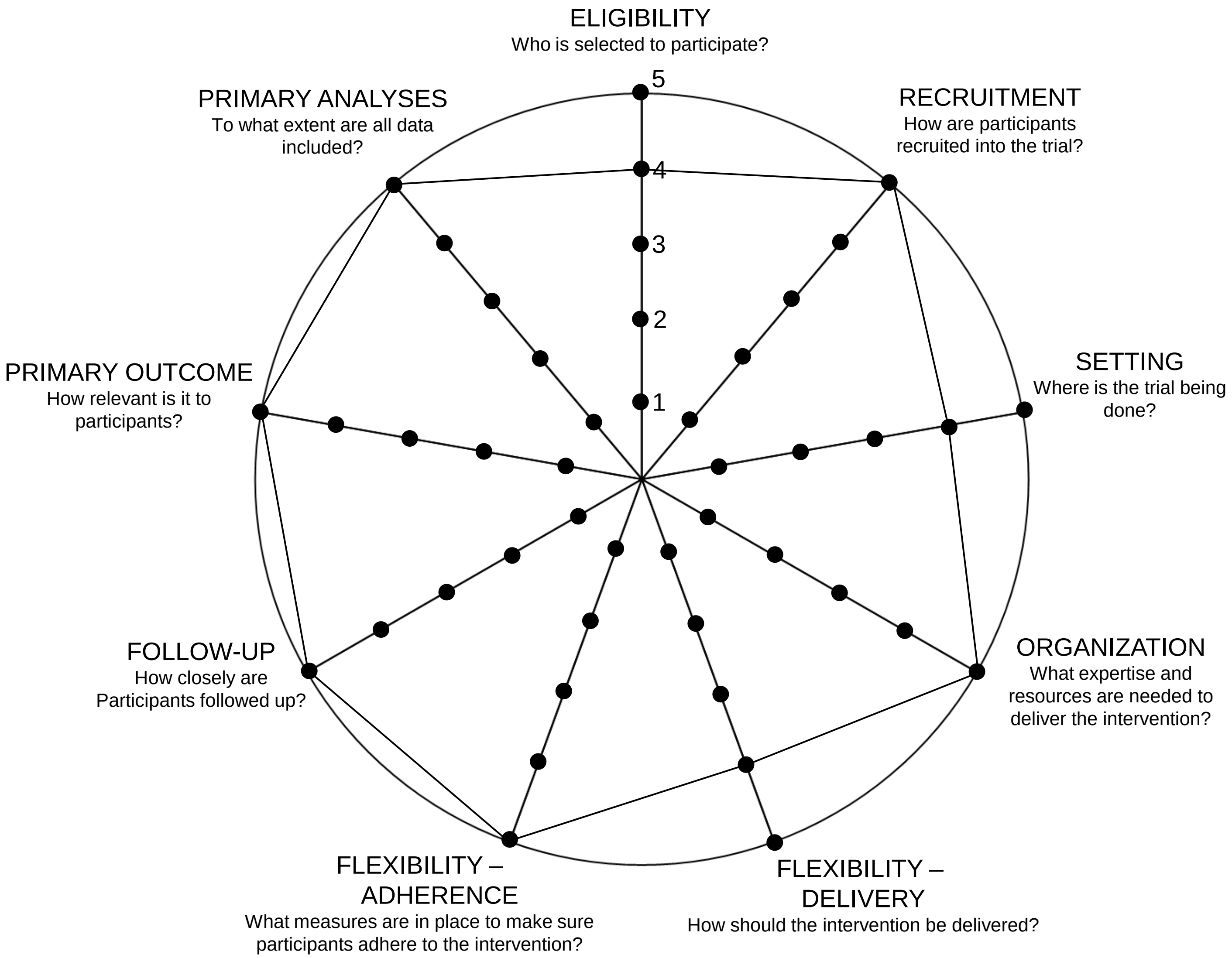

eFigure 2. Data components and flow of data for The DETECT Hep C Screening Trial. *Performed by trained research assistants using a structured approach and electronic data collection procedures in REDCap (REDCap, Vanderbilt, TN).

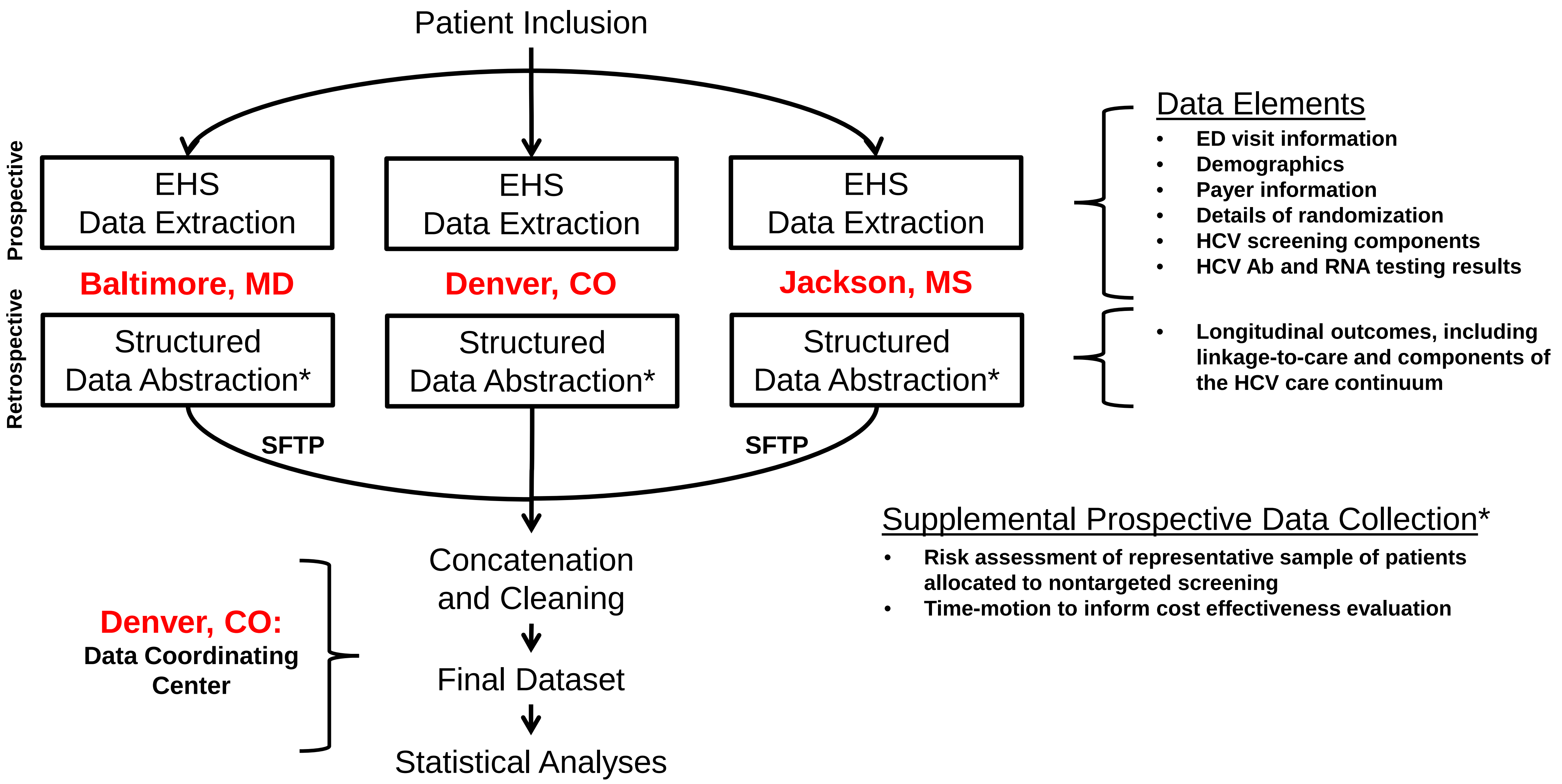

eFigure 3A. Eligibility Best Practice Advisory (BPA).

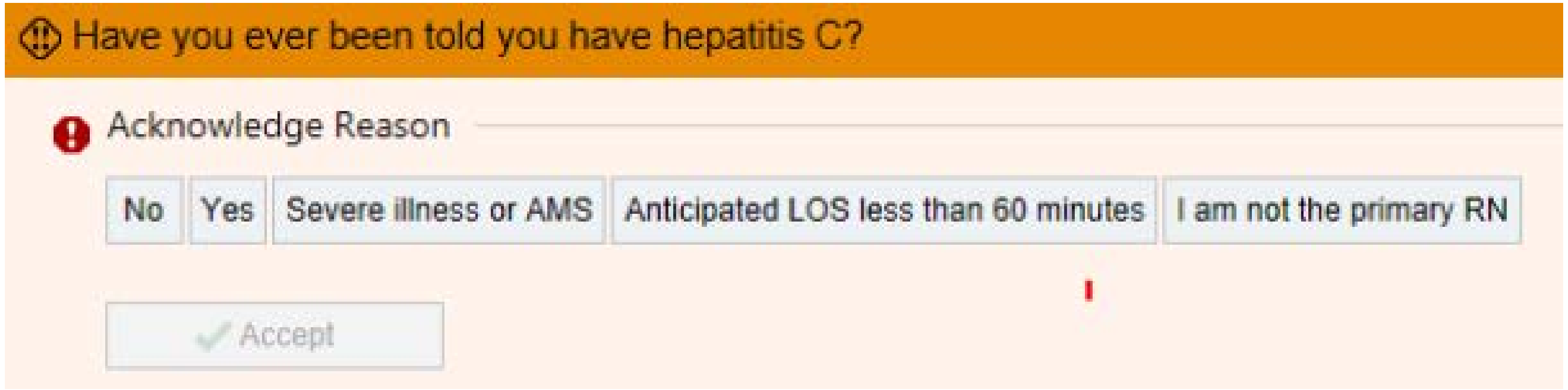

eFigure 3B. Nontargeted HCV test offer BPA.

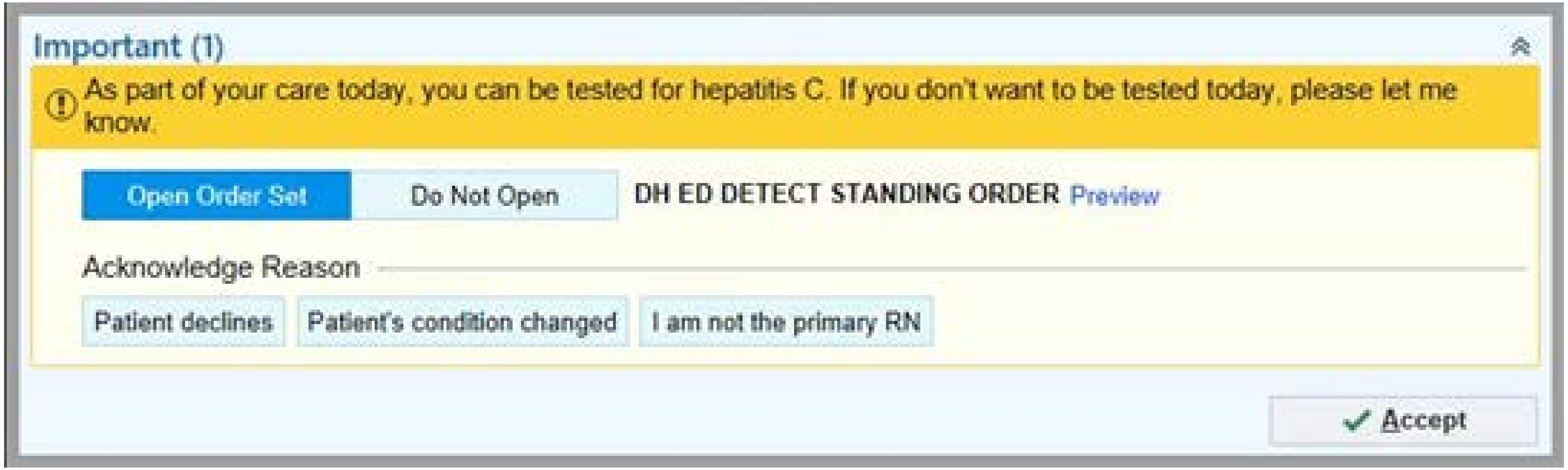

eFigure 3C. Targeted HCV screening questions BPA.

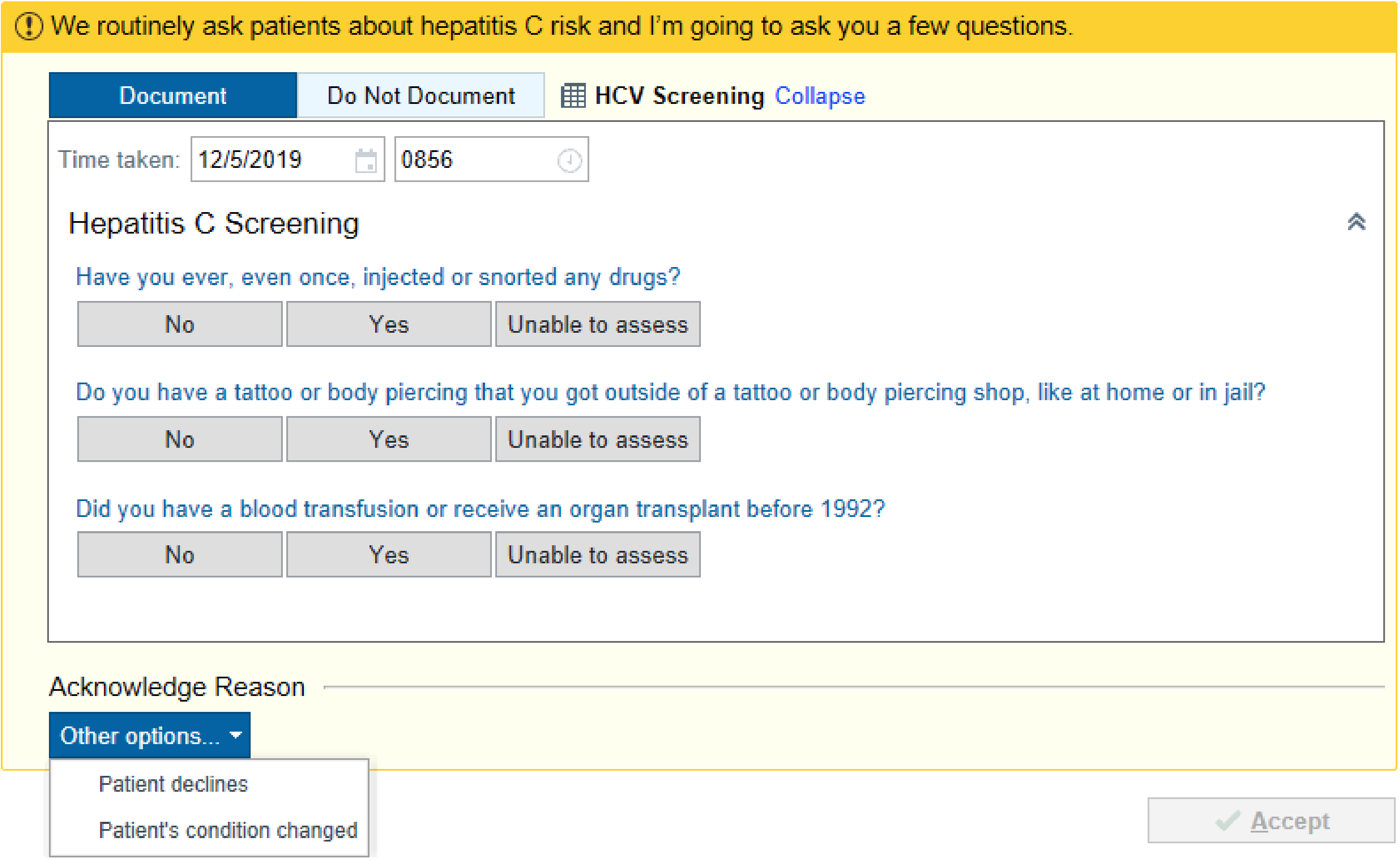

eFigure 3D. Targeted HCV test offer BPA.

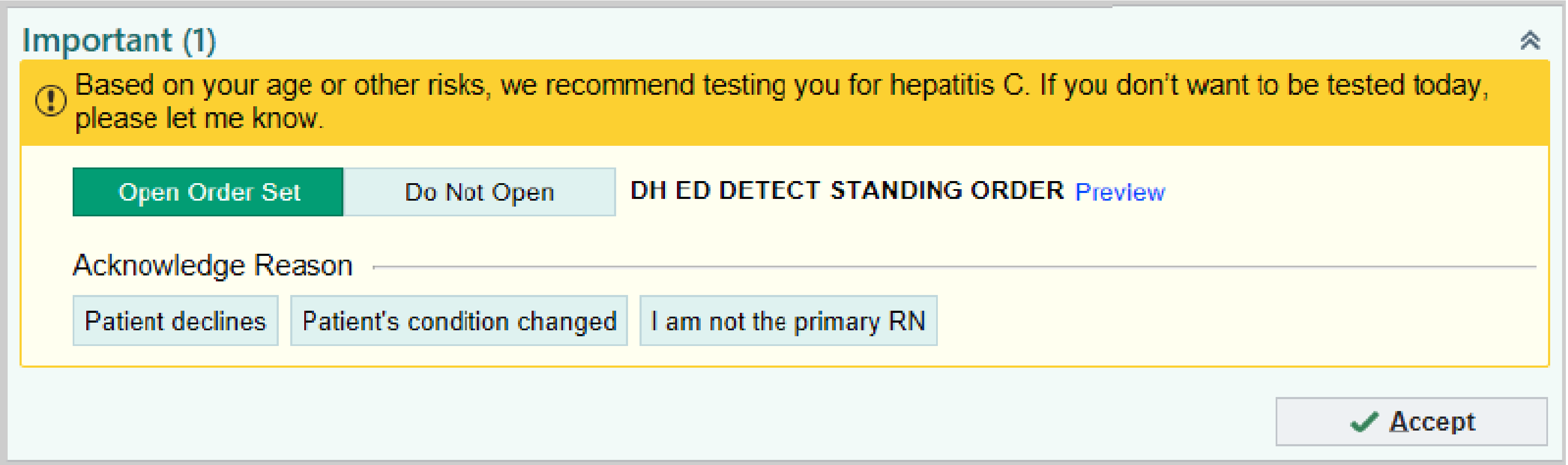

Figure. DETECT Data Monitoring, All clinical areas; November 20 2019 – July 5 2021

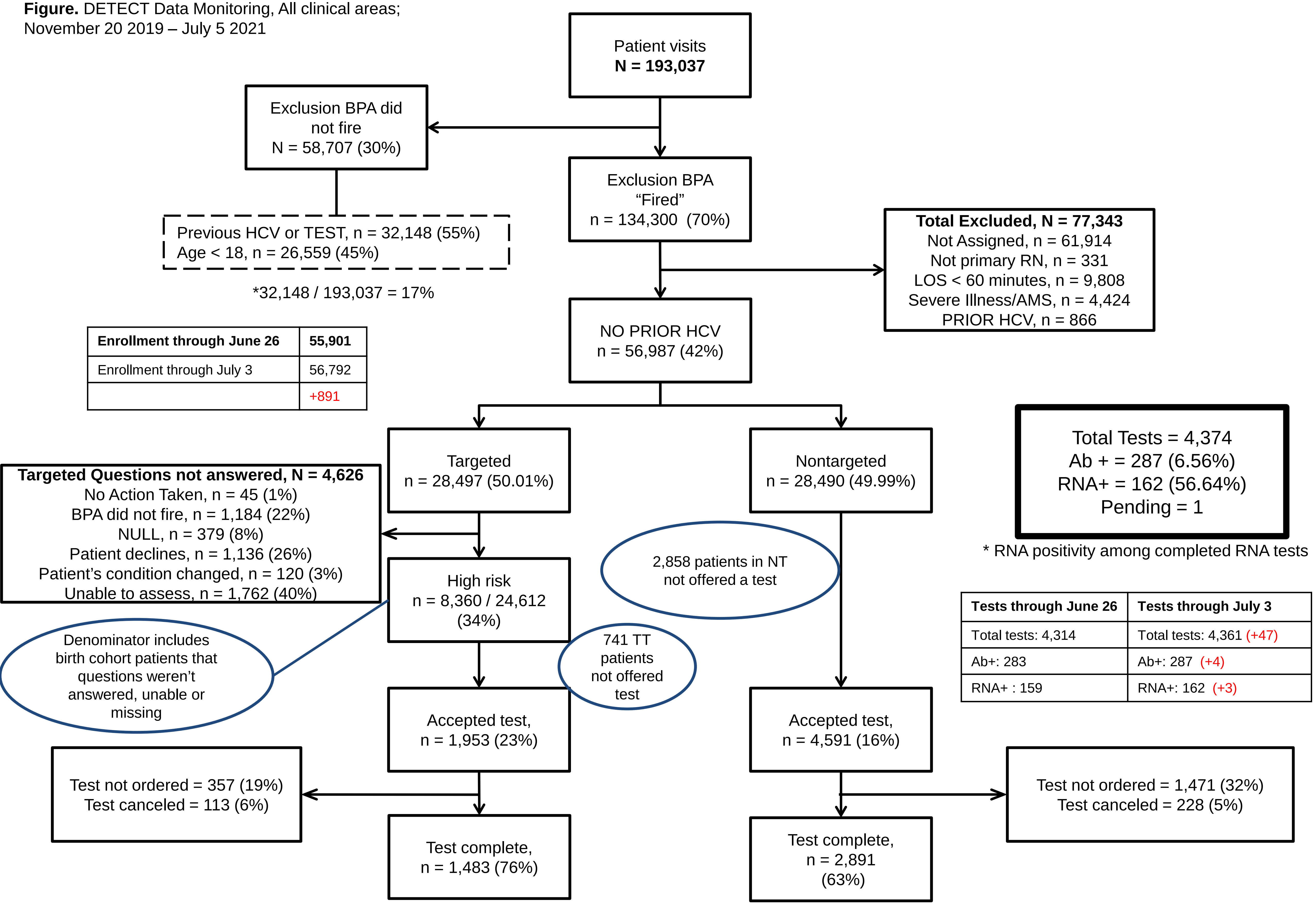

**Enrollment through June 26**	**55,901**
Enrollment through July 3	56,792
	+891

Tests through June 26	Tests through July 3
Total tests: 4,314	Total tests: 4,361 (+47)
Ab+: 283	Ab+: 287 (+4)
RNA+ : 159	RNA+: 162 (+3)

Figure. Number of patients by category per week, all clinical areas

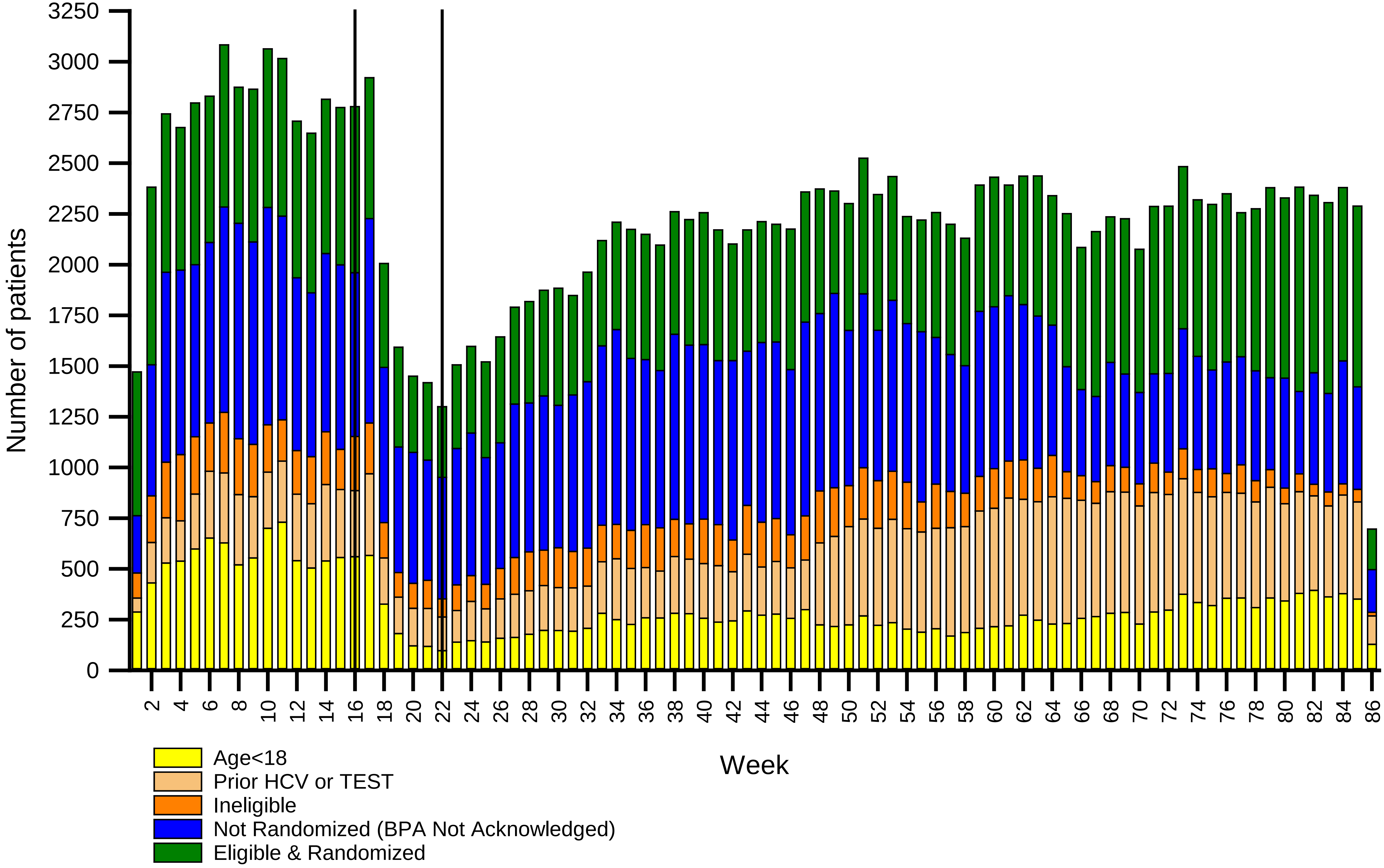

Figure. Number of eligible & randomized patients per week, all clinical areas

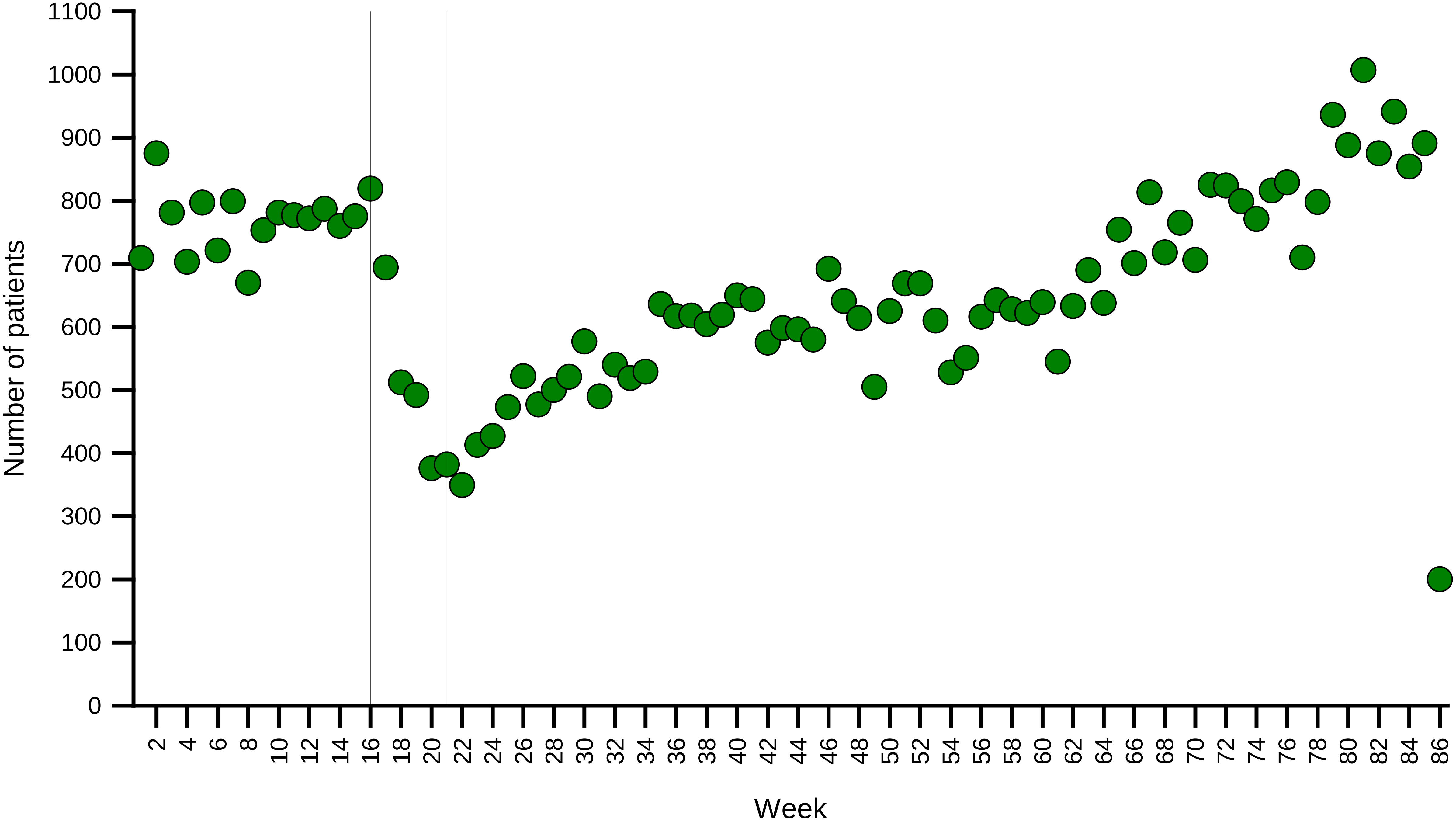

Figure. Percent of total number of patients "Not Assigned".
Daily slope prior week plus partial week = 1.19 (previously 0.08)

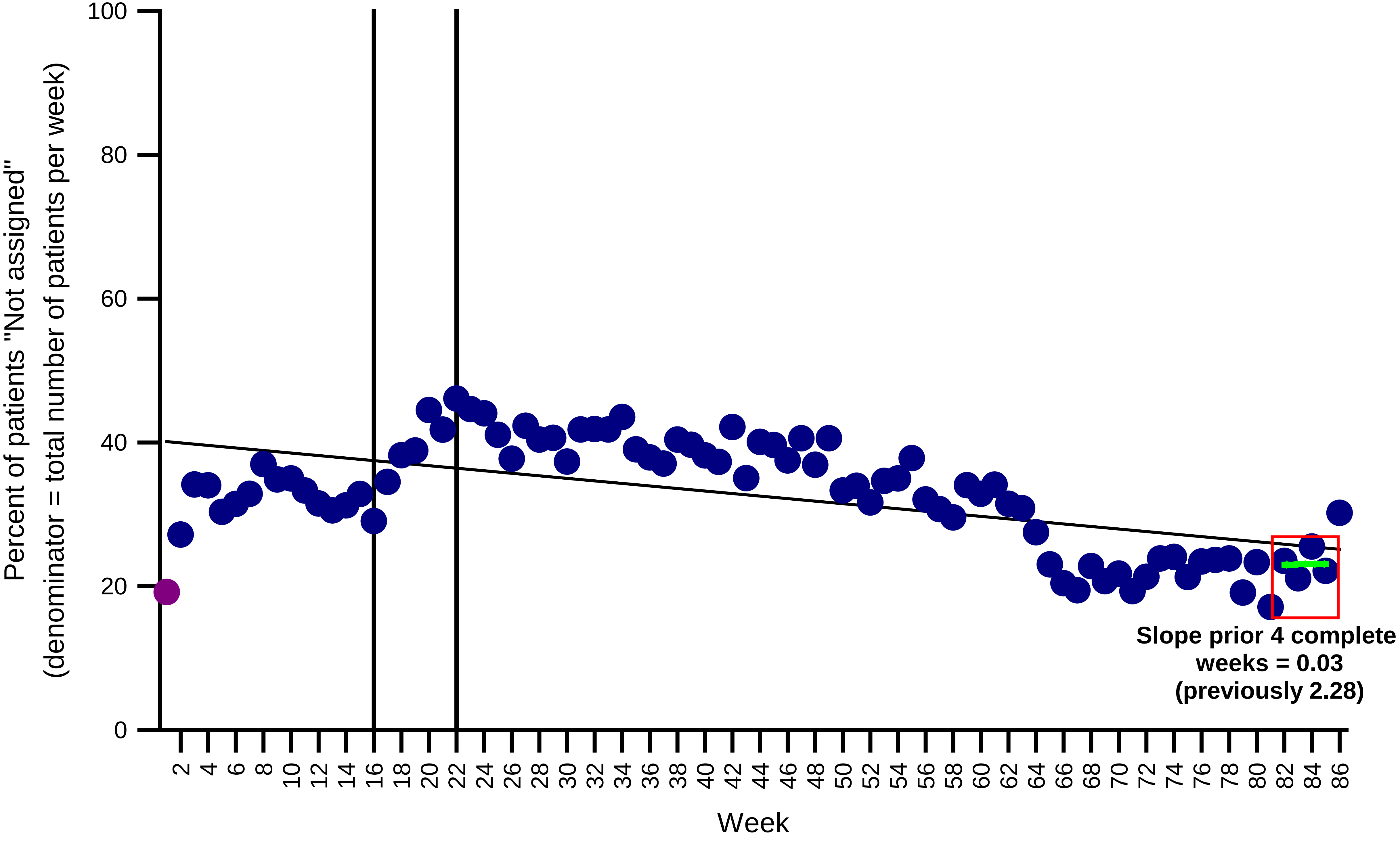

Figure. Number of tested patients per week across all clincal areas

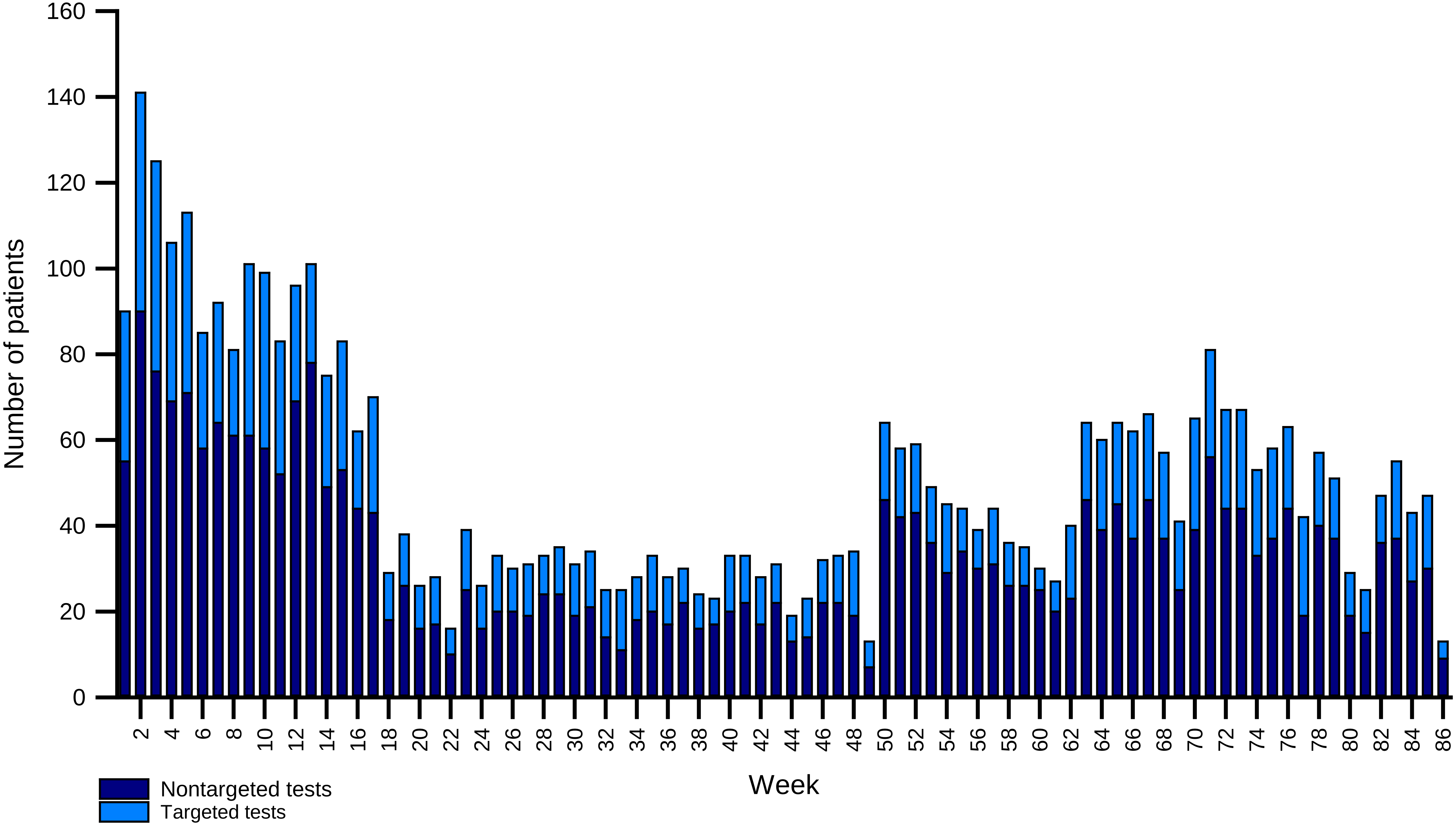

Figure. Number of tested patients per week across all clincal areas

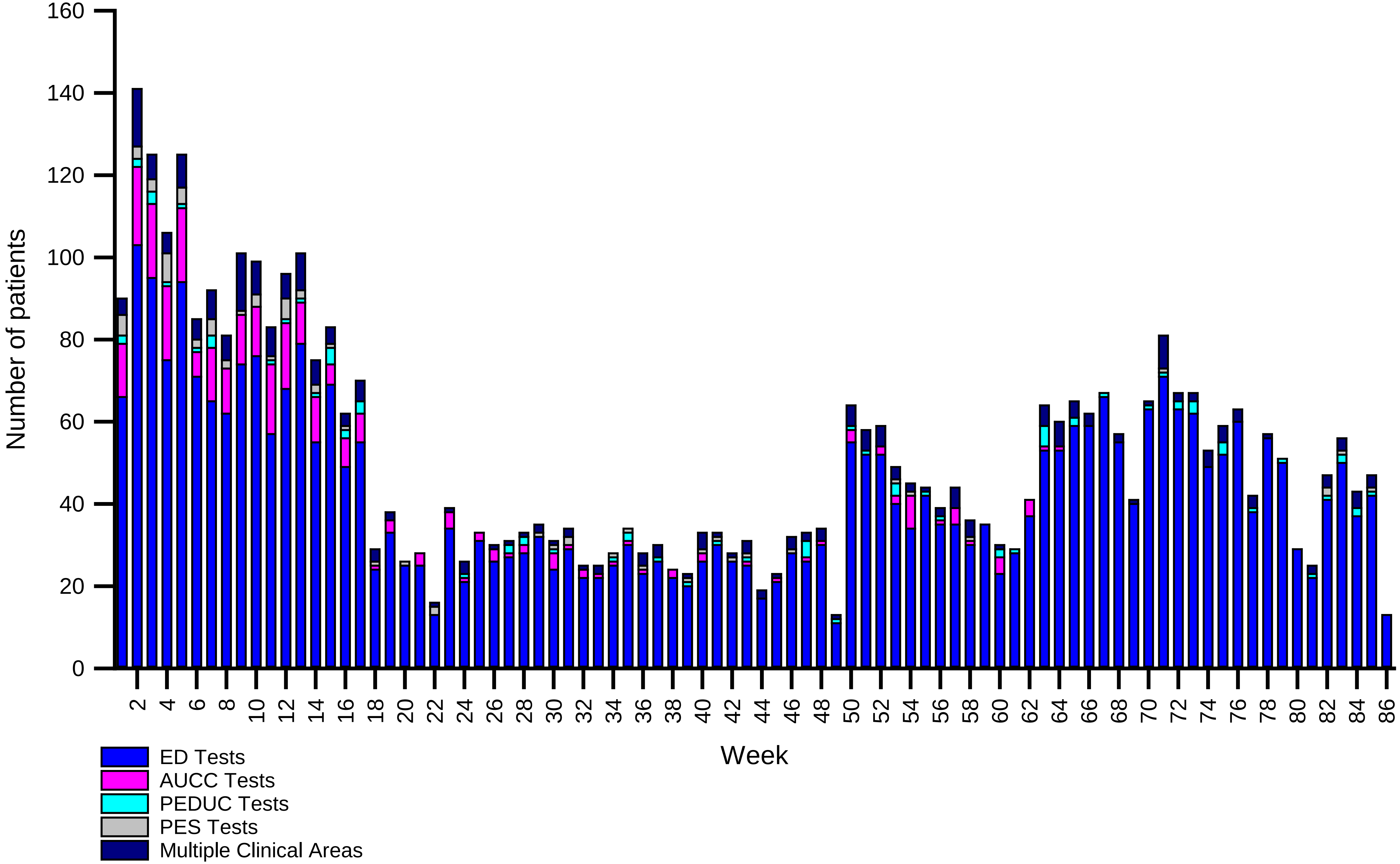

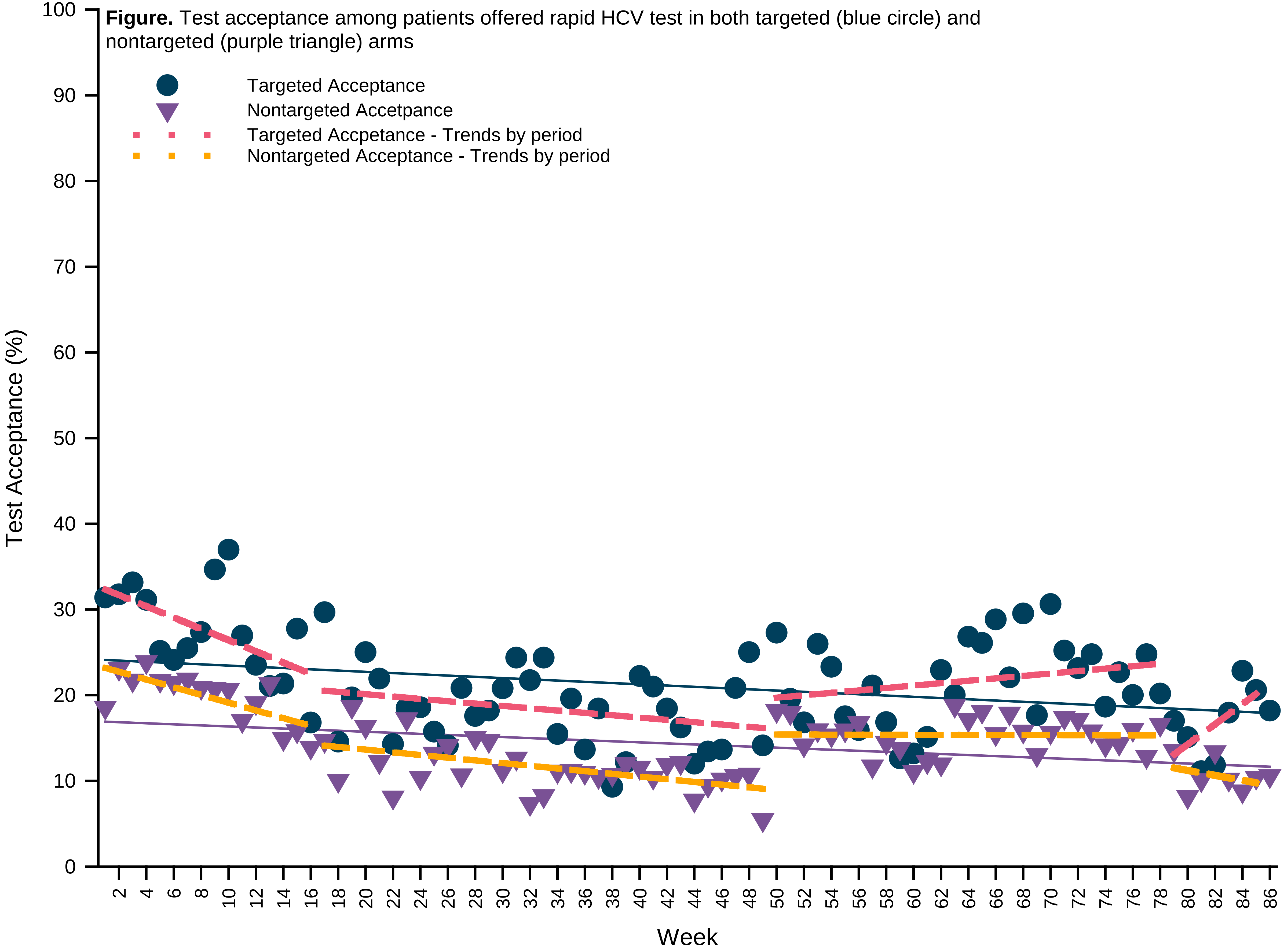

Figure. Test acceptance among patients offered rapid HCV test in both targeted (blue circle) and nontargeted (purple triangle) arms

Figure. Estimated amount of time to reach 4,655 completed screening tests and estimated number of patients randomized.

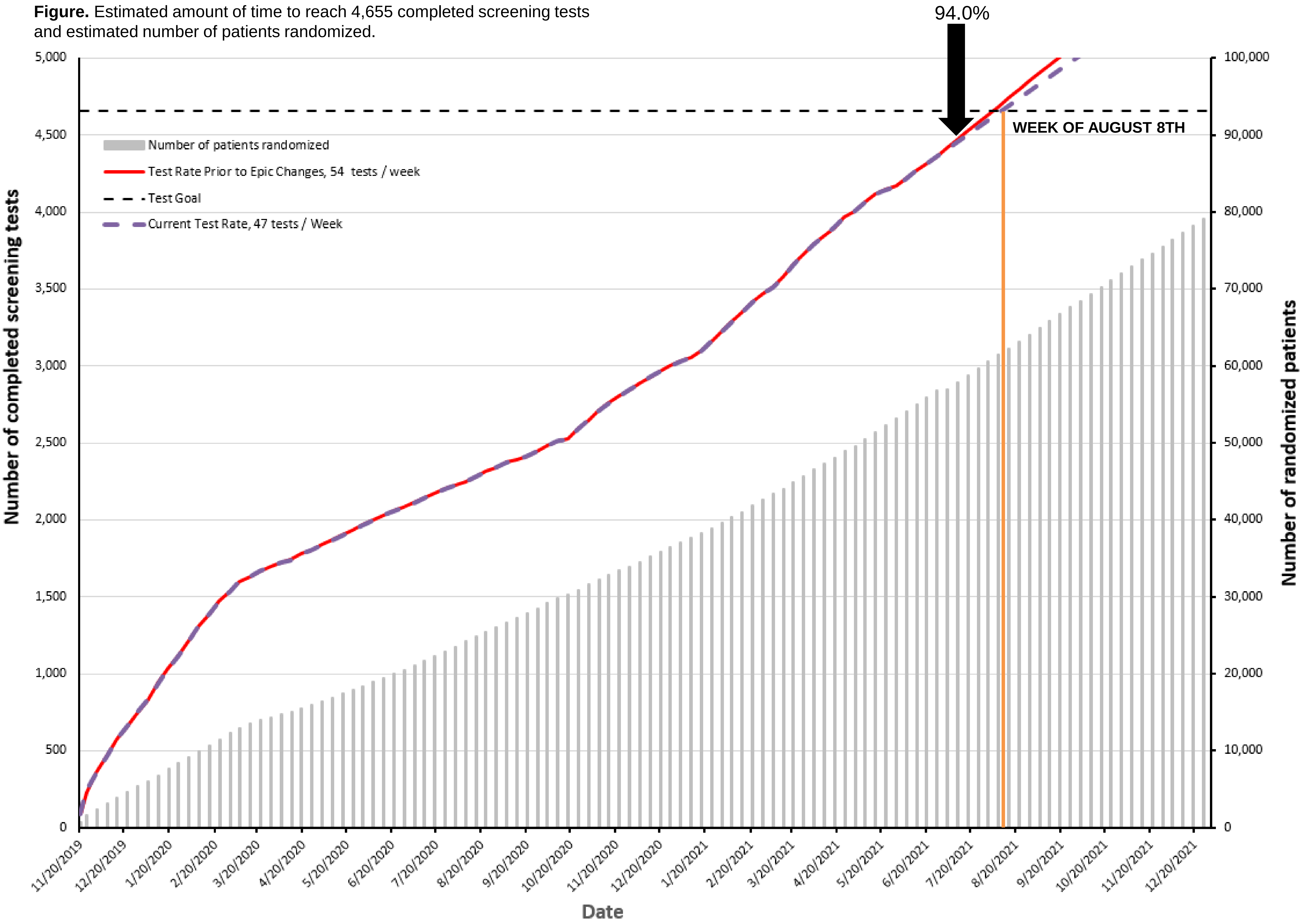

Figure. Number of patients by category per week, Adult Emergency Department

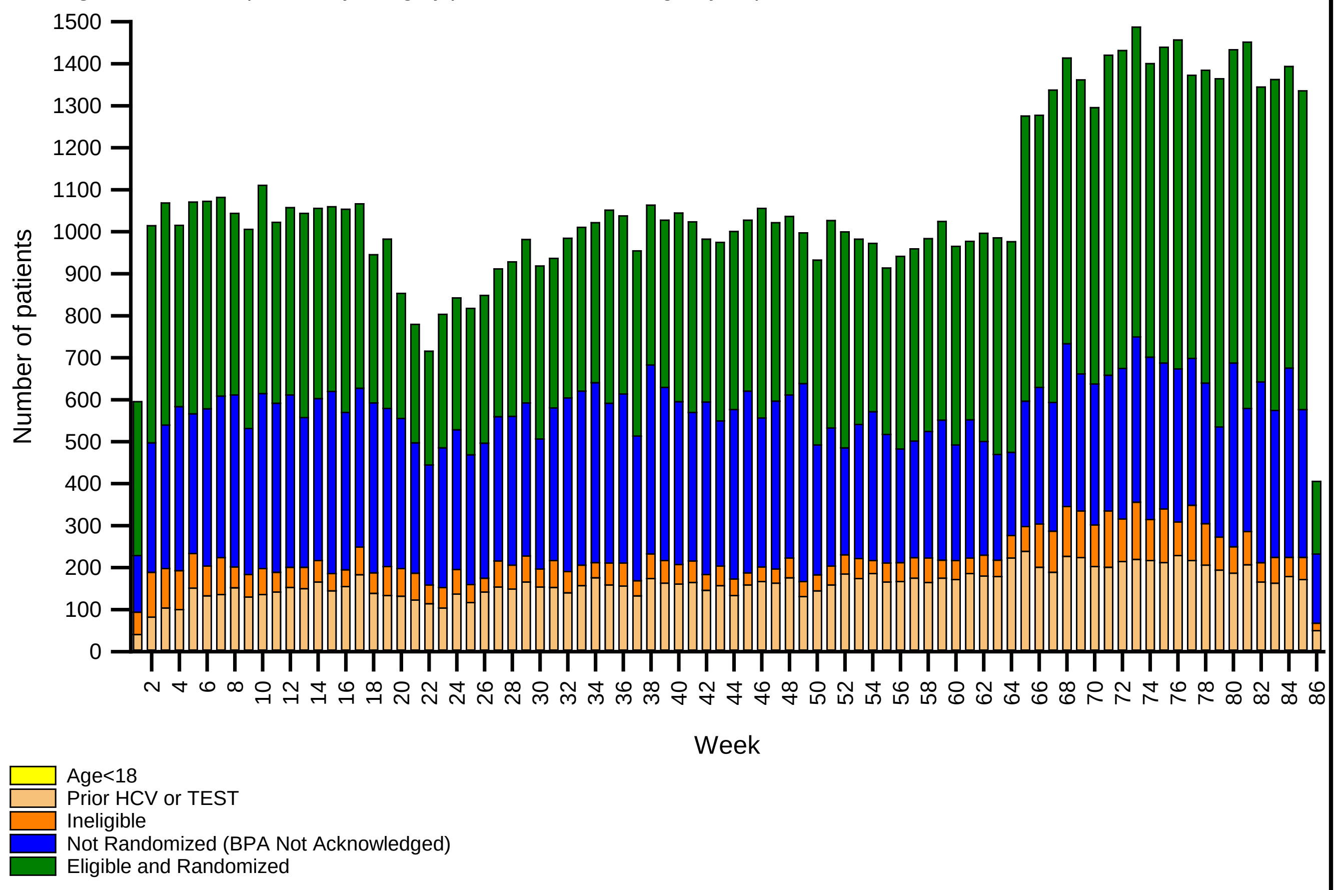

Figure. Percentage of total number of patients "Not Assigned", Adult ED

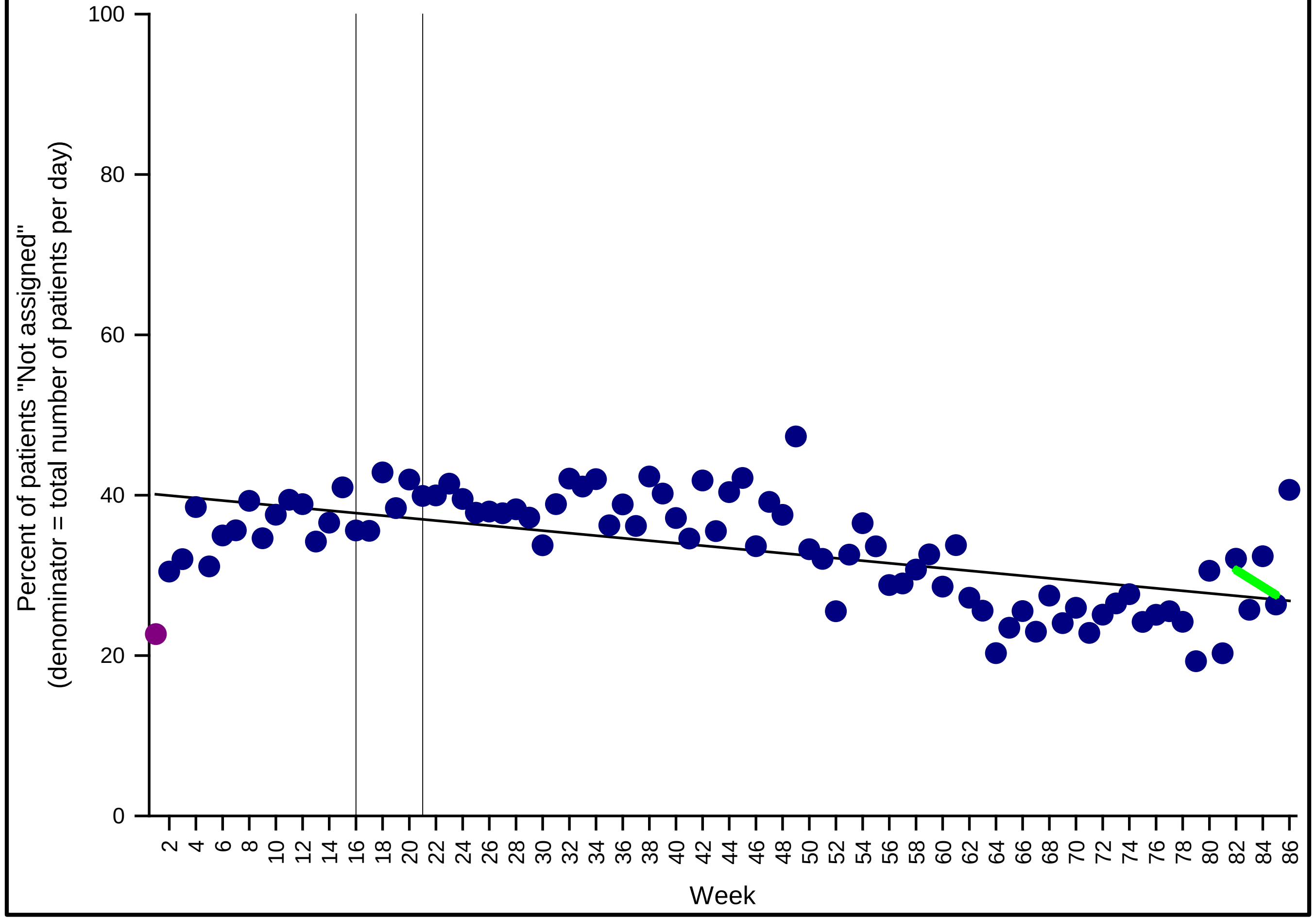

Figure. Number of patients by category per week, PEDUC

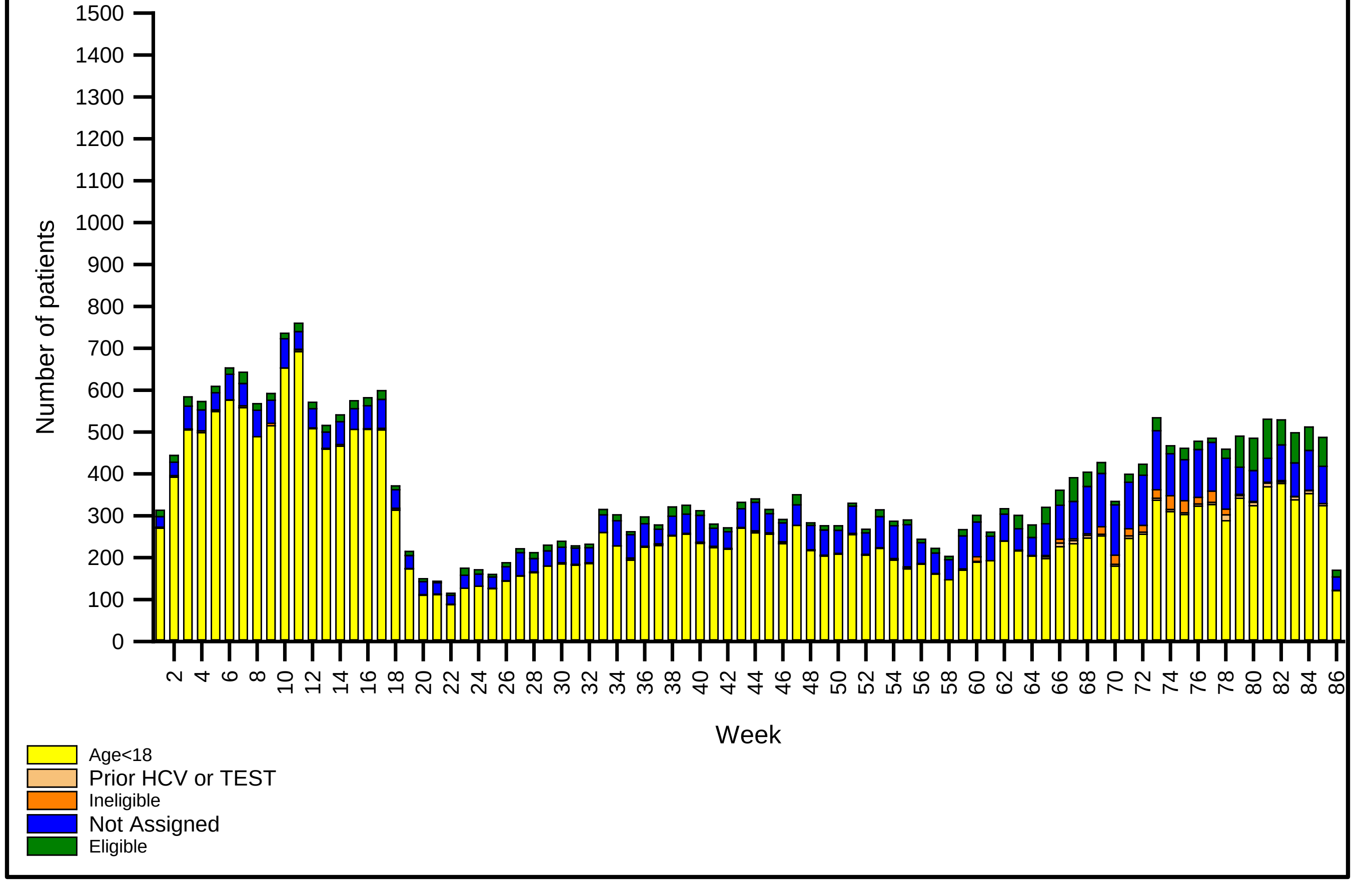

Figure. Percentage of total number of patients "Not Assigned", PEDUC

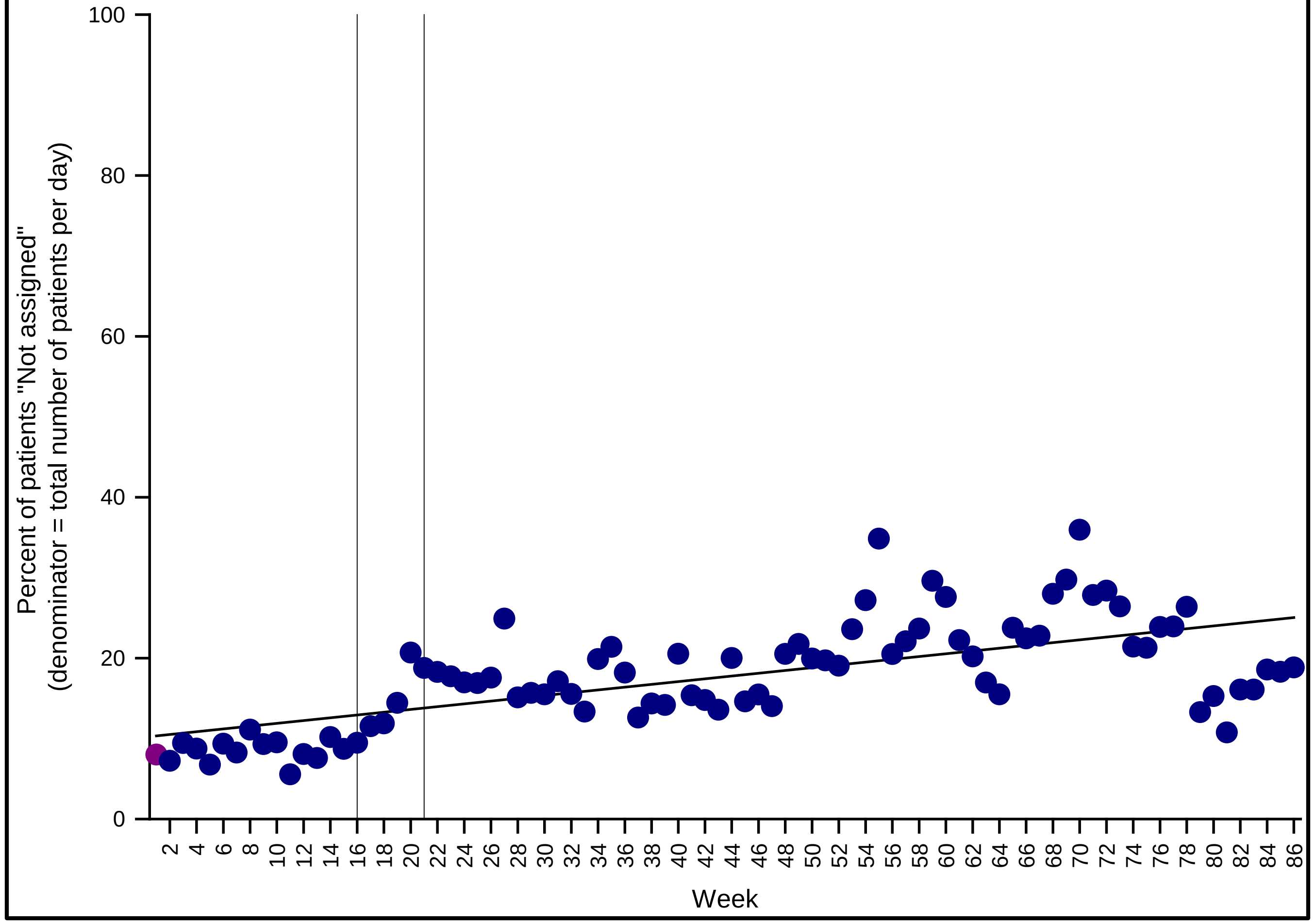

Figure. Number of patients by category per week, PES

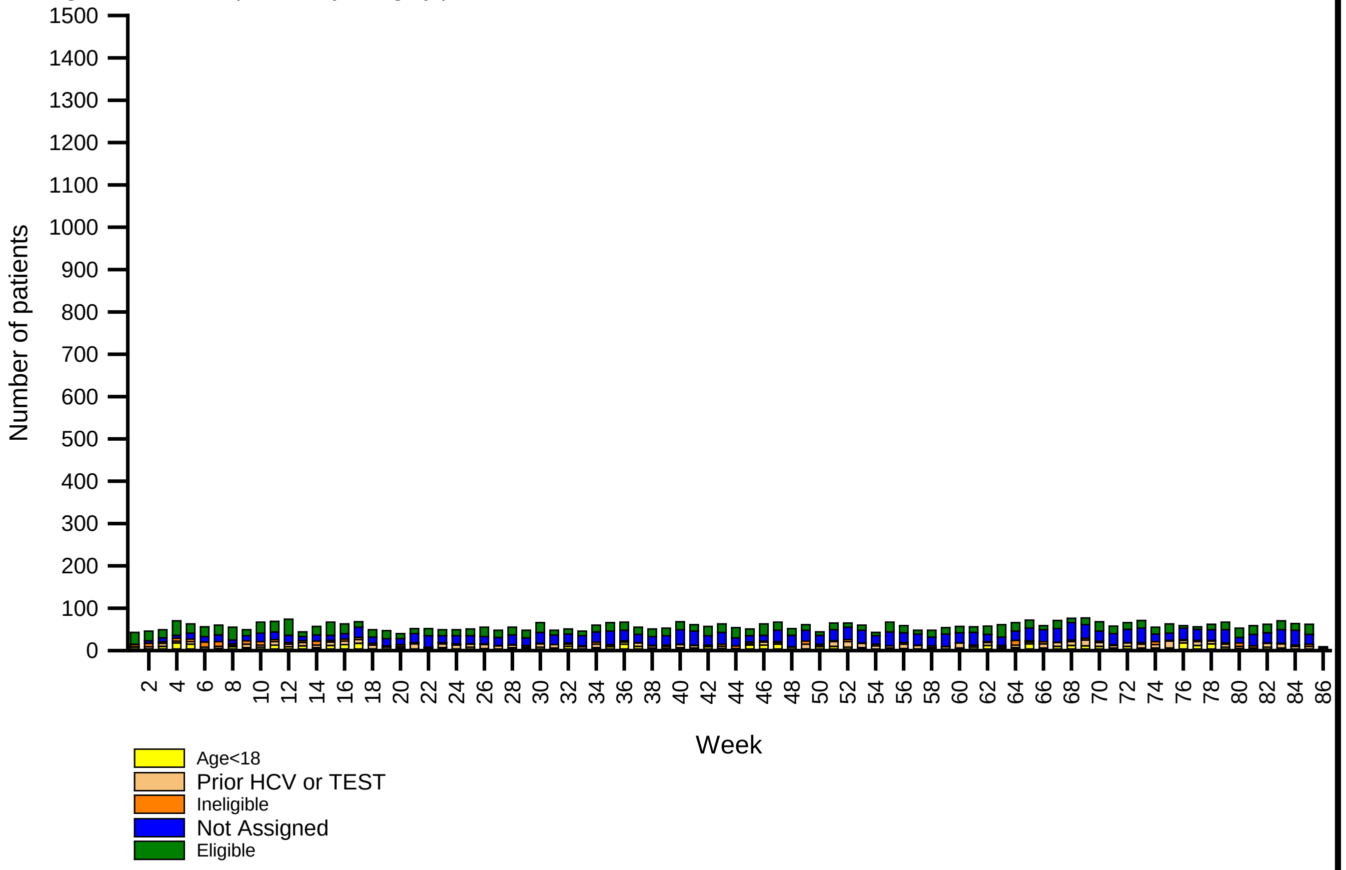

Figure. Percentage of total number of patients "Not Assigned", PES

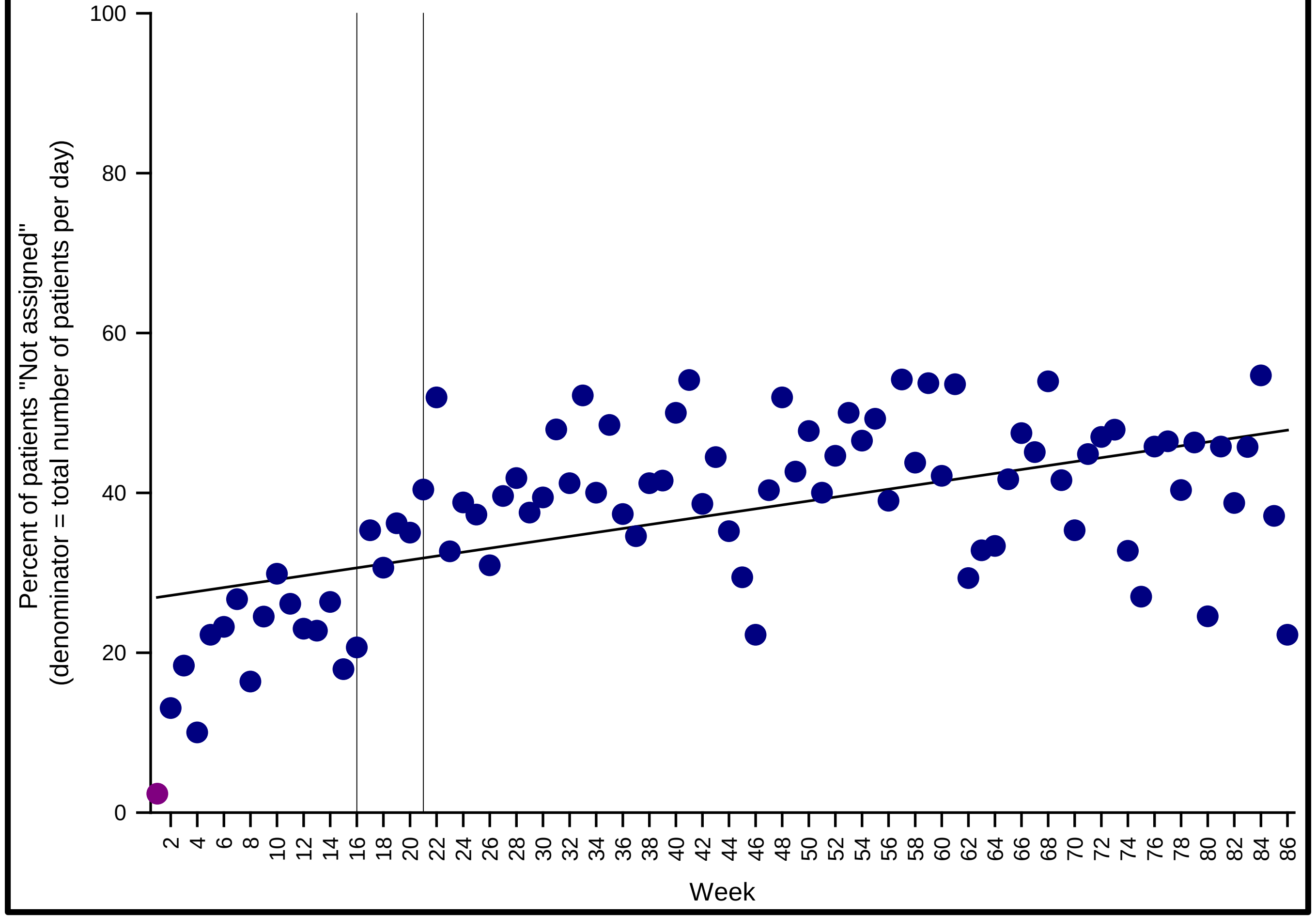

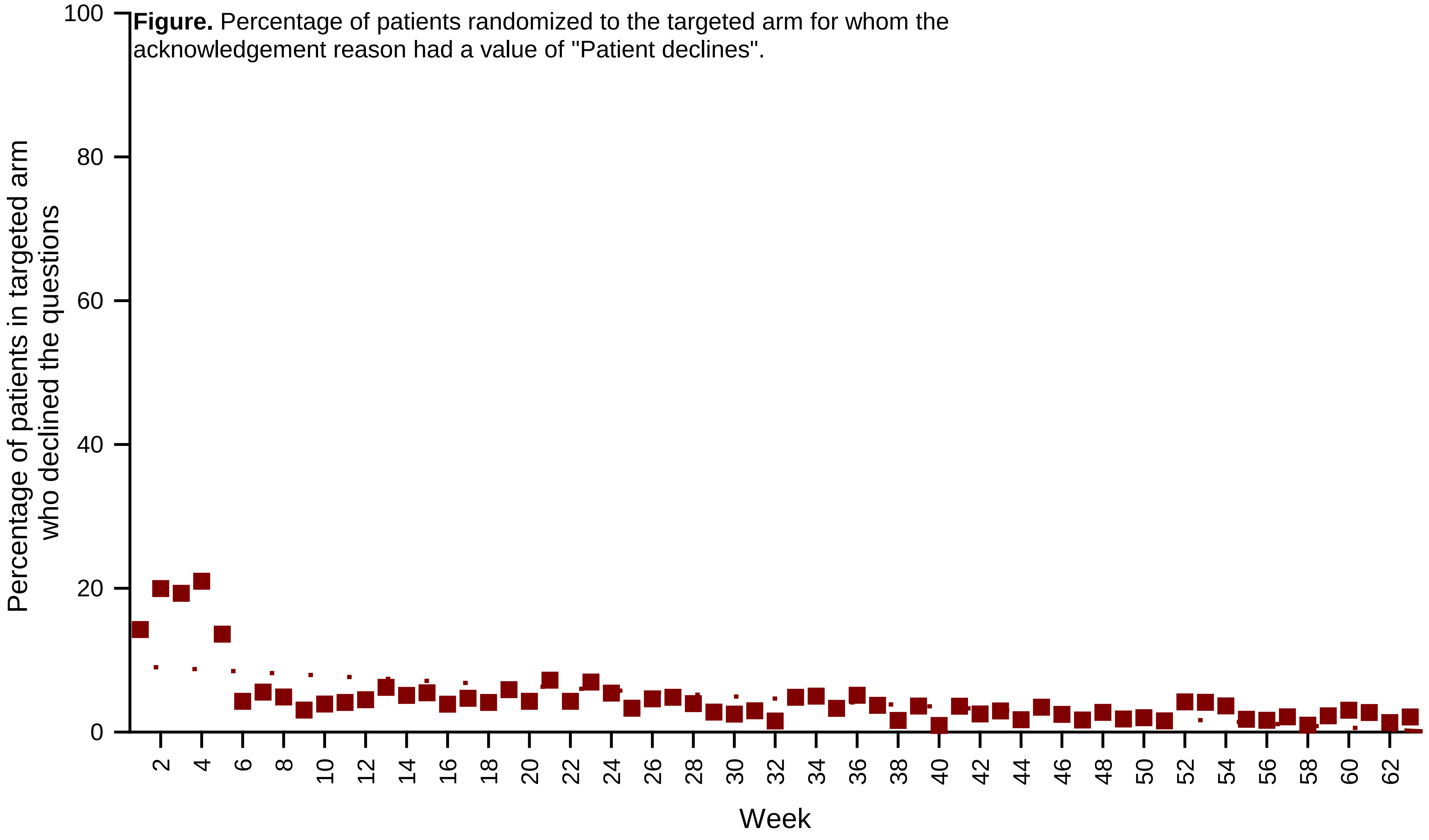

Figure. Percentage of patients randomized to the targeted arm for whom the acknowledgement reason had a value of "Patient declines".

Figure. Positive rapid HCV antibody test results & housing status of patient, per hour of day (based on hour that test results appear in chart), all clinical areas

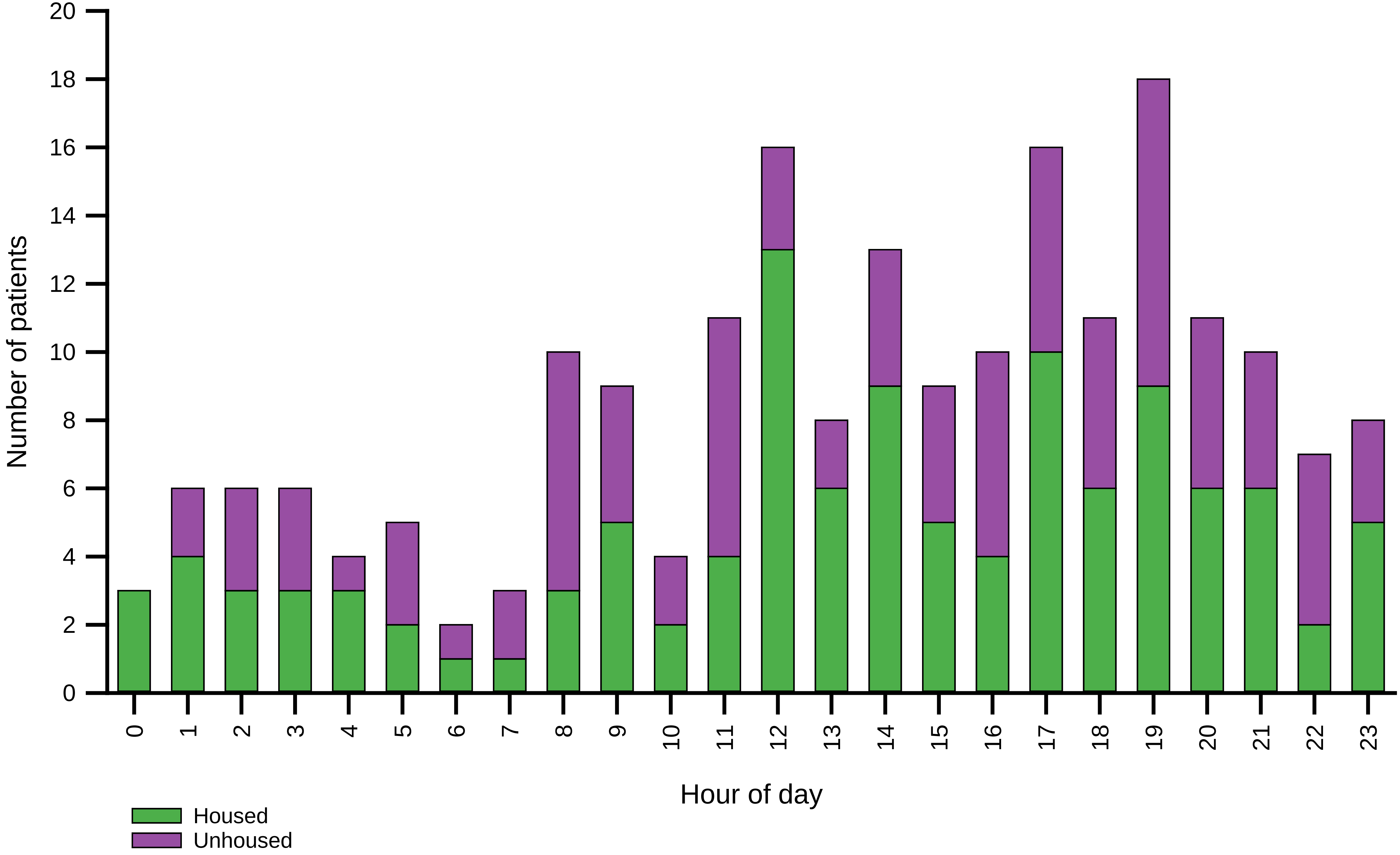

Figure. Percentage of patients randomized to each arm, all clinical areas.

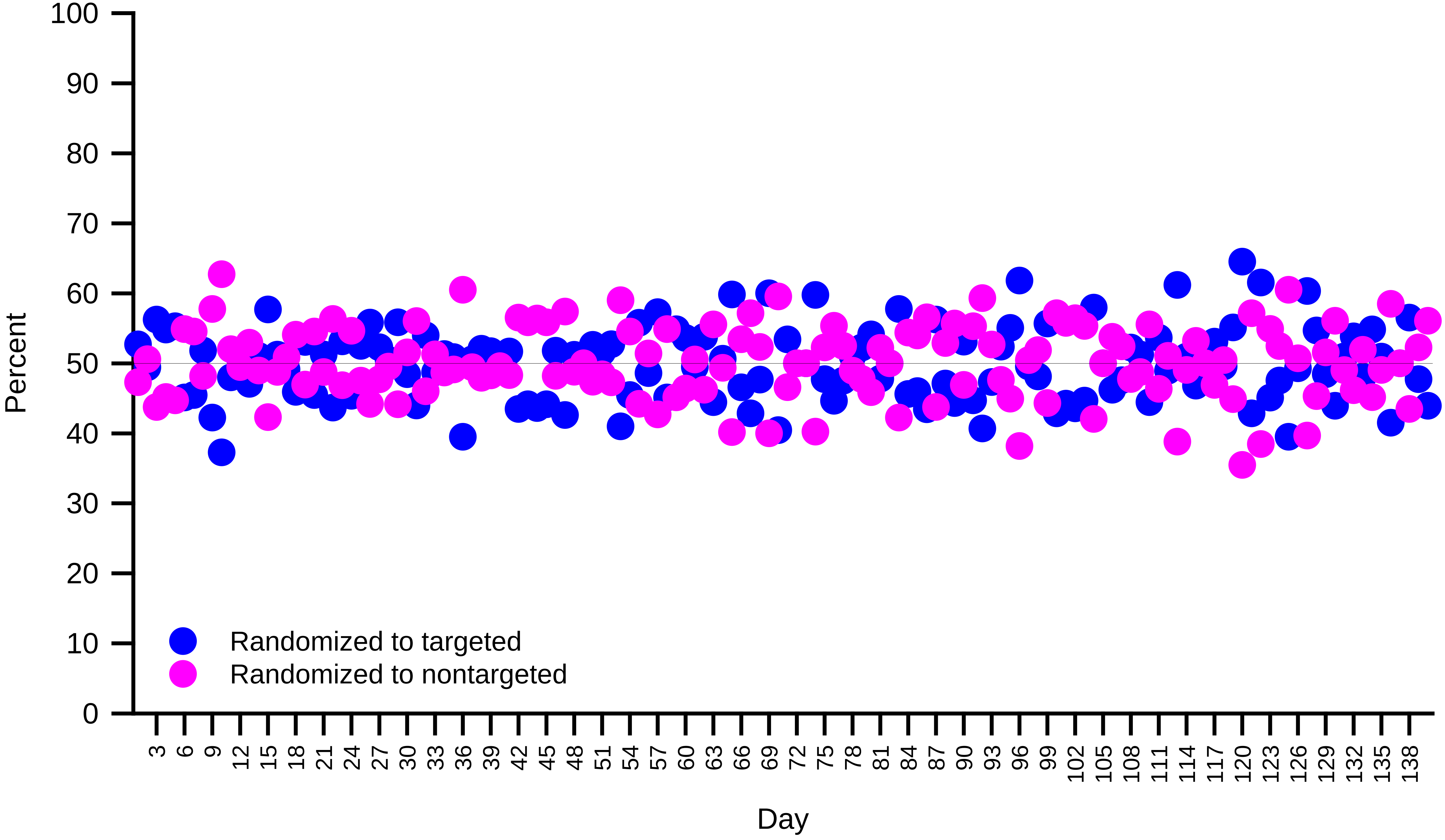

Table. Risk characteristics of patients randomized to targeted arm and completed questions.

	N	(%)	Test Acceptance
Number randomized to Targeted	5,421		
Questions completed	4,541	(84)	
High Risk	1,696	(37)	
Birth Cohort Only	839	(49)	17%
Birth Cohort + Other Risk	161	(10)	43%
Other Risk Only	690	(41)	42%

	**Day Shift (7a – 7p)**		**Night Shift (7p – 7a)**	
	ED	AUCC	ED	AUCC
**Total number of patients**	3,265	3,989	2,343	34
**Total number of "not assigned"**	981	1,807	846	17
**Percentage "not assigned"**	30.0%	45.2%	36.1%	50.0%

	**ED N = 1,234**		**AUCC N = 1,292**	
	N	(%)	N	(%)
**Arrival**				
Ambulance	422	(34)	25	(2)
Self	794	(64)	1,267	(98)
**Acuity**				
Immediate	29	(2)	0	(0)
Emergent	215	(17)	0	(0)
Urgent	767	(62)	464	(36)
Less Urgent	193	(16)	703	(54)
Non-Urgent	22	(2)	123	(10)

	**ED N = 4,181**		**AUCC N = 2,875**	
	N	(%)	N	(%)
**Arrival**				
Ambulance	1,905	(46)	58	(2)
Self	2,207	(53)	2,816	(98)
**Acuity**				
Immediate	52	(1)	0	(0)
Emergent	823	(20)	0	(0)
Urgent	2708	(65)	1007	(35)
Less Urgent	531	(6)	1585	(55)
Non-Urgent	52	(1)	278	(10)

Figure. Number of patients randomized by hour of day, all clinical areas

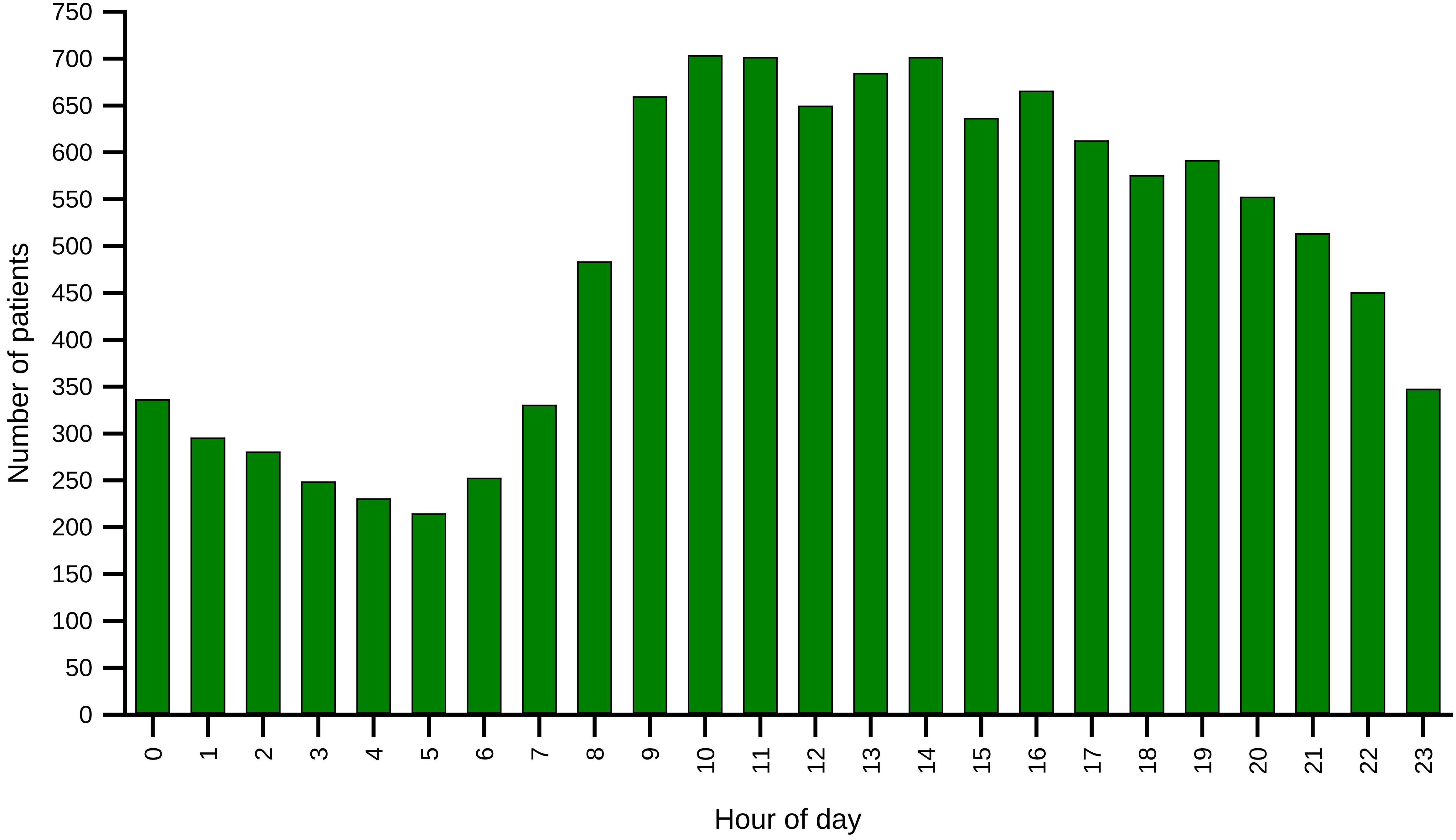

Figure. Rapid HCV antibody test results per hour of day
(based on hour that test results appear in chart), all clinical areas

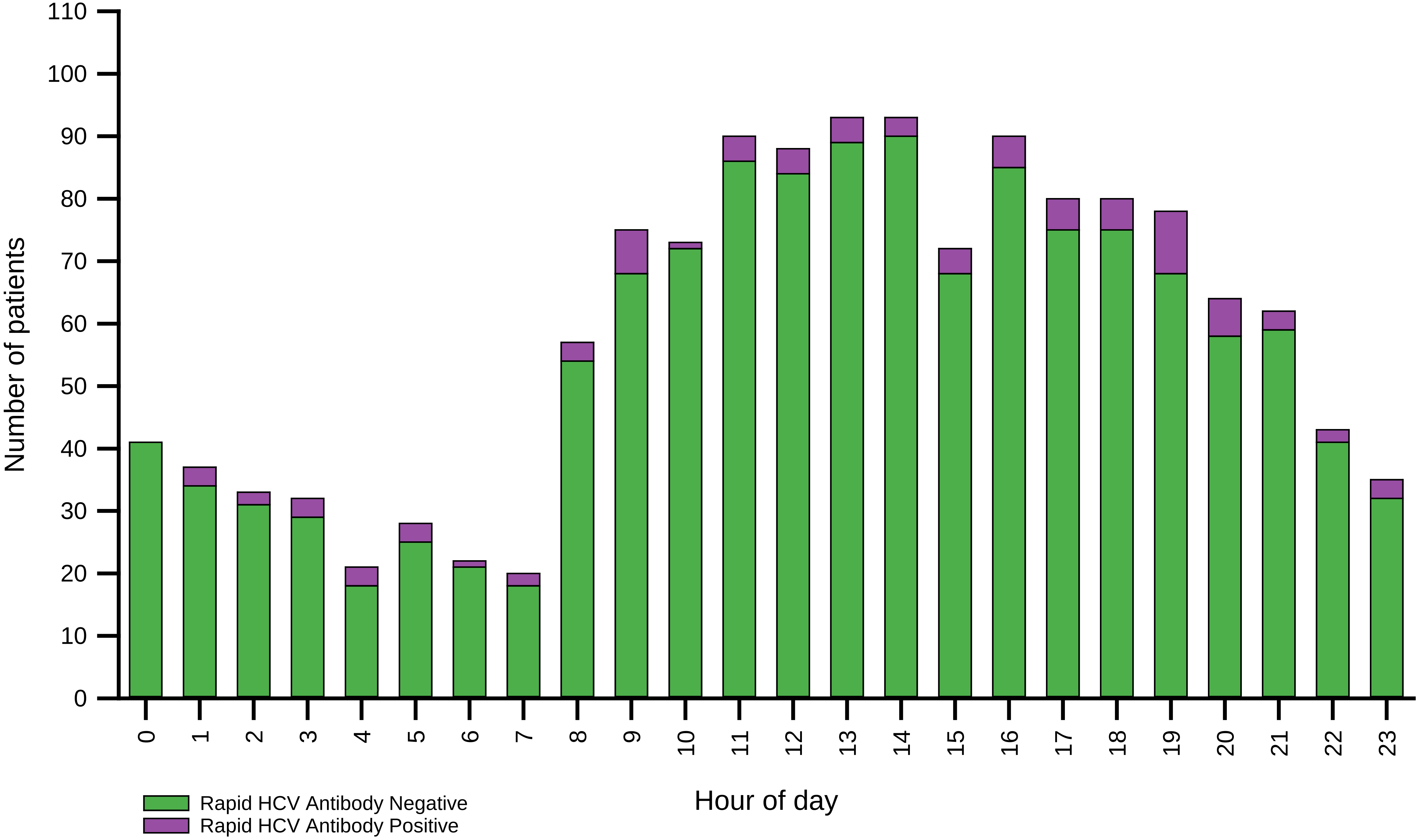

Figure. Number of "Missing" targeted questions per day, all clinical areas

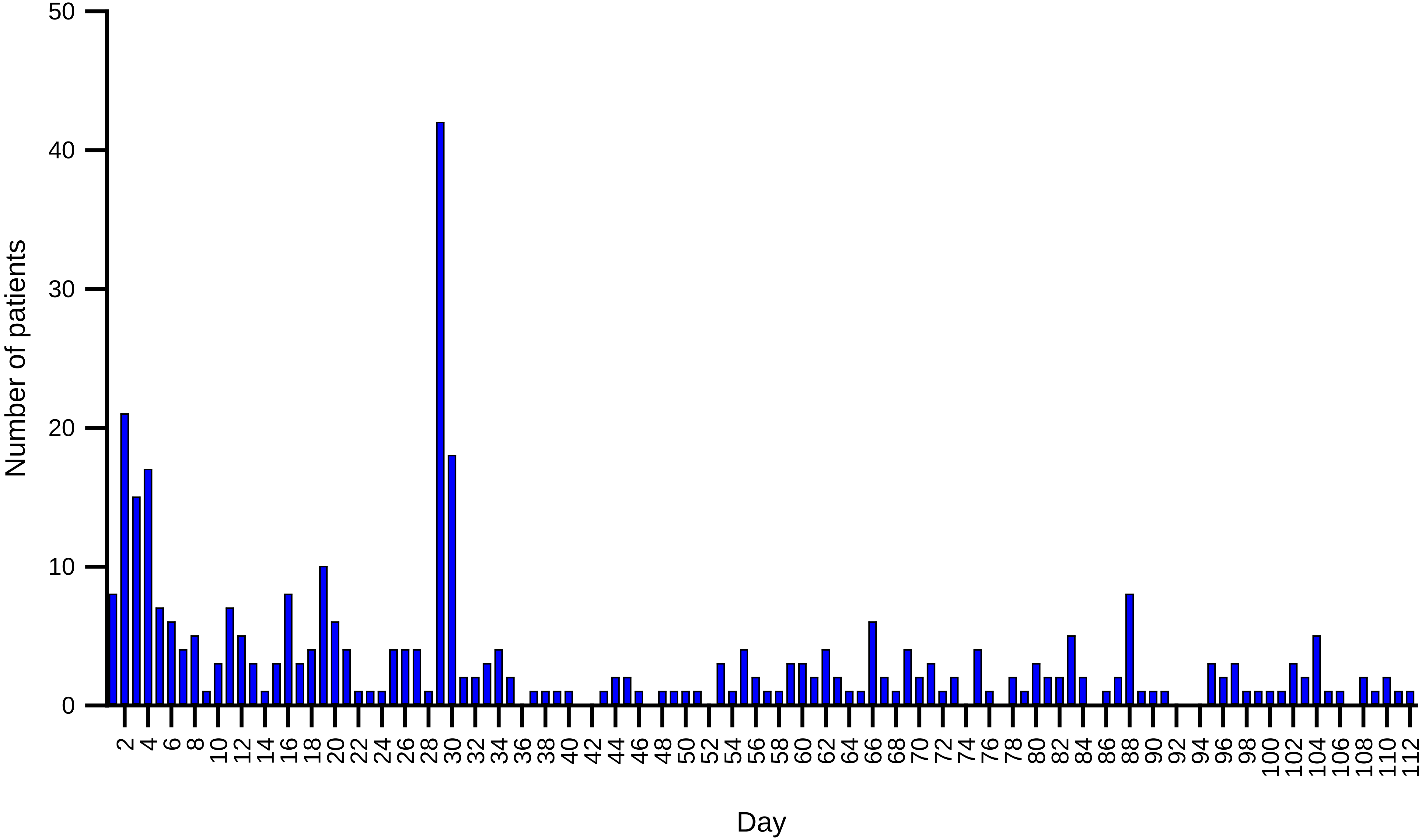

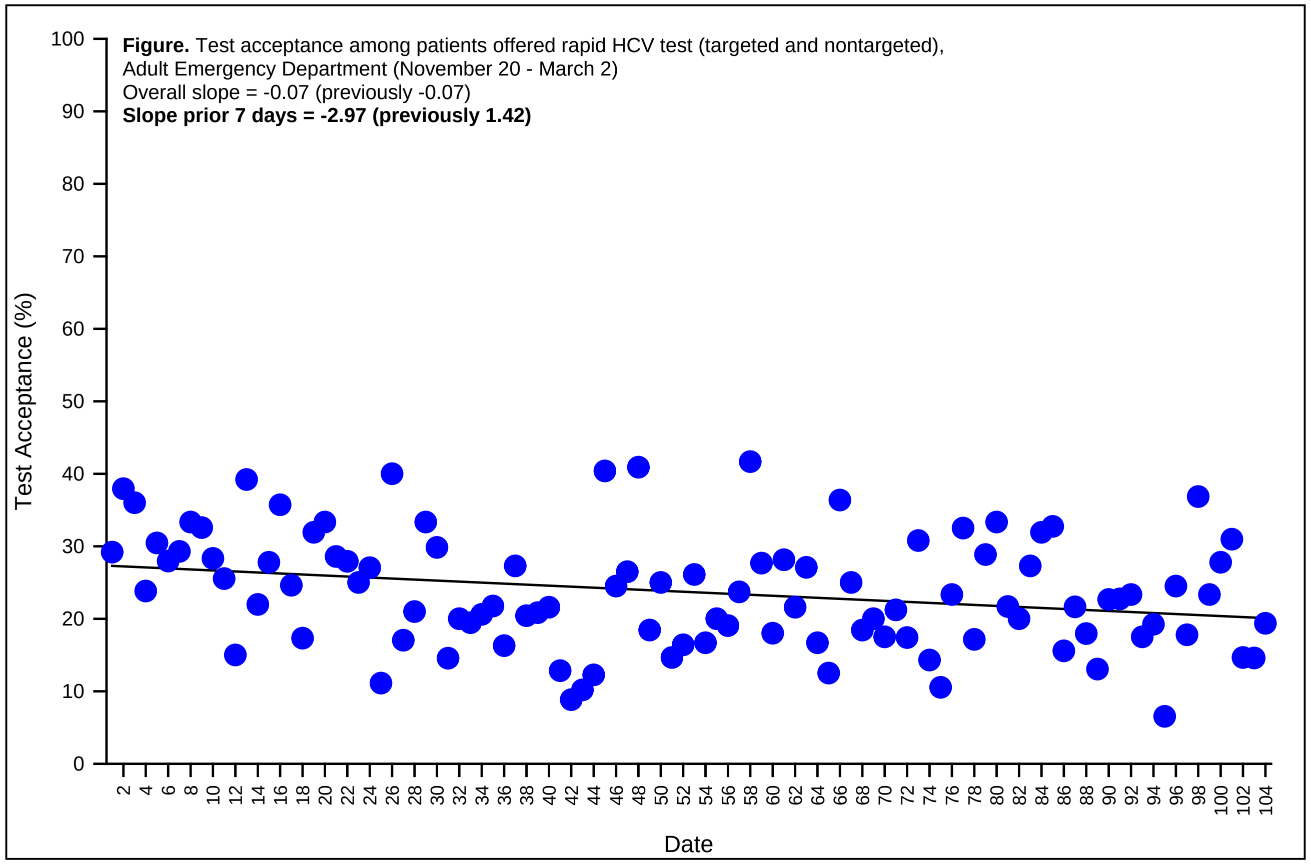
Figure. Test acceptance among patients offered rapid HCV test (targeted and nontargeted),
Adult Emergency Department (November 20 - March 2)
Overall slope = -0.07 (previously -0.07)
Slope prior 7 days = -2.97 (previously 1.42)
Test Acceptance (%)
Date

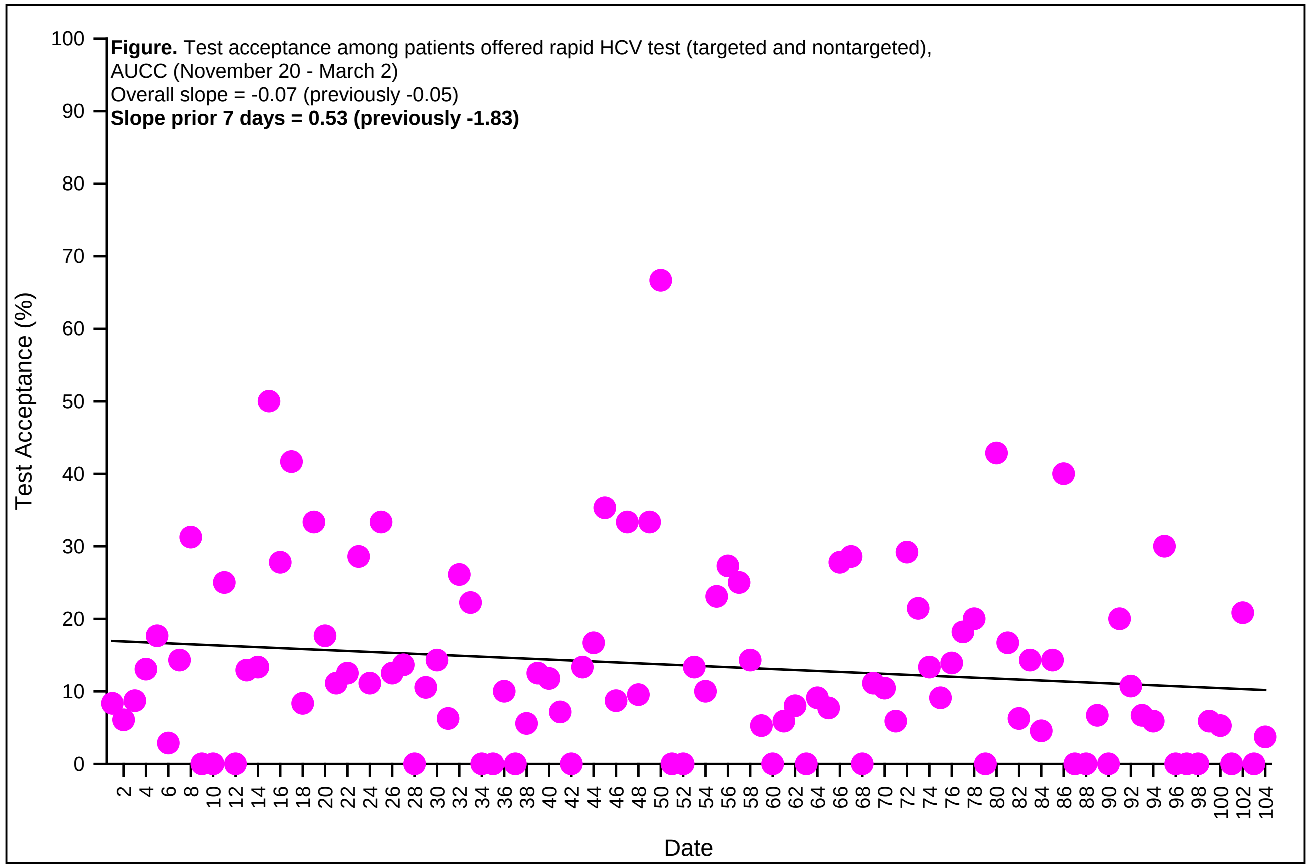
Figure. Test acceptance among patients offered rapid HCV test (targeted and nontargeted),
AUCC (November 20 - March 2)
Overall slope = -0.07 (previously -0.05)
Slope prior 7 days = 0.53 (previously -1.83)
Test Acceptance (%)
Date

Figure. Median length of stay among eligible/randomized patients and "not assigned" patients, AUCC (November 20 - January 13)

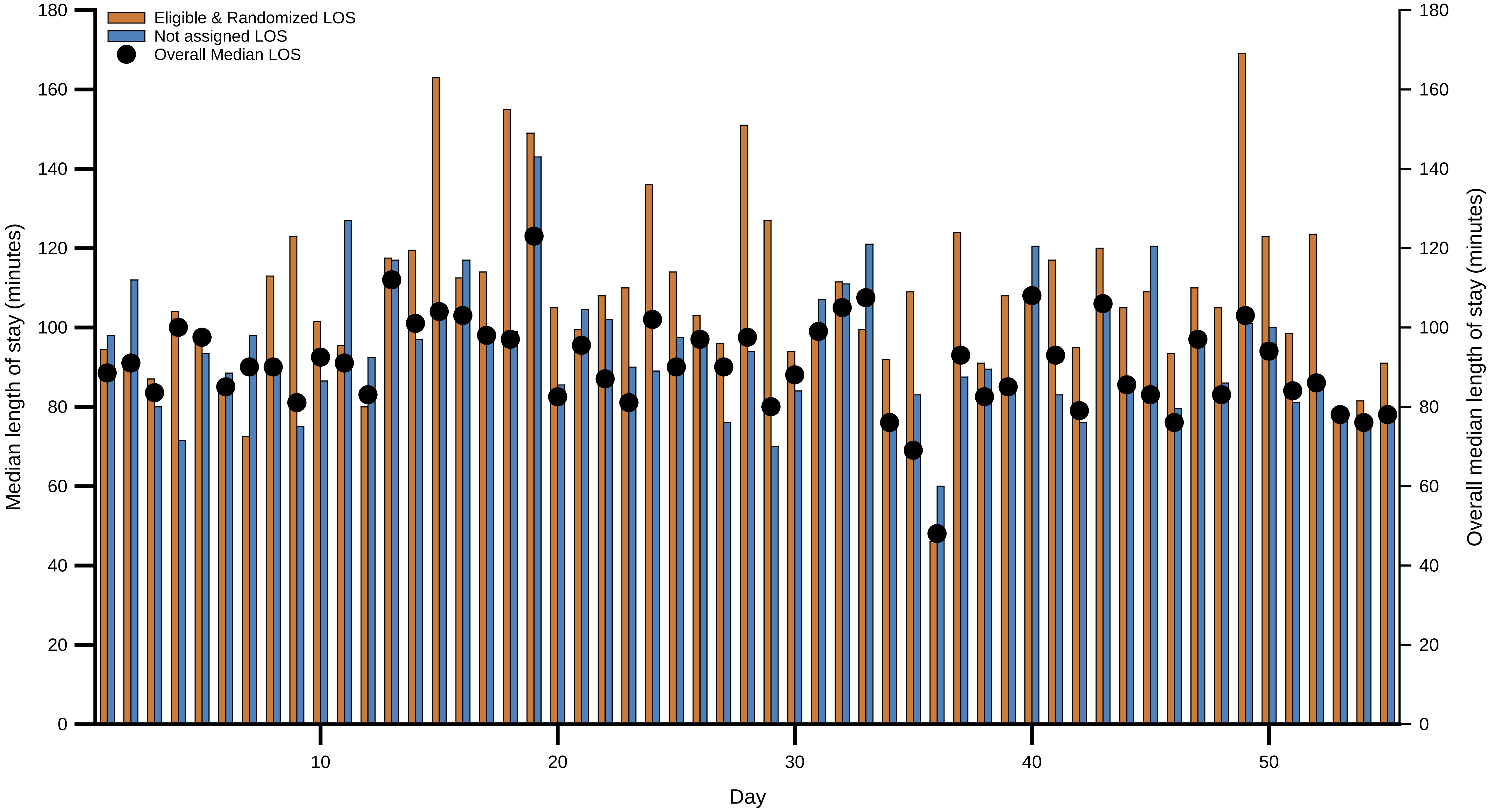

Figure. Exclusion BPA Status by individual **Adult ED** Nurses. The denominator represents the number of patients that nurse clicked “Triage Complete” and the Exclusion BPA was present for that patient. The numerator is the final status of the Exclusion BPA and the nurse that triggered/acknowledged the BPA was the same nurse that clicked “Triage Complete” AND the Exclusion BPA was triggered before “Triage Complete” was selected.

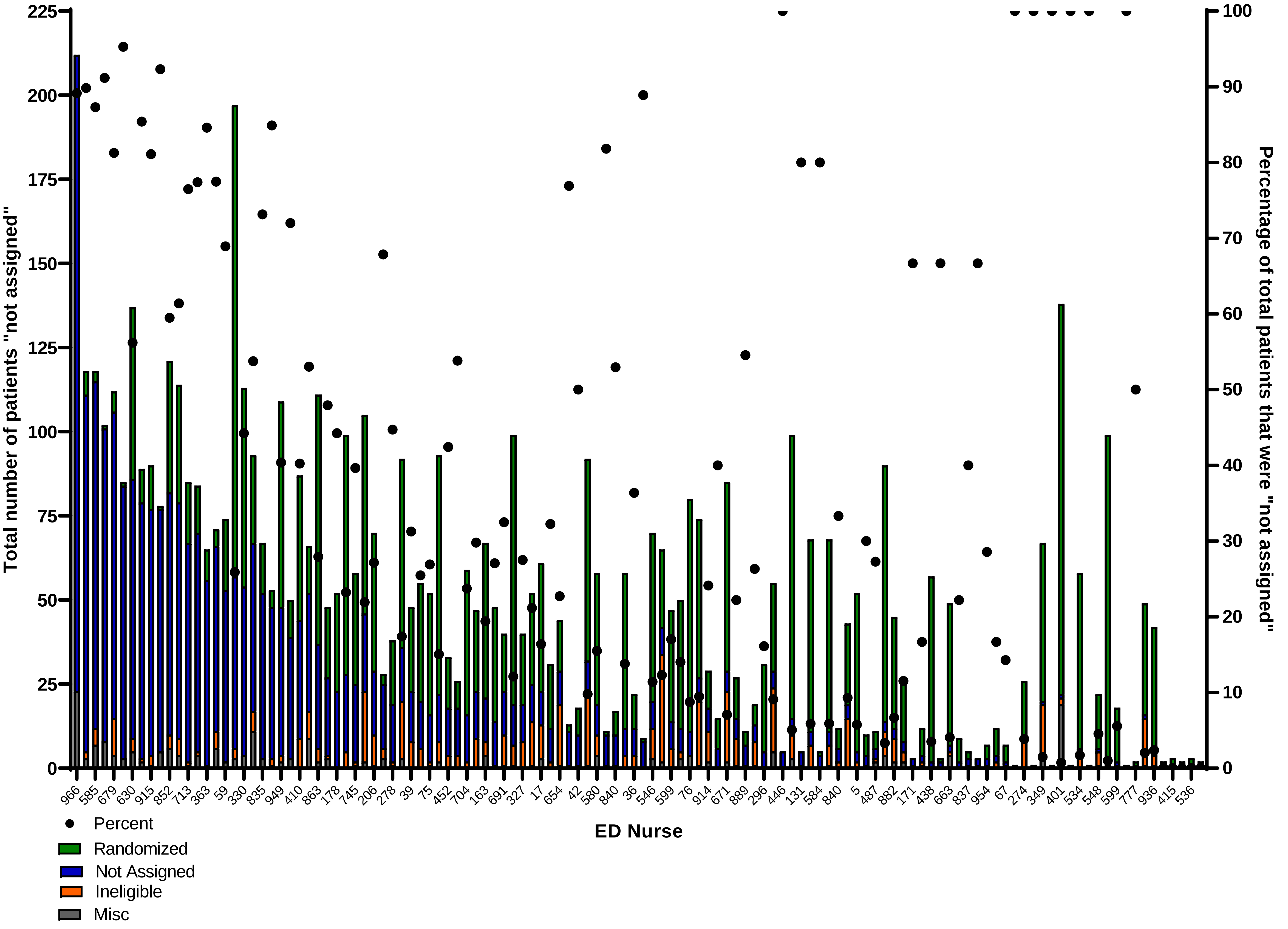

Figure. Exclusion BPA Status by individual **AUCC** Nurses. The denominator represents the number of patients that nurse clicked "Triage Complete" and the Exclusion BPA was present for that patient. The numerator is the final status of the Exclusion BPA and the nurse that triggered/acknowledged the BPA was the same nurse that clicked "Triage Complete" AND the Exclusion BPA was triggered before "Triage Complete" was selected.

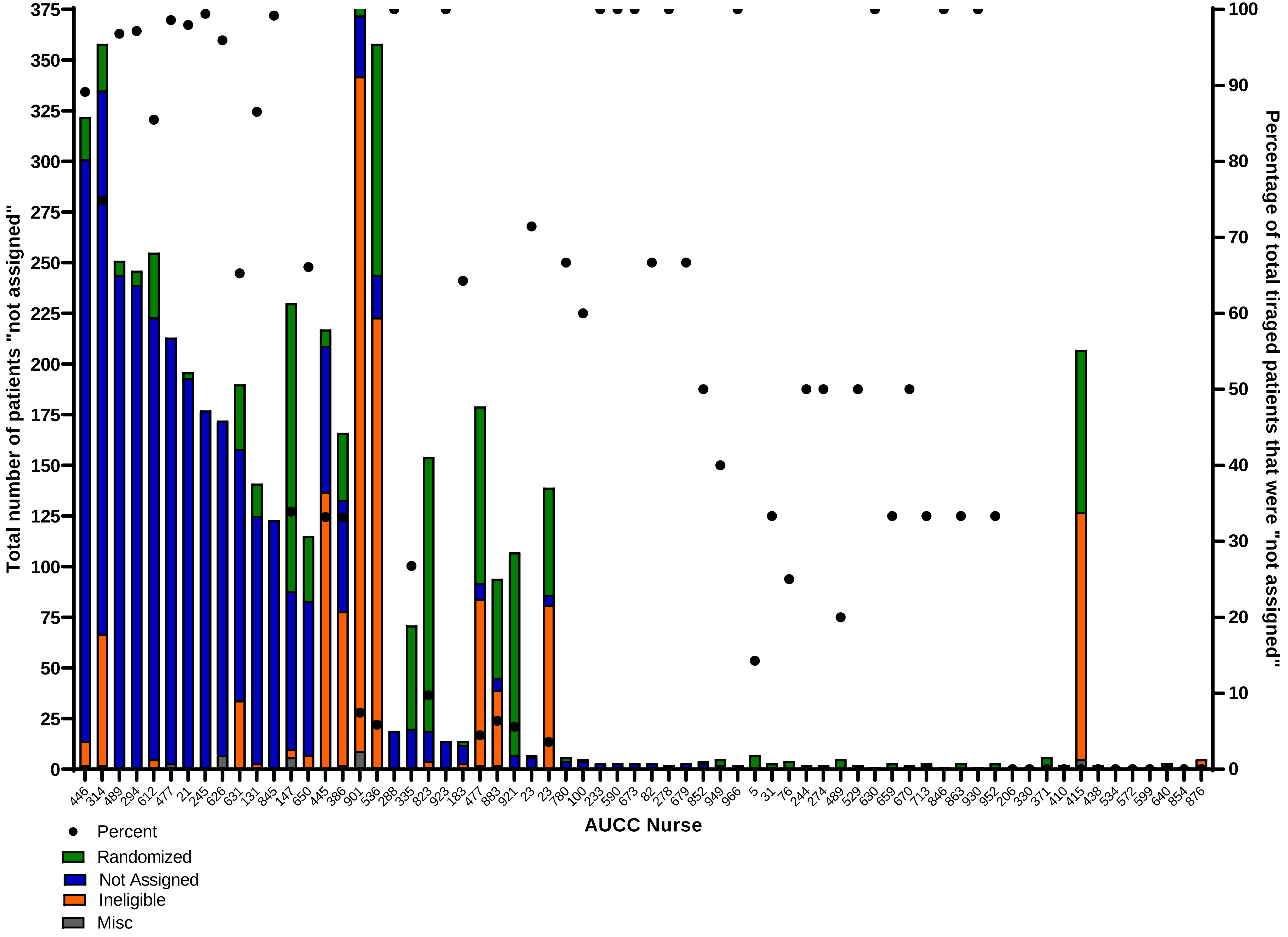

Determining Effective Testing in Emergency Departments and Care Coordination on Treatment Outcomes (DETECT) for Hepatitis C (Hep C)

STUDY PROTOCOL

PIs: Jason Haukoos, MD, MSc
Sarah Rowan, MD

NIH Project Title: The Determining Effective Testing in Emergency Departments and Care Coordination on Treatment Outcomes (DETECT) for Hepatitis C (Hep C) Trial

Grant Number: R01DA042982

Funding Organization: National Institute on Drug Abuse (NIDA) / National Institutes of Health (NIH)

ClinicalTrials.gov Number: NCT04003454 (The DETECT Hep C Screening Trial) & NCT04026867 (The DETECT Hep C Linkage-to-Care Trial)

COMIRB Number: 17-2327

PRINCIPAL INVESTIGATORS:

Jason Haukoos, MD, MSc
Department of Emergency Medicine
Denver Health Medical Center
Professor of Emergency Medicine, Epidemiology, and Clinical Sciences
University of Colorado School of Medicine
Colorado School of Public Health
University of Colorado Denver
Telephone: (303) 602-5174
Email: Jason.Haukoos@dhha.org

Sarah Rowan, MD
Division of Infectious Diseases
Denver Health Medical Center
Associate Professor of Medicine
University of Colorado School of Medicine
Telephone: (303) 602-6392
Email: Sarah.Rowan@dhha.org

PROJECT MANAGER:

Emily Caruso, MSPH
Department of Emergency Medicine
Denver Health Medical Center
University of Colorado School of Medicine
Telephone: (303) 602-5178
Email: Emily.Caruso@dhha.org

PROJECT COORDINATORS:

Kevin Kamis, MPH
Denver Public Health
Denver Health Medical Center
Telephone: (303) 602-8729
Email: Kevin.Kamis@dhha.org

Stephanie Gravitz, MPH
Department of Emergency Medicine
Denver Health Medical Center
Telephone: (303) 602-5274
Email: Stephanie.Gravitz@dhha.org

Carolynn Lyle, PA-C, MPH
Department of Emergency Medicine
Denver Health Medical Center
Telephone: (303) 602-5235
Email: Carol.Lyle@dhha.org

CO-INVESTIGATORS:

Alia Al-Tayyib, MSPH, PhD
Denver Public Health
Denver Health Medical Center
Department of Epidemiology
Colorado School of Public Health

James Galbraith, MD
Department of Emergency Medicine
University of Mississippi

Edward Gardner, MD
Denver Public Health
Denver Health Medical Center
Division of Infectious Diseases
University of Colorado School of Medicine

Yu-Hsiang Hsieh, PhD
Department of Emergency Medicine
Johns Hopkins University

Benjamin Linas, MD, MPH
Departments of Medicine and Epidemiology
Boston University

Michael Lyons, MD, MPH
Department of Emergency Medicine
University of Cincinnati College of Medicine

Jake Morgan, PhD
Department of Health Law, Policy & Management
Boston University School of Public Health

Richard Rothman, MD, PhD
Department of Emergency Medicine
Johns Hopkins University

Allison Sabel, MD, MPH, PhD
Department of Biostatistics and Informatics
Denver Health Medical Center
Colorado School of Public Health

Douglas White, MD
Department of Emergency Medicine
Highland Hospital / Alameda County Medical Center

David Wyles, MD
Division of Infectious Diseases
Denver Health Medical Center
University of Colorado School of Medicine

CONTACT INFORMATION:

Department of Emergency Medicine
Denver Health Medical Center
777 Bannock Street, Mail Code 0108
Denver, Colorado 80204 USA
Phone: (303) 602-5178
Email: Emily.Caruso@dhha.org

PROTOCOL VERSION HISTORY AND AMENDMENTS

Date	Version	Description of Modifications
October 31, 2018	1.0	Original study design as proposed in November 2016 with modifications in May 2017.
January 1,2019	2.0	Inclusion of supplemental award and minor modifications to protocol.
February 15, 2019	2.1	Added survey methodology to sample patients enrolled in Aim 1 who are allocated to nontargeted screening (Sections 6E and 6I).
March 5, 2019	2.2	Modified targeted risk questions.
August 12, 2019	2.3	Updated description of Clinical Referral.<br>Updated exclusion criteria for length of stay (<60 minutes).<br>Updated exclusion/inclusion criteria for Aim 2, and minor additions to linkage navigation referral and coordination procedures.<br>Addition of ClinicalTrials.gov numbers for Aim 1 & 2<br>Outcome language updated to match ClinicalTrials.gov<br>Addition of Certificate of Confidentiality and OHRP certificate for the inclusion of prisoner.
November 1, 2019	2.4	Minor edits.
January 9, 2020	2.5	Aim 2 enrollment expansions to include patients who self-disclose previous HCV diagnosis and opportunity to enroll patients with positive confirmatory RNA after ED discharge. Addition of prisoner RNA follow up procedures.
June 15, 2020	2.6	Addition of “Aim 4: Social determinants of HCV linkage, treatment and cure”. Addition to include JHH and UMMC in survey implementation to prospectively collect data for antibody positive patients tested through the Aim 1 workflow.
July 1, 2020	2.7	Addition of special considerations for performing enrollment of participants in the setting of the COVID-19. Also, addition of description of e-consent procedures.
December 3, 2020	2.8	Revising consent procedures for the Aim 2 Linkage Trial to request a waiver of documentation of written informed consent.
January 21, 2021	2.9	Clarification of verbal consent procedures for Aim 2 and Aim 4, including additional justification for these procedures given inequity in access to and understanding of technologies needed for eConsent.
May 15, 2021	3.0	Blinded sample size re-estimation and consolidation of Statistical Analysis Plan.
July 7, 2021	3.1	Modification to analytic plan for Aim 2 and updated Figure 2.
January 29, 2022	3.2	Clarification of sample size estimation for Aim 2.
February 17, 2022	3.3	Modification to follow-up period for longitudinal outcomes.

ABBREVIATIONS AND DEFINITIONS

AASLD – American Association for the Study of Liver Diseases
ACASI – Audio Computer Administered Self-Interview
CDC – Centers for Disease Control and Prevention
CER – Cost-Effectiveness Ratio
CFR – Code of Federal Regulations
CoC - Certificate of Confidentiality
DAA – Direct-Acting Antivirals
DCC – Data Coordination Center
DHMC – Denver Health Medical Center
ED – Emergency Department
EHR – Electronic Health Record
HCV – Hepatitis C Virus
HHS – Health and Human Services
ICER – Incremental Cost-Effectiveness Ratio
IDU – Intravenous Drug Use
IDSA – Infectious Diseases Society of America
IOM – Institute of Medicine
IQR – Interquartile Range
IRB – Institutional Review Board
OHRP – Office for Human Research Protections
PWID – Person Who Injects Drugs
RR – Risk Ratio
SD – Standard Deviation
SFTP – Secure File Transfer Protocol
SSR – Sample Size Re-Estimation
SUD – Substance Use Disorder
SVR – Sustained Virologic Response
USPSTF – United States Preventive Services Task Force

CONTENTS

1. SUMMARY 9
2. SPECIFIC AIMS and HYPOTHESES 10
3. BACKGROUND AND SIGNIFICANCE 11
4. RATIONALE 14
5. PRELIMINARY STUDIES 16
6. METHODS 18

AIM 1 – "Emergency Department Screening" 18
- A. Study Design 18
- C. Population 19
- D. Interventions 19
- E. Data Collection & Research Procedures 20
- F. Outcome Measures 20
- G. Data Management 20
- H. Analytic Plan 20
- I. Sample Size Estimation 21
- J. Sample Size Re-Estimation 22

AIM 2 – "Linkage-to-Care" 25
- A. Study Design 25
- B. Setting 25
- C. Population 25
- D. Interventions 25
- E. Data Collection & Research Procedures 27
- F. Outcome Measures 27
- G. Data Management 27
- H. Analytic Plan 28
- I. Sample Size Estimation 28

AIM 3 – "Cost Effectiveness" 29
- A. Study Design 29
- B. Setting 29
- C. Population 29
- D. Interventions 29
- E. Data Collection and Research Procedures 29
- F. Outcome Measures 30
- G. Data Management 30

H. Analytic Plan 30
I. Sample Size Estimation 31
Aim 4 – "Disparities and Social Determinants of Health" 32
A. Study Design 32
B. Setting 32
C. Population..32
D. Intervention..32
E. Data Collection & Research Procedures 32
F. Outcome Measures...33
G. Data Management & Research Procedures 33
H. Analytic Plan 33
I. Sample Size Estimation 34
7. HUMAN SUBJECTS PROTECTIONS 34
A. Institutional Review Board...34
B. Descriptions, Risks, and Justification of Procedures 35
C. Estimated Duration of the Study...42
D. Number and Distribution of Subjects 42
E. Examinations, Laboratory Tests, Procedures, and Follow-up Visits 43
F. Protected Health Information 44
G. Risks..44
H. Benefits..46
I. Limitations 46
J. Data Monitoring Plan 48
K. Summary of Knowledge to be Gained..48
8. STATISTICAL ANALYSES PLANS FOR THE CLINICAL TRIALS 49
A. Aim 1 – "Emergency Department Screening Trial" 49
B. Aim 2 – "Linkage-to-Care Trial" 49
9. REFERENCES 50

APPENDIX

1. SUMMARY

The Determining Effective Testing in Emergency Departments and Care Coordination on Treatment Outcomes (**DETECT**) for Hepatitis C (**Hep C**) Trial will compare the effectiveness of nontargeted rapid opt-out HCV screening versus targeted rapid opt-out HCV screening in multiple urban emergency departments (**EDs**) in the United States (**"Screening Trial"**). Recognizing the critical importance of linkage to care following identification, we will then evaluate the effectiveness of linkage navigation plus clinician referral versus clinician referral alone (**"Linkage Trial"**). We will then use program costs and trial results to compare screening and linkage strategies from the perspective of the institution while also projecting long-term costs and cost-effectiveness from a societal perspective using an established Monte Carlo transition-state model from the Center for Health Economics of Treatment Interventions for Substance Use Disorder, HCV, and HIV (**CHERISH**) (**"Cost Effectiveness"**).[1,2] Finally, we will specifically increase enrollment and collect gender-based data for participants in the linkage trial to evaluate gender differences in rates of linkage, the effect of linkage navigation on gender, and associations between gender and treatment outcomes, and how other disparities and social determinants of health (**SDoH**) effect HCV testing, linkage to care, and treatment (**"Disparities and Social Determinants of Health"**).

2. SPECIFIC AIMS and HYPOTHESES

AIM 1 – Emergency Department Screening: To compare the effectiveness of non-risk-based (nontargeted) and risk-based (targeted) HCV screening when integrated into high-volume, urban EDs

Hypothesis 1: Nontargeted HCV screening is significantly associated with identification of new HCV diagnoses when compared to targeted HCV screening.

AIM 2 – Linkage-to-Care: To compare the effectiveness of linkage navigation plus clinician referral versus clinician referral alone for patients with HCV identified in EDs

Hypothesis 2: Clinician referral plus linkage navigation, which includes structured counseling and formal system-based linkage, significantly increases the proportion of newly HCV-diagnosed individuals who complete HCV care visits and initiation of treatment for HCV when compared to clinician referral alone.

AIM 3 – Cost Effectiveness: To measure and compare programmatic costs and project long-term clinical outcomes, costs, and cost-effectiveness of ED-based screening for HCV and linkage to HCV care

Hypothesis 3: Nontargeted HCV screening coupled with linkage navigation will yield the best outcomes and be cost-effective per newly HCV-diagnosed patient, with an incremental cost-effectiveness ratio <$100,000/quality-adjusted life years gained.

AIM 4 – Disparities and Social Determinants of Health

Disparities Aim A: To compare gender differences in linkage to HCV care for patients HCV-diagnosed in EDs, and to compare the relative effectiveness of linkage navigation plus clinician referral versus clinician referral alone for men and women

Disparities Hypothesis A: Women will have higher rates of linkage to HCV care than men overall and the addition of linkage navigation support to clinician referral will have a smaller effect on linkage rates for women than for men.

Disparities Aim B: To identify gender-specific factors associated with linkage-to-care, treatment adherence, and cure for patients with HCV

Disparities Hypothesis B: Factors associated with HCV linkage and cure will be identified and will differ between men and women.

Social Determinants of Health Aim: To understand how social determinants of health (SDoH) effect HCV linkage-to-care, initiation, and completion of treatment among patients with HCV identified in the ED

Social Determinants of Health Hypotheses: (a) Two variables, housing stability (social) and current illicit substance use (behavioral) will be main drivers of the associations between SDoH and HCV treatment, linkage-to-care, and initiation, and will be identified as the SDoH most strongly associated with HCV treatment outcomes while other SDoH variables will show weaker associations; and **(b)** A history of depression and anxiety will modify the association between SDoH and HCV linkage and treatment such that the negative association will be exacerbated for those with a history of depression or anxiety.

3. BACKGROUND AND SIGNIFICANCE

The burden of disease from hepatitis C (**HCV**) is substantial and increasing. HCV is the most common blood borne infection in the United States.[3] As of 2016, 3.5 million individuals are estimated to be chronically infected with HCV and of these >50% remain undiagnosed.[4,5] HCV infection is often indolent and individuals living with HCV, including those who develop cirrhosis, may remain asymptomatic for years until ultimately presenting with decompensated liver disease or cancer (**Figure 1A**). At this stage, treatment is less effective, liver injury is largely irreversible, and 5-year mortality exceeds 50%.[6] Morbidity and mortality from HCV infection has increased significantly in recent years,[7-9] with approximately 16,000 deaths annually in the United States attributed to HCV infection and thousands more attributed to HCV-associated conditions.[7,10-12] The economic impact of caring for patients with undiagnosed and untreated HCV exceeds $10 billion in annual direct medical costs.[12,13] As such, HCV infection poses a major public health problem in the United States and its early identification is a critical public health priority.[14]

HCV screening is crucial to decreasing morbidity and mortality, and reducing infections. Testing for HCV infection is the first in a series of important interventions aimed at optimizing the care continuum, leading ultimately to cure of the disease (**Figure 1B**).[15,16] Well-tolerated, highly-effective direct-acting antivirals (**DAAs**) have revolutionized HCV treatment,[17-23] dramatically reducing morbidity and mortality, and halting transmission. In the 2014 – 2016 update to the Department of Health and Human Services' action plan, "Combating the Silent Epidemic of Viral Hepatitis: Action Plan for the Prevention, Care, & Treatment of Viral Hepatitis," the second key priority area includes expanding testing to "identify persons infected with viral hepatitis early in the course of their disease."[24] As high impact prevention prioritizes effectiveness and costs, feasibility of implementation, and coverage of target populations, HCV screening is the principal means of prevention of HCV-related diseases and transmission.

The Centers for Disease Control and Prevention (**CDC**) currently recommends HCV screening for individuals with the following risks: born from 1945 – 1965, history of injecting drugs, receipt of blood products or organ transplants prior to 1992, long-term hemodialysis, human immunodeficiency virus (**HIV**) infection, or those who have persistent and unexplained abnormal alanine aminotransferase levels.[25,26] In 2010, the Institute of Medicine (**IOM**) published a national strategy for the prevention of infectious hepatitis principally because prior public health efforts (specifically, risk factor-based screening) had not succeeded in controlling the epidemic.[27] The IOM concluded that at-risk individuals commonly do not know they are at risk. Subsequently, the U.S. Preventive Services Task Force (**USPSTF**) and American Association for the Study of Liver Diseases (**AASLD**)-Infectious Diseases Society of America (**IDSA**) published recommendations for HCV screening, calling for screening of those from the "birth cohort" or with high risk attributes, congruent with current CDC recommendations;[28,29] however, their recommendations also concluded

Figure 1. Natural progression of HCV infection (**A**) and the HCV care continuum (**B**).

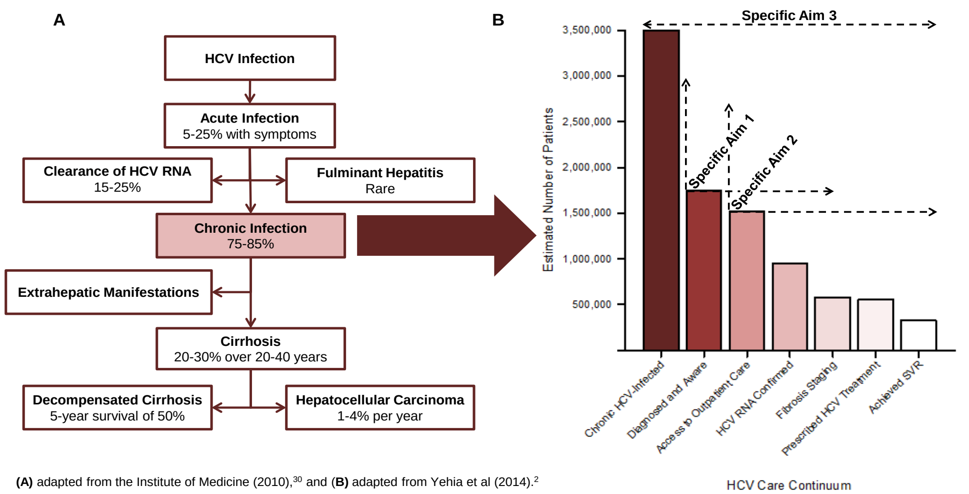

(**A**) adapted from the Institute of Medicine (2010),[30] and (**B**) adapted from Yehia et al (2014).[2]

that "…no randomized trials or observational studies have compared clinical outcomes of different approaches to screening for HCV", supporting the critical need for well-designed prospective comparative effectiveness research for HCV screening and linkage-to-care.

Emergency departments are important clinical settings for HCV screening. Primary care is also a priority clinical setting for HCV screening; programs designed to target high-risk populations in community health centers report chronic HCV prevalence of 8-9%.[16,30] However, in 2013, over 41 million individuals were uninsured, and although the provisions of the Affordable Care Act reduced this number, 27 million individuals remained uninsured in 2016, with millions more underinsured.[31] Access to primary care is particularly limited in certain geographic regions.[32] Emergency departments (**EDs**) serve as a medical safety net by, in part, filling the gap for those who do not have access to health care.[33] EDs have also been a major focus of infectious diseases screening in the United States (e.g., influenza, Ebola, HIV).[34-49] This is driven by the fact that over 135 million ED visits occur annually,[50] EDs serve substantial numbers of at-risk patients,[33] and are common sites of missed diagnostic opportunities.[51] Recognizing their potential value, in 2001 the CDC endorsed ED-based HIV screening as part of the national strategy to address HIV.[52] Since then, substantial progress has been made to improve effectiveness of ED-based HIV screening, an important contributor to the decreasing prevalence of undiagnosed infections in the United States.[53] In 2007, Rothman *et al.* published a conceptual framework of ED-based HIV testing strategies, which included diagnostic testing (i.e., testing based on clinical signs or symptoms), targeted screening (i.e., testing high-risk subpopulations), and nontargeted screening (i.e., testing all individuals regardless of risk).[39] This conceptual model lends itself perfectly to *HCV* screening in EDs. Given the burden of HCV, the successes of HIV screening programs, and the role EDs serve as a safety-nets, EDs are an ideal setting to identify patients with undiagnosed HCV and facilitate linkage-to-care.

Although targeted HCV screening is currently recommended by the CDC and USPSTF, narrow screening strategies (e.g., those only targeting the "birth cohort" or persons who inject drugs [**PWID**]) may miss up to 25% of patients living with HCV.[28] Other arguments against targeted screening suggest that clinicians are too busy to perform risk assessments and patients are unwilling or unable to provide risk information.[54] Based on similar concerns, in 2006 the CDC dramatically shifted its *HIV* screening paradigm to recommend nontargeted opt-out HIV screening in all healthcare settings.[55] Since 2006, 18 studies have evaluated *nontargeted* HIV screening in ED settings.[44] All demonstrated the ability to identify patients living with HIV, including a larger or comparable number of newly diagnosed cases when compared to diagnostic testing or targeted screening, while contributing substantially to the growth of ED-based HIV screening programs nationally.[56-58] Estimates of HCV prevalence in EDs are at least 10-fold higher than HIV prevalence, supporting the notion that nontargeted HCV screening may outperform targeted HCV screening.[59-61] Nontargeted HCV screening has not been evaluated in a way to inform whether this method would increase testing uptake by de-stigmatizing the process, or decrease uptake by eliminating risk factor discussions and lowering individuals' motivations to test. Because of these potential differences in uptake and uncertainty of unrecognized risk, the yield of nontargeted HCV testing is unknown.

After diagnosis, linkage-to-care is a critical component of the HCV Care Cascade. Effective linkage-to-care has been a major challenge for ED-based HCV testing with studies reporting linkage for only 20-39% of those newly diagnosed.[59,60,62] Unlike HCV, many HIV testing programs have successfully implemented active linkage programs, yielding linkage well over 80% within 3 months of HIV diagnosis.[63-65] Linkage navigators have been utilized in community-based HCV testing programs, but while the role of navigation has been described in these settings, the added benefit of a linkage navigator above and beyond standard clinician referral has not been evaluated in a prospective, comparative manner.[66-68] Additionally, the effectiveness of navigators for PWID, a traditionally under-engaged and under-treated population, has not been assessed. Given that HCV is now curable with short courses of well-tolerated regimens, and the increasing availability of these breakthrough

medicines, the critical element to slowing the epidemic is HCV testing and effective linkage to care. In high-volume ED settings, a linkage navigator may improve laboratory follow-up, posttest counseling, linkage-to-care, and ultimately HCV treatment and cure.

Women have been underrepresented in all aspects of HCV research. Of the reported cases of chronic HCV, a larger proportion occur in men than in women (65% vs 35% in Colorado), while reported acute HCV cases have a significantly smaller gender differences (54% vs 46% in Colorado).[69,70] Given the larger proportion of chronic cases occurring in men, clinical trials have included significantly more men than women.[17-22,71-73] Sub-analyses have not detected gender differences in the efficacy of treatment, but may have been underpowered to detect gender differences in side effects, completion rates, or other endpoints. Studies involving populations more likely to be affected by *acute* HCV, such as PWID, have been similarly skewed toward male participants, despite evidence suggesting that there are nearly as many women affected by acute HCV as men. For example, in a study of DAA treatment among PWID in primary care settings in Australia, only 32% of participants were women.[74] In Vancouver, a study of HCV knowledge and treatment willingness among PWID had a similarly disproportionate percentage of women (33%).[75] The few studies that specifically involve gender in HCV care focus on pregnancy-related issues.[76] This is important considering the 4-7% risk of vertical transmission, though given that DAAs are not approved for use in pregnancy, a similar focus on engagement in HCV care for growing number of women of childbearing age who are living with HCV but not pregnant would benefit those individuals and their risk sharing partners, and would have the added benefit of eliminating the risk of vertical transmission if the woman does become pregnant.[77,78] One study of knowledge about HCV among pregnant women with opiate use disorders (50% of whom were HCV-positive) revealed extensive HCV knowledge gaps, underscoring the need to develop better targeted HCV prevention and treatment programs for women, particularly those of childbearing age.[79]

Understanding the influence of gender on human health is a national priority. By studying gender differences in engagement in HCV care among individuals who are diagnosed with HCV in an ED setting, we will work toward understanding key factors associated with successful linkage to HCV care and completion of HCV treatment. This knowledge will lead to a better understanding of barriers and facilitators to HCV care and how they differ for women and men, allowing for development of targeted interventions. This works is in alignment with the NIH Strategic Plan for Women's Health Research in the following ways: (1) this study will consider the roles of gender on the health outcomes of women and men living with HCV; (2) we will evaluate the impacts of a wide variety of factors that affect women's abilities to engage in healthcare; and (3) data from this study will inform the development of personalized interventions that support HCV treatment for women.

4. RATIONALE

Aim 1 – "Emergency Department Screening"

To evaluate the clinical effectiveness and to maximize internal validity of rapid opt-out HCV screening in high-volume urban EDs, we will use a randomized controlled design to compare nontargeted to targeted HCV screening.[80] We chose to perform a 2-arm trial because (1) to date, no head-to-head comparative trial has been performed, (2) the CDC, USPSTF, and AASLD-IDSA recommend performing targeted screening, and (3) preliminary research supports the hypothesis that nontargeted screening may be superior. We will perform this trial across three institutions in the United States to incorporate different geographic regions, reflecting different HCV-affected populations and barriers to care. We devised a "pragmatic" framework, including fully integrated screening, testing, and linkage-to-care processes, to maximize generalizability. Scientific and organizational structures are modeled after The HIV TESTED Trial.

Aim 2 – "Linkage-to-Care"

We will perform a distinct randomized clinical trial of clinician referral plus linkage navigation versus clinician referral alone leveraging patients identified with HCV from the trial performed in Aim 1 (**Figure 2**). A focus of this second trial will be on individuals most at risk for transmitting HCV (i.e., <40 years of age[63,64] and PWID[81]). We have planned this trial around this subgroup, and given the smaller sample size, will use stratified block randomization to maximize balance, ensure sufficient numbers of patients, and maximize analytic efficiency.[82] As is the case with many EDs in the United States, the EDs participating in The DETECT Hep C Trial have a variety of protocols and experiences with linkage to HCV care. Thus, we chose 1 site for enrollment in this trial to minimize the heterogeneity of the linkage process and to ensure that sites with more developed linkage protocols do not compromise care for patients enrolled in the clinician referral only arm. Although the ED at Denver Health has extensive experience with *HIV* screening, HCV screening has not yet been initiated; thus it will provide a naïve screening and linkage environment in which to evaluate *de novo* HCV linkage services. This will ensure that all patients receive the standard of care for new HCV diagnoses and will allow evaluation of linkage navigation in addition to clinician referral while minimizing contamination from existing linkage processes. Distinct from the Screening Trial, the Linkage Trial will also include individuals with untreated hepatitis C, verified by the electronic medical record.

Aim 3 – "Cost Effectiveness"

Multiple studies concur that HCV therapy is cost-effective[83-87] and that routine screening for HCV is cost-effective in specific high prevalence environments (i.e., substance abuse treatment centers and methadone maintenance programs).[87] To date, however, no studies have rigorously evaluated routine ED-based HCV screening. The results of Aims 1 and 2 will demonstrate the effectiveness of screening and linkage. Aim 3 will investigate the economic consequences of each strategy from both institutional and societal perspectives, and provide a comparative cost evaluation, while projecting long-term cost effectiveness and outcomes. The economic perspective and estimate of the value of ED-based HCV screening are critical in an environment characterized by limited resources directed to HCV care. We will thus employ the Hepatitis C Cost-Effectiveness (**HEP-CE**) model, a Monte Carlo transition-state simulation model of HCV screening, linkage, treatment, and disease progression to simulate the lifetime progression of a cohort of hypothetical individuals seen in the ED, assuming both HCV screening and linkage strategies.

Aim 4 – "Disparities and Social Determinants of Health"

Given that linkage rates from EDs have traditionally been very low, <30%,[62] it is critical to determine the factors associated with linkage to HCV care so they can be addressed at the individual and system levels. Research in linkage to *HIV* care suggests that barriers to linkage are generally related to healthcare system factors like wait times for provider appointments, social factors like stigma, and population-specific characteristics such as homelessness or substance abuse.[88] However, little is known about barriers to linkage to care for patients with HCV, or how the barriers differ by gender. This supplemental award, however, will allow additional enrollment in this linkage-to-care trial to optimize our ability to evaluate gender differences in rates of linkage and associations between gender, linkage barriers, and treatment outcomes.

Further, high-risk communities, such as PWID, face barriers including social, behavioral, and structural factors that influence their likelihood of successfully linking and initiating treatment for HCV. Understanding these factors may inform individual care, healthcare systems, and policy, as well as the development of public health interventions to increase HCV treatment linkage and initiation among the US's high-risk populations. Recognizing the importance of how social and structural factors impact an individual's likelihood to initiate HCV treatment is critical to slowing transmission and disease progression. As such, we will assess the associations between SDoH and successful HCV treatment initiation among people who test positive for HCV in the ED. We also will examine if self-reported depression or anxiety modifies the association between SDoH and HCV treatment linkage and initiation, as mental health has been shown to act as a modifying co-morbidity for individuals receiving treatment for a chronic disease.

5. PRELIMINARY STUDIES

Our research team has pioneered investigations in ED-based HIV screening since 2004 and, more recently, HCV screening.[56,57,59-61,89-101] The overarching goal of our work is to evaluate infectious diseases screening among high-risk and underserved populations in EDs, and to combine meticulous scientific rigor, robust program evaluation, and implementation science to best inform processes and policies on a national level. The following preliminary research was performed by members of our team.

1) HCV Screening in an Integrated Healthcare System: Missed Opportunities: The goals of this study were to estimate: (1) prevalence of HCV infection in an integrated healthcare system (Denver Health); (2) prevalence of undocumented HCV infection stratified by age; and (3) the proportion of individuals who had at least 1 ED visit prior to HCV diagnosis. From 2008 through 2013, 24,863 HCV antibody tests were performed, of which 3,893 (16%) were antibody positive. Of these, 2,217 (57%) had RNA testing performed, and 1,851 (83%) were RNA positive. Of the 1,851 individuals, 73% were from the birth cohort and the majority was uninsured. From January 1, 2014 through September 30, 2015, an additional 476 individuals were identified with chronic HCV infection. Using structured medical record abstraction and blinded research assistants, we determined that 30% (145) of those individuals had previously undocumented HCV infection, and of those, 61% (89) had at least 1 ED visit in the 3 years prior to their diagnoses, and 49% (71) were not from the birth cohort. We concluded that the burden of HCV infection is substantial in this urban, integrated, safety-net healthcare system, prevalence of undiagnosed infection is high, and a large proportion of HCV-infected but undiagnosed patients receive care in EDs, suggesting a missed opportunity for diagnosis.[102]

2) HCV in EDs – Significant Prevalence, Unrecognized Disease, and Feasibility of Nontargeted Strategies: We have published important preliminary findings in *Hepatology, Annals of Emergency Medicine,* and *Clinical Infectious Diseases*.[59,60,98,99] These studies were conducted independently and non-comparatively, but with the following goals: (1) to evaluate the performance of targeted and nontargeted HCV screening in EDs and estimate prevalence of chronic HCV infection; (2) to estimate seroprevalence of HCV infection among ED patients; and (3) to identify proportions of individuals among nontargeted and blinded seroprevalence populations who would be missed if only targeted strategies were used (**Table 1**). We concluded: (1) a high prevalence of unrecognized chronic HCV infection exists among ED patients targeted for HCV testing; (2) linkage to HCV care from EDs is challenging; (3) while birth cohort screening would identify nearly twice as

Table 1. Summary of preliminary research for HCV screening, HCV prevalence, and risk in multiple EDs in the United States.

Panel A. Targeted HCV Screening	Year	Offered N	Tested N	Ab+ %	RNA+ %	Linked %
Galbraith *et al.*	2013	2,325	1,534	11%	8%	38%
White *et al.*	2014	7,554	2,568	10%	7%	24%
**Panel B. Nontargeted HCV Screening**	**Year**		**Tested N**	**Ab+ %**	**RNA+ %**	**Linked %**
Galbraith *et al.*	2015-16	-	6,205	7%	4%	-
White *et al.*	2015	-	2,432	7%	5%	36%
**Panel C. Blinded HCV Seroprevalence**	**Year**	**Tested N**	**Ab+ %**	**RNA+ %**	**Previously Undiagnosed* %**	**Non-BC + Non-PWID***
Hsieh *et al.*	2013	4,713	14%	-	**31%**	**25%**
Lyons *et al.*	2008-09	924	14%	11%	**66%**	**14%**

Abbrev: Ab+ = antibody positive; RNA+ = ribonucleic acid positive; BC = birth cohort; PWID = person who injects drugs; “-” = unknown, not reported, or not collected. *Percentages of Ab+ (Hsieh *et al.*) or RNA+ (Lyons *et al.*)

many cases as risk-based screening alone, employment of both strategies would still miss up to 25% of HCV infections, suggesting that in high-volume ED settings nontargeted screening may be more effective; and (4) the ED is an important clinical setting for high-impact HCV screening and linkage-to-care interventions.

3) Successful Performance of a Large-Scale HIV Screening Trial in Multiple EDs: The HIV TESTED Trial: This study (R01AI106057; clinicaltrials.gov ID: NCT01781949), recently completed enrollment ahead of schedule and within budget.[103] Its goal was to evaluate the effectiveness of 3 rapid HIV screening strategies (nontargeted HIV screening, enhanced targeted HIV screening using the Denver HIV Risk Score[104,105] and traditional targeted HIV screening).[52] Using methods proposed in this application, including use of 4 distinct electronic health systems (**EHS**) systems from 4 institutions (Denver Health Medical Center, Denver, CO; Highland Hospital, Oakland, CA; Johns Hopkins Hospital, Baltimore, MD; University of Cincinnati Medical Center, Cincinnati, OH), we developed and successfully integrated patient-level randomization algorithms and targeted screening methods into routine emergency care. For patients randomized to 1 of the 2 targeted screening arms, nurses asked risk questions and entered patient responses into EHS-embedded instruments that guided screening and test offering. During the study period, 76,235 patients were randomized and 14,294 completed HIV testing in a balanced manner across all 4 sites. This study represents the largest, most comprehensive multi-center, prospective pragmatic randomized trial of HIV screening in EDs. The results will greatly improve our understanding of how to provide effective rapid HIV testing in this important clinical setting.

6. METHODS

AIM 1 – "Emergency Department Screening"

A. Study Design

We will perform a prospective pragmatic randomized effectiveness trial[106-108] that will allow us to directly compare 2 HCV screening methods while minimizing threats to internal validity.[109] Patients will be screened for HCV infection using 1 of 2 interventions using a balanced patient-level random allocation scheme built into existing EHSs for each ED, using methods previously described (**Section 5 – Preliminary Studies**). Patients will therefore be offered HCV testing based on the result of the screening arm to which they are assigned, and in the case of the targeted arm, the results of the risk assessment evaluation performed by the intake nurse. All randomization will be completely integrated into electronic medical screening systems and workflow at each site as was the case with The HIV TESTED Trial. Integration of randomization into the electronic systems will allow for real-time concealed random allocation. Nurses who perform screening and all other ED staff (e.g., physicians, technicians) will understand the conceptual goals of the project but will be blinded to study hypotheses, and patients will be completely blinded to the purpose of the study (**Figure 2**).

Figure 2. Study schematic for The DETECT Hep C Trials. [R = randomization, S = survey/stratification, = primary outcome, = secondary outcomes]
Abbreviations: PWID = person who injects drugs

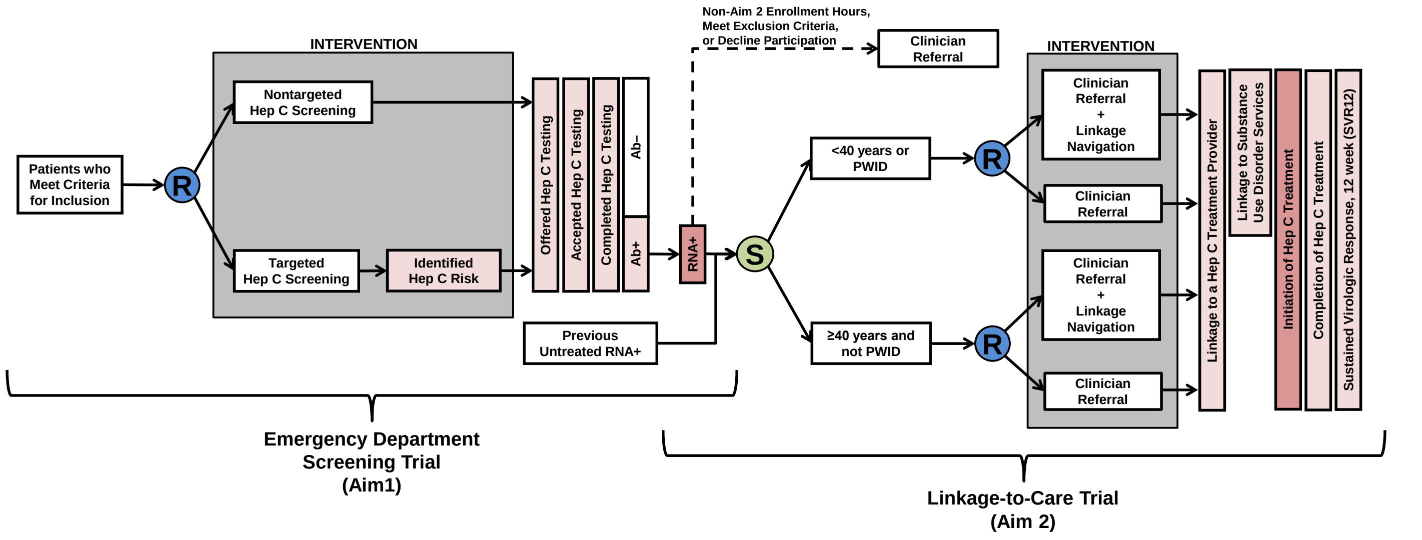

B. Setting

This study will be performed at multiple sites, including the EDs at Denver Health Medical Center (**DHMC**) (Denver, CO), Johns Hopkins Hospital (**JHH**) (Baltimore, MD), and the University of Mississippi Medical Center (**UMMC**) (Jackson, MS) (**Table 2**). These sites were selected because of the heterogeneity of populations served, local HCV epidemics, geographic distributions, expertise of investigators, and likelihood of successful completion of the trial. DHMC will serve as the coordinating site for this project. To enhance and streamline the Institutional Review Board (**IRB**) process, the Colorado Multiple IRB (**COMIRB**) will serve as the central IRB with UMMC and JHH ceding to COMIRB.

Table 2. Characteristics of study sites for Specific Aim 1.

Site	Setting	Hospital Type	Annual ED Census	Racial/Ethnic Minorities* (%)	Uninsured Patients† (%)	Birth Cohort (%)	PWID (%)
Denver Health MC	Urban	L1/C/SN/T	96,000	53%	17%	30%	7%
Johns Hopkins Hospital	Urban	L1/U/T	69,000	77%	15%	31%	6%
University of Mississippi MC	Urban	L1/U/T	66,000	73%	36%	25%	§

Abbreviations: MC = Medical Center; ED = emergency department; L1 = level 1 trauma center; C = county; SN = safety-net; U = University; T = teaching/academic. *Defined as Asian, Black, Hispanic, American or Alaskan Native, Native Hawaiian, or Non-Hawaiian Pacific Islander. †Defined as uninsured or sponsored by a state healthcare discount program. Does not include Medicaid or Medicare. §Unknown, although 12% HCV antibody prevalence among whites born since 1965.

C. Population

Consecutive patients ≥18 years of age who present to the EDs during the study enrollment period will be eligible for inclusion if they are considered clinically stable by screening nurses or physicians and capable of providing consent for medical care. Due to the integrated nature of the study, patients will be enrolled 24 hours per day, 7 days per week. Patients will be excluded if they: (1) are <18 years of age; (2) are unable to consent for care (e.g., altered mentation, critical illness or injury); (3) have already participated in the trial; (4) self-identify as already living with HCV; or (5) have an anticipated ED length of stay <60 minutes. See **Section 7 – Human Subjects Protection** for details related to human subjects protection and consent.

D. Interventions

Intervention: Nontargeted Screening

The nontargeted HCV screening arm will consist of implementing non-risk-based rapid opt-out HCV screening. Consecutive patients who present to the ED for evaluation, who meet criteria for inclusion, and who are randomized to this arm will be offered, regardless of risk, voluntary free rapid HCV testing by nurses using a standardized script and opt-out consent during medical screening.

Intervention: Targeted Screening

The targeted HCV screening arm will consist of implementation of risk-based rapid opt-out HCV screening using current recommendations for HCV screening by the CDC, USPSTF, and AASLD-IDSA.[25,26,28,29] Targeted HCV screening will consist of offering HCV testing to those identified with the following specific risk characteristics, adapted from the above recommendations: born between 1945–1965 (i.e., "birth cohort"); injection drug use (**IDU**); intranasal drug use; tattoo or piercing in an unregulated setting; or blood transfusion or organ recipient before 1992. Risk factor surveys will be incorporated into electronic medical screening and patient tracking systems in each ED. As such, nurses will use this tool while electronically entering responses to each of the risk questions during screening. Nurses will apply the set of risk questions to all patients who meet criteria for inclusion and are randomized to the targeted screening arm. Those who have an affirmative response to any question will be considered at increased risk for HCV infection and offered rapid HCV testing using opt-out consent (**Appendix, Targeted Screening Risk Assessment Tool**). Patients who deny all risk will be considered low risk and not offered rapid HCV testing.

Positive HCV Antibody Results Follow-up

For patients who test positive for HCV antibodies in either arm, the clinical team will explain the process for obtaining RNA results and will provide the Clinician Referral Intervention as outlined below under Aim 2.

E. Data Collection & Research Procedures

We will collect the following data for all eligible patients: (1) patient ED visit information (unique patient identifier, acuity level, mode of arrival, date/time of the visit); (2) demographics (age, sex, race, ethnicity, primary language); (3) payer information (commercial, Medicare, Medicaid, self, or state-sponsored); (4) details of randomization, including the intervention assigned and results of risk screening, if applicable; (5) whether a patient was offered, accepted, and completed rapid HCV testing; (6) results from all rapid HCV tests, and for all patients with a positive HCV antibody test, results of HCV RNA testing; (7) whether patients with positive HCV RNA tests were successfully linked into care; and (8) components of the HCV Care Cascade. Data from (1) through (6) will be collected prospectively using methods developed and validated by our team from each institution's electronic screening, patient tracking, and laboratory reporting systems. We have long-standing and extensive experience interfacing with such systems across different institutions, obtaining large amounts of valid patient-level data. Data from (7) and (8) will be retrospectively obtained using trained personnel and structured procedures, and using an 18-month time frame (**Appendix, Longitudinal Outcomes & HCV Care Continuum Data Collection Instrument**)[110].

Risk Assessment Survey: We will conduct surveys of a representative sample of patients enrolled in this aim who are allocated to the nontargeted arm. A research assistant will collect risk information, as collected when allocated to the targeted arm, using a closed-response data collection instrument (**Appendix, Risk Assessment Survey**).

F. Outcome Measures

The primary outcome will be confirmed cases of newly diagnosed chronic HCV, defined as patients who test positive for HCV antibody and HCV RNA and without a prior HCV diagnosis. Secondary outcomes will include *all* patients identified with chronic HCV in anticipation of testing patients with previously diagnosed HCV infection (i.e., those who do not identify as having been previously diagnosed and who are re-diagnosed during this trial), as well as HCV test offer, acceptance, and completion, and progression through the HCV Care Continuum (i.e., receipt of RNA results, HCV genotype result, fibrosis staging, evaluation by an HCV treatment expert, treatment with DAAs, and sustained virologic response at 12 weeks after treatment completion [**SVR12**] [i.e., HCV cure]).

G. Data Management

Denver Health will serve as the Data Coordinating Center (**DCC**) and data from non-Denver institutions will be transferred to the DCC using a Secure File Transfer Protocol (**SFTP**). Data will be extracted from each institution's EHS and cleaned so that variables are consistent across sites. The cleaned and de-identified dataset will be sent via SFTP to the DCC for final data concatenation, cleaning, and analyses, performed using the most current version of SAS (SAS Institute, Inc., Cary, NC).

H. Analytic Plan

All statistical analyses will be conducted by the study's statistical core, which includes the study biostatistician and principal investigators. After cleaning and locking the dataset, the primary analysis will be performed while

blinded to allocation. Although no formal interim analyses are planned for Aim 1, the study team may perform preliminary analyses for purposes of presentation at scientific meetings; these instances, if they occur, will be explicitly qualified as such and described as preliminary.

Figure 3. Power simulations of test offer (Panel A), acceptance (Panel B), completion (Panel C), and prevalence (Panel D). Each point from each panel represents 1,000 Monte Carlo simulated trials of total randomized patients (thousands) with all other assumptions held constant. ◯ represent anticipated assumptions.

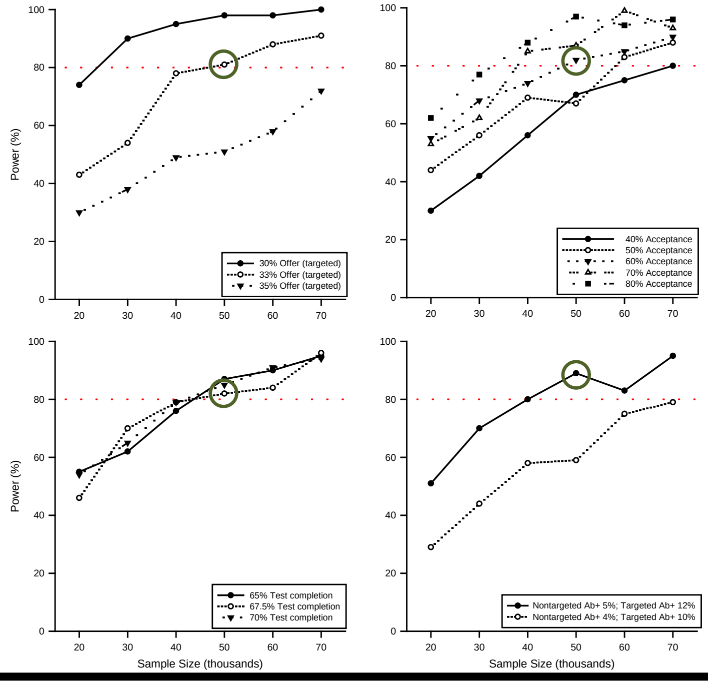

Continuous data will be reported as medians with interquartile ranges (**IQRs**) and categorical data as proportions or percentages with 95% confidence intervals (**CIs**). Bivariate statistical tests (e.g., Wilcoxon rank sum test, Fisher's exact test, etc.) will be used to compare variables between study groups. Patient-level data will be reported for all variables given that patients can only enroll in the trial once. The primary unit of analysis will be patient visits. All analyses will be performed using the intention-to-treat principle and no interim analyses are planned given the pragmatic trial approach and minimal risk to subjects.[111] Given the randomized design, the primary comparison will include an unadjusted risk ratio (**RR**) for newly-identified HCV case (primary outcome) with 95% CIs, specifically comparing nontargeted HCV screening to targeted HCV screening (primary hypothesis), but using a random effect hierarchical model to account for institution-level clustering, if needed.[111,112] Statistical significance for the primary analysis will be defined as $p<0.05$ based on two-tailed statistical testing, which includes a lower 95% confidence limit of the RR >1.0. Sensitivity analyses will be performed to account for patients who were identified as antibody positive but RNA negative, and subsequently determined to be previously treated for hepatitis C. Secondary comparisons will include all other outcomes by study arm, stratified by age, gender, race, ethnicity, income, and education level.

See **Statistical Analysis Plan** for a consolidated description of all planned analyses.

I. Sample Size Estimation

The primary superiority hypothesis of this aim is that nontargeted HCV screening will be significantly associated with new chronic HCV diagnoses when compared to targeted HCV screening. As described in **Section H – Analytic Plan**, the primary statistical measure will include an unadjusted RR. Based on our *a priori* assumptions related to the performance of each screening method (i.e., 100% and 33% test offer for patients randomized to the nontargeted and targeted arms, respectively; 60% test acceptance for patients in both arms; 70% test completion for patients in both arms; 5% and 10% HCV antibody positive proportions for patients randomized to the nontargeted and targeted arms, respectively; and 65% HCV RNA positive for HCV antibody positive patients in both arms), we estimated required sample sizes by performing 1,000 simulated trials using Monte Carlo methods in SAS. Invoking the assumptions described above and their approximate ranges, we estimate requiring a minimum of 50,000 randomized patients across all sites to achieve >80% power (**Figure 3**). This will result in an estimated 13,965 completed rapid HCV tests and an estimated 611 confirmed newly diagnosed

HCV cases, resulting in an estimated unadjusted RR = 1.25 (95% CI: 1.07 - 1.48) ($\alpha = 0.05$). Accounting for the hierarchical nature of the multi-site study, and using preliminary data from The HIV TESTED Trial, we estimate the intra-cluster correlation coefficient (**ICC**) to range conservatively between 0.005 - 0.01, resulting in a Design Effect of 1.01 - 1.03, or a required 1% - 3% inflation of the sample size.[112] Given the small number of clusters (3 sites) and the relatively large number of patients enrolled at each site, we anticipate the effect of clustering to be negligible; as such, we did not specifically modify the sample size but will account for clustering in the primary analysis. Enrollment will be equally balanced across sites, resulting in a minimum of 16,667 randomized patients per site.

Risk Assessment Survey: We anticipate including 450 patients in this part of the study to provide approximate 5% 95% confidence limits (10% 95% CI) for all prevalence estimates. We anticipate balancing enrollment in this part of the study to include 150 patients per site.

J. Sample Size Re-Estimation

In early April 2021, the Screening Trial crossed the 50% enrollment target and with the final site (University of Mississippi Medical Center) having recently initiated enrollment. In general surveillance and trial monitoring, several assumptions used for the original sample size estimation were identified as being different from what was being observed during actual trial performance (**Table A**). As such, a sample size re-estimation (**SSR**), blinded to outcomes by study arm, was undertaken using Monte Carlo simulation in SAS.

Table A. Original assumptions used to estimate sample size, and weighted and inverse probability weighted estimates from observed trial performance.

	Nontargeted			Targeted		
	Original Assumptions	Weighted Estimates	IPW Estimates	Original Assumptions	Weighted Estimates	IPW Estimates
	%	%	%	%	%	%
**Test Offer**	100	93.8	82.6	33	36.1	33.0
**Test Accept**	60	26.4	33.0	60	34.0	47.0
**Test Complete**	70	62.9	56.9	70	69.3	65.2

		Aggregate Weighted Estimates	Aggregate IPW Estimates	
	%	%	%	%
**Ab+**	5	5.9	4.7	10
**RNA+**	65	42.4	31.1	65

Abbreviations: IPW = inverse probability weighted.

As of April 27, 2021 the Screening Trial was at 58.1% enrolled and using these updated enrollment data, we project randomizing 129,663 visits across the three sites to complete 13,965 HCV antibody tests as originally estimated. This increase in number of visits randomized reflectis a 2.6 increase over the number originally estimated to be randomized (i.e. 50,000). Moreover, RNA+ prevalence is signficantly lower than originally estimated (i.e. actual: 42% vs original estimate: 65%). Using both weighted and inverse probability weighted estimates to account for individual site contributions to the total trial for test offer, test acceptance, and test completion by study arm, while also using a two-way data table (**Table B**) to model potential combinations of Ab+ prevalences by study arm using aggregate Ab+

Table B. Two-way data table of potential combinations of nontargeted (NT) and targeted (T) Ab+ prevalences resulting in estimated aggregate Ab+ prevalences. Light gray represents likely potential combinations based on current weighted prevalences across sites. Dark gray represents likely potential combinations based on current inverse probability weighted prevalences.

		T Ab+ Prevalence											
		**1%**	**2%**	**3%**	**4%**	**5%**	**6%**	**7%**	**8%**	**9%**	**10%**	**11%**	**12%**
**NT Ab+ Prevalence**	**1%**	1	1.3	1.7	2.0	2.3	2.7	3.0	3.3	3.6	4.0	4.3	4.6
	**2%**	1.7	2.0	2.3	2.7	3.0	3.3	3.7	4.0	4.3	4.6	5.0	5.3
	**3%**	2.3	2.7	3.0	3.3	3.7	4.0	4.3	4.7	5.0	5.3	5.6	6.0
	**4%**	3.0	3.3	3.7	4.0	4.3	4.7	5.0	5.3	5.7	6.0	6.3	6.6
	**5%**	3.7	4.0	4.3	4.7	5.0	5.3	5.7	6.0	6.3	6.7	7.0	7.3
	**6%**	4.3	4.7	5.0	5.3	5.7	6.0	6.3	6.7	7.0	7.3	7.7	8.0
	**7%**	5.0	5.3	5.7	6.0	6.3	6.7	7.0	7.3	7.7	8.0	8.3	8.7
	**8%**	5.7	6.0	6.3	6.7	7.0	7.3	7.7	8.0	8.3	8.7	9.0	9.3
	**9%**	6.4	6.7	7.0	7.3	7.7	8.0	8.3	8.7	9.0	9.3	9.7	10.0
	**10%**	7.0	7.4	7.7	8.0	8.3	8.7	9.0	9.3	9.7	10.0	10.3	10.7
	**11%**	7.7	8.0	8.4	8.7	9.0	9.3	9.7	10.0	10.3	10.7	11.0	11.3
	**12%**	8.4	8.7	9.0	9.4	9.7	10.0	10.3	10.7	11.0	11.3	11.7	12.0

Abbreviations: NT = nontargeted; T = targeted.

prevalence, we performed a series of simulations (**Tables C-D**) to estimate the power to test the primary study hypothesis – nontargeted hepatitis C screening is superior to targeted hepatitis C screening.

Simulations using original assumptions and the originally planned 50,000 randomized visits, which the trial has already exceeded, resulted in significantly fewer total tests performed (n=5,969) and power of only 0.3% (weighted and inverse probability weighted). Using the projected 129,663 randomized visits, estimates from current trial enrollment (**Table A**), and our original hypothesized effect estimate (RR = 1.25), simulations resulted in trials with totals of 15,452-15,623 (weighted) or 16,602-16,616 (inverse probability weighted) HCV antibody tests performed (exceeding our original estimation), while achieving powers of 65.7% (weighted) and 42.9% (inverse probability weighted). Fixing the RR at 1.25, simulations of 10% increased sample sizes (N=142,629) increased powers to 70.7% (weighted) and 43.0% (inverse probability weighted), simulations of 30% increased sample sizes (N=168,562) increased powers to 73.9% (weighted) and 52.8% (inverse probability weighted), and simulation of 50% increased sample sizes (N=194,495) increased powers to 79.8% (weighted) and 57.7% (inverse probability weighted).

Table C. Simulated results to estimate sample size and power using summary estimates weighted by contributions to enrollment from each site from the trial at 58.6% of target enrollment. Each simulation represents 1,000 simulated trials using Monte Carlo methods.

Simulation	Nontargeted Ab+ Prevalence (%)	Targeted Ab+ Prevalence (%)	Δ (%)	Aggregate Ab+ Prevalence (%)	Randomized Visits	Median RR	Median NT Tests	Median T Tests	Median Total Tests	Power* (%)
Original	5	10	-5	6.7	50,000	0.91	3,839	2,130	5,969	0.3
1	9	1	+8	6.4	129,663	16.40	9,977	5,475	15,452	*100*
2	8	1	+7	5.7	129,663					
3	9	2	+7	6.7	129,663					
4	8	2	+6	6.0	129,663	7.35	10,091	5,511	15,602	*100*
5	7	2	+5	5.3	129,663					
6	8	3	+5	6.3	129,663					
7	7	3	+4	5.7	129,663	4.27	10,101	5,518	15,619	*100*
8	8	4	+4	6.7	129,663					
9	7	4	+3	6.0	129,663					
10	6	4	+2	5.3	129,663	2.73	10,100	5,514	15,614	*100*
11	7	5	+2	6.3	129,663					
12	6	5	+1	5.7	129,663					
13	7	6	+1	6.7	129,663	2.13	10,100	5,509	15,609	*100*
14	6	6	0	6.0	129,663	1.82	9,981	5,472	15,453	*100*
15	5	6	-1	5.3	129,663	1.53	10,101	5,515	15,616	*98.1*
16	6	7	-1	6.3	129,663	1.57	10,098	5,514	15,612	*99.5*
16a	5.3	7.2	-1.9	5.9	129,663	1.44	10,094	5,520	15,614	*82.3*
17	5	7	-2	5.7	129,663	1.31	10,099	5,517	15,616	71.0
18	6	8	-2	6.7	129,663	1.37	10,105	5,517	15,622	*91.4*
18a	5.5	8.0	-1.5	6.3	*194,495*	1.26	15,153	8,270	23,423	*79.8*
18b	5.5	8.0	-1.5	6.3	*168,562*	1.26	13,128	7,165	20,293	73.9
18c	5.5	8.0	-1.5	6.3	*142,623*	1.26	11,107	6,067	17,174	70.7
18d	5.5	8.0	-1.5	6.3	129,663	1.26	10,105	5,512	15,617	65.7
19	5	8	-3	6.0	129,663	1.14	10,091	5,512	15,603	26.6
20	4	8	-4	5.3	129,663	0.92	10,100	5,516	15,616	0.8
21	5	9	-4	6.3	129,663	1.02	10,100	5,517	15,617	4.0
22	4	9	-5	5.7	129,663	0.81	9,985	5,478	15,463	0
23	5	10	-5	6.7	129,663	0.92	10,104	5,519	15,623	0.1
24	4	10	-6	6.0	129,663					
25	3	10	-7	5.3	129,663					
26	4	11	-7	6.3	129,663					
27	3	11	-8	5.6	129,663					
28	4	12	-8	6.6	129,663					
29	3	12	-9	6.0	129,663					
30	2	12	-10	5.3	129,663	0.30	9,986	5,476	15,462	0

Abbreviations: RR = risk ratio; NT = nontargeted; T = targeted
*Percentage of simulated trials where the lower 95% confidence limit of the RR was >1 (indicating statistical significance) when calculating the association between nontargeted hepatitis C screening and new diagnoses when compared to targeted hepatitis C screening.

Table D. Simulated results to estimate sample size and power using summary estimates inverse probability weighted by contributions to enrollment from each site from the trial at 58.6% of target enrollment. Each simulation represents 1,000 simulated trials using Monte Carlo methods.

Simulation	Nontargeted Ab+ Prevalence (%)	Targeted Ab+ Prevalence (%)	Δ (%)	Aggregate Ab+ Prevalence (%)	Randomized Visits	Median RR	Median NT Tests	Median T Tests	Median Total Tests	Power* (%)
Original	5	10	-5	6.7	50,000	0.91	3,839	2,130	5,969	0.3
1	6	2	+4	4.7	129,663	4.55	10,057	6,556	16,613	*100*
2	5	4	+1	4.7	129,663					
3	4.7	4.7	0	4.7	129,663	1.52	10,054	6,556	16,610	*91.4*
3a	4.7	4.7	0	4.7	*108,917*	1.54	8,450	5,505	13,955	*84.0*
4	4.6	4.8	-0.2	4.7	129,663	1.47	10,052	6,555	16,607	*85.1*
4a	4.6	4.8	-0.2	4.7	*108,917*	1.48	8,443	5,505	13,948	78.3
5	4.6	4.9	-0.3	4.7	129,663	1.44	10,061	6,552	16,613	79.2
6	4.5	5.2	-0.7	4.7	129,663	1.33	10,050	6,552	16,602	60.0
7	4.4	5.3	-0.9	4.7	*194,495*	1.25	15,053	8,523	23,606	
8	4.4	5.4	-1	4.7	*168,562*	1.25	13,074	7,213	20,287	57.7
8a	4.4	5.4	-1	4.7	*142,629*	1.25	10,060	9,830	19,890	52.8
8b	4.4	5.4	-1	4.7	129,663	1.25	11,059	6,558	17,617	43.0
9	4.3	5.5	-1.2	4.7	129,663	1.20	10,059	6,559	16,618	29.2
10	4	6	-2	4.7	129,663	1.02	10,055	6,561	16,616	2.9
11	3	8	-5	4.7	129,663					
12	2	10	-8	4.6	129,663					
13	1	12	-11	4.6	129,663	0.13	10,056	6,549	15,605	0

Abbreviations: RR = risk ratio; NT = nontargeted; T = targeted
*Percentage of simulated trials where the lower 95% confidence limit of the RR was >1 (indicating statistical significance) when calculating the association between nontargeted hepatitis C screening and new diagnoses when compared to targeted hepatitis C screening.

Simulations of trials with 129,663 randomized visits with varying Ab+ prevalences by study arm, constrained by the aggregate Ab+ prevalences (5.9%, weighted; 4.7% inverse probability weighted) and

their respective 95% lower confidence limites (LCLs) (5.1% and 3.3%, respectively), resulted in RRs of 1.35 (weighted) or 1.45 (inverse probability weighted) to achieve a power >80% (**Figures A-B**). Given the steep slope of the power curves between -4 and 0 (weighted) and -2 and 0 (inverse probability weighted) for Ab+ prevalence difference, small differences in Ab+ prevalence between study arms would result in large differences in power, and many of the simulations resulted in sufficient power. As such, no changes to overall target enrollment were made.

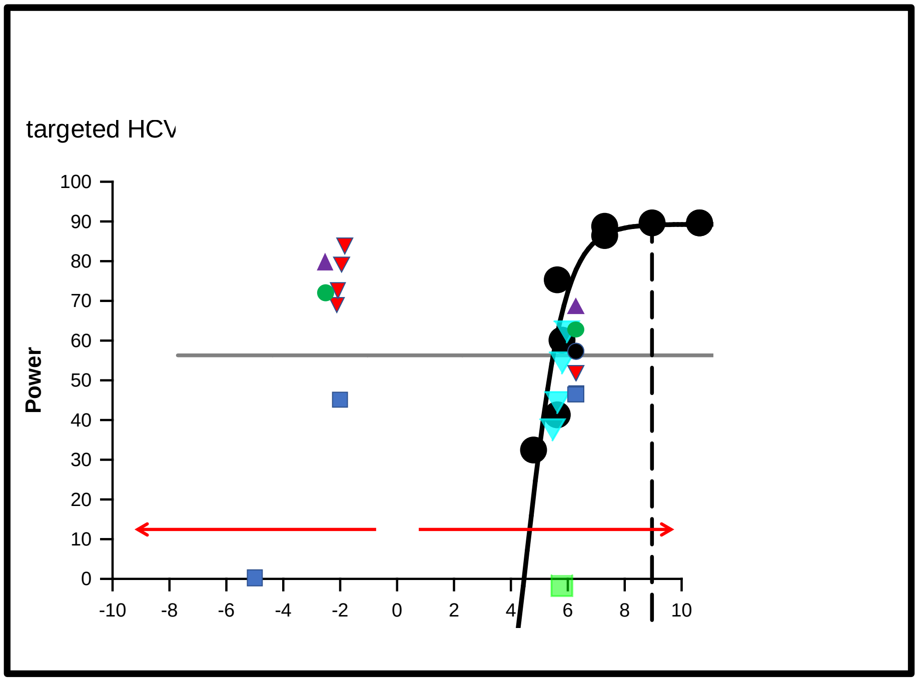

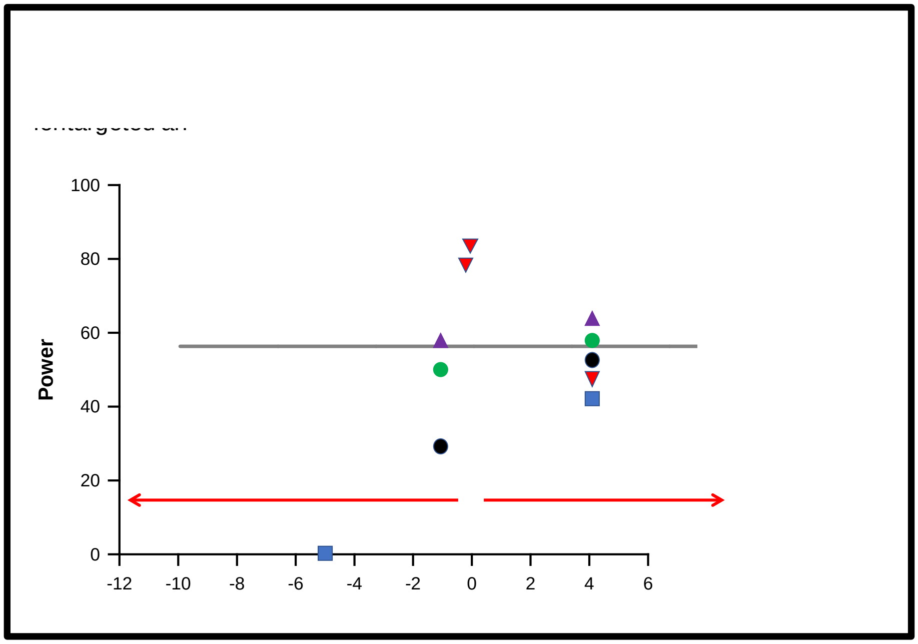

AIM 2 – "Linkage-to-Care"

A. Study Design

We will perform a prospective randomized effectiveness trial to compare 2 linkage-to-care strategies. Permuted block randomization with 2 strata (i.e., <40 years of age or active IDU [defined as IDU within 30 days], or ≥40 years of age without active IDU) and varying block sizes will be used to minimize imbalance, ensure appropriate numbers of patients in subgroups, and allow for efficient analyses. Allocation will be concealed by blinding block sizes and using the REDCap Randomization Module, a web-based platform to assign patients to arms (REDCap, Vanderbilt University, TN).[113] Although patients will not be blinded to the interventions, they will be blinded to the outcomes. Also, a trained research assistant will perform all enrollment, including stratification and randomization (**Figure 2**). Clinical staff will not be blinded, however, to assignment.

B. Setting

This study will be performed at Denver Health, a nationally recognized safety-net hospital and integrated health care system in Denver, Colorado that includes an acute care hospital and level I trauma center (DHMC), 9 federally qualified community health centers, specialty clinics including hepatology and infectious diseases, and Denver Public Health.[114] Patients will be enrolled in the ED with subsequent referral to a HCV treatment provider.

C. Population

Patients eligible for inclusion in the linkage trial will include: (1) those who meet criteria for inclusion in the screening trial (≥18 years of age, considered clinically stable by screening nurses or physicians, and capable of providing consent for medical care and without prior participation, identified or disclosed HCV infection, or an anticipated ED length of stay < 60 minutes) and who test positive for HCV antibodies will be eligible for inclusion in the linkage trial; and (2) those with untreated active HCV confirmed by the electronic medical record and who were not identified in the screening trial. Exclusions include: prisoners and individuals who live outside of Colorado (given follow-up constraints), non-Spanish or English (as the self-interview for participants will only be available in Spanish and English), and those who are pregnant (because HCV treatment is not currently approved for pregnancy). Patients whose confirmatory test results return positive for RNA after ED discharge will receive a follow up phone call from research staff to disclose positive result and be offered the opportunity to return to the Denver Health Emergency Department to participate in the linkage trial. A dedicated research assistant will screen, consent, verify contact information, provide incentives, stratify, and randomize all patients for this study during predetermined random blocks of time, including nights and weekends, to ensure representative sampling of patients in an ED, while also maximizing enrollment. See **Section 7 – Human Subjects** Protection for details related to human subjects protections and consent.

D. Interventions

Individuals who test positive for HCV in Aim 1, or who are identified as having untreated HCV through a study-specific notification built into the medical record, and who agree to participate in this trial will be randomized to one of two interventions (**Figure 2**).

Intervention: Clinician Referral

The Clinician Referral arm will serve as an "active control" and baseline standard of care. All individuals who test positive for HCV antibodies will be informed of their result and physicians will be prompted to include standardized language in the Discharge Instructions/After Visit Summary, including post-testing information and specific follow-up instructions (**Appendix – HCV Preliminary Positive Discharge [AVS] Information**). Patients will also be instructed to access their electronic patient portal (MyChart) for their RNA test results or to call a designated results line. Patients with untreated active HCV (previous detectable HCV RNA with no subsequent undetectable HCV RNA in their medical record) will receive a tailored AVS specific for individuals identified as having previously diagnosed HCV.

The research assistant will review all RNA test results. Patients who test negative for HCV RNA will not receive any follow-up contacts by the clinical or research teams. The research assistant will call patients (a maximum of three attempts) with positive RNA results to instruct the patient to follow-up with their primary care provider or a HCV treatment provider and will provide contact information for Denver Health HCV care providers.

Intervention: Clinician Referral + Linkage Navigation

The Linkage Navigation arm will consist of an additional service layered onto clinician referral and will incorporate protocols from Antiretroviral Treatment and Access Studies (**ARTAS**), the most influential studies of HIV linkage-to-care to date.[115,116] Individuals randomized to this intervention will be contacted by a linkage navigator either during the ED visit (if during business hours) or the following business day (if during non-business hours) as is the protocol for our current HIV linkage to care program. If the navigator does not contact the patient at the time of diagnosis, he or she will offer to meet with the patient in person or over the phone. For all individuals in this arm, a structured linkage navigation process will include (a) reiteration of posttest counseling messages and (b) assessment of the patient's needs for medical insurance and substance use disorder treatment.

For individuals who test positive for HCV RNA or present to the ED with untreated, active HCV, navigation will also include (c) referral to an appropriate HCV treatment provider; (d) coordination of appointments with an enrollment specialist, if needed; (e) provision of some social services (e.g., transportation vouchers); (f) referral to resources and services as needed including but not limited to health insurance enrollment assistance, HIV care, local harm reduction services (e.g. syringe access programs), housing resources, and mental health services; (g) coordination of appointment scheduling including rescheduling missed appointments through the entire hepatitis C treatment process; and (h) contacting patients after appointments to assess their understanding and any additional needs regarding engagement in HCV care. The linkage navigator will undergo structured training prior to the start of the trial, and will provide follow-up services for up to 12 months (365 days) from the time of ED testing with at least 5 attempted contacts for difficult to reach patients.

For individuals who test negative for HCV RNA, the linkage navigator will make no more than three attempts to contact the patient to (i) deliver and explain the HCV RNA results; (j) discuss the risk of reinfection and future testing recommendations; and (j) provide resources for insurance enrollment and substance use disorder treatment, as needed.

See **The DETECT Hep C Linkage Trial Study Manual** for details related to this intervention.

E. Data Collection & Research Procedures

During this aim, an assessment of barriers and facilitators to linkage to HCV care will be conducted through use of an audio computer administered self-interview (**ACASI**) offered to all enrolled patients after informed consent has been obtained but before randomization. ACASI has been shown to be a superior method for collecting potentially sensitive information in an efficient and anonymous format.[117,118] The survey will be offered in English and Spanish and will collect the following data for all enrolled patients: (1) ED visit information (unique study identifier, date/time of the visit); (2) demographics (age, sex, race/ethnicity, primary language); (3) payer information (commercial, Medicare, Medicaid, self); (4) detailed contact information;[119] (5) details of randomization; (6) clinician referral information; (7) HCV RNA testing; (8) linkage navigation details; and (9) all outcome measures. Data from (1) through (6) will be collected prospectively by a trained research assistant during enrollment, data from (7) will be collected retrospectively by a trained research assistant, data from (8) will be collected prospectively by the linkage navigator, and data from (9) will be collected by a trained research assistant, blinded to intervention allocation and distinct from the research assistant who will perform enrollment, using an 18-month time frame. All retrospective data collection will use structured methods.[110] In prior studies, we have consistently obtained consent and longitudinal follow-up for >90% of HIV-diagnosed patients from the ED.[57,89,90] See **Appendix** for copies of the **Screening & Enrollment Data Collection Instrument, Clinician Referral Data Collection Instrument, Linkage Navigation Data Collection Instrument**, **ACASI Survey Instrument**, and **Longitudinal Outcomes & HCV Care Continuum Data Collection Instrument**.

F. Outcome Measures

The primary outcome will include initiation of HCV treatment within 12 months from the time of ED diagnosis (for new HCV diagnoses) or ED visit (for those identified with untreated, active HCV) as defined by those with a positive HCV RNA test (active HCV). Secondary outcomes will be: (a) linkage to an HCV treatment provider within 12 months of ED diagnosis (for new HCV diagnoses) or ED visit (for existing HCV diagnoses); (b) for individuals who self-identify as PWID, initiation of substance use disorder services within 12 months of ED diagnosis (for new HCV diagnoses) or ED visit (for those identified with untreated; (c) completion of a full course of HCV treatment with DAAs within 12 months of ED diagnosis (for new HCV diagnoses) or ED visit (for existing HCV diagnoses); (d) sustained virologic response 12 weeks (SVR12) after completing treatment with DAAs within 12 months of ED diagnosis (for new HCV diagnoses) or ED visit (for existing HCV diagnoses); and (e) all outcomes within 18 months of ED diagnosis or visit. Rates of SVR12 will be measured by report of undetectable HCV RNA Nucleic Acid Amplification Test (NAAT) 12 weeks after completion of DAAs among those identified with active HCV.[29] Individuals without evidence of a HCV-associated visit within 12 months from ED diagnosis or ED visit will be considered not linked to care. All outcome measures will be collected and verified via electronic medical record review by trained research assistants blinded to study allocation.

G. Data Management

Data will be entered into a secure electronic database (REDCap, Vanderbilt University, TN) that will be developed to maximize valid data entry by including closed-response entries and range restrictions. Data will then be transferred into native SAS format and cleaned prior to performing analyses using SAS. The dataset will be locked and all analyses performed by the study's biostatistician in conjunction with the principal investigators while blinded to study allocation.

H. Analytic Plan

All analyses will be performed using the intention-to-treat principle. Bivariate statistical tests will be used to compare variables, including results from the ACASI surveys between study groups. Given the randomized design, the primary comparison will include the absolute percentage difference and risk ratio with 95% CIs for initiation of HCV treatment (primary outcome), and tested using chi-square or Fisher's exact test. Kaplan-Meier and Cox proportional hazard regression will also be used to estimate associations between the interventions and outcomes when modeled using time-to-event. One interim effectiveness analyses is planned and will be performed by the study's biostatistician while maintaining blinding. Using the approach by O'Brien-Fleming, the interim analysis will occur at the study's halfway point, after 140 total patients (approximately 35 per arm) have been enrolled and outcomes data collected, and with an effectiveness threshold of $p<0.0054$ for the primary outcome. A non-binding futility threshold of $p>0.5$ will be used for the primary outcome. If the trial is not stopped after the interim analysis, it will proceed to enroll the full sample (280) with a significance effectiveness threshold of $p<0.0492$. The unit of analysis will be the patient. Secondary analyses will include comparisons of all other outcomes by study arm with subgroup analyses for the stratum of individuals <40 years of age or PWID, age, gender, race, ethnicity, income, and education level.

See **Statistical Analysis Plan** for a consolidated description of all planned analyses.

I. Sample Size Estimation

The primary superiority hypothesis for the linkage trial is powered based on a conservative estimate of HCV treatment initiation of 10% using clinician referral with a hypothesized effect of 20% (to 30%) for those allocated to the linkage navigation intervention. We powered this trial based on the <40 years of age or PWID stratum to ensure adequate power. As such, we estimate requiring a minimum of 140 patients (70 per arm) in each stratum to achieve a power of 80% and an alpha of 0.0492 using O'Brien-Fleming stopping rules for one planned interim analysis. No adjustments will be made for loss to follow-up as patients will be considered not linked to care after 12 months from the time of diagnosis or entry into this trial (**Figure 4**).

Figure 4. Sample size estimates for Aim 2, The DETECT HCV Linkage Trial.

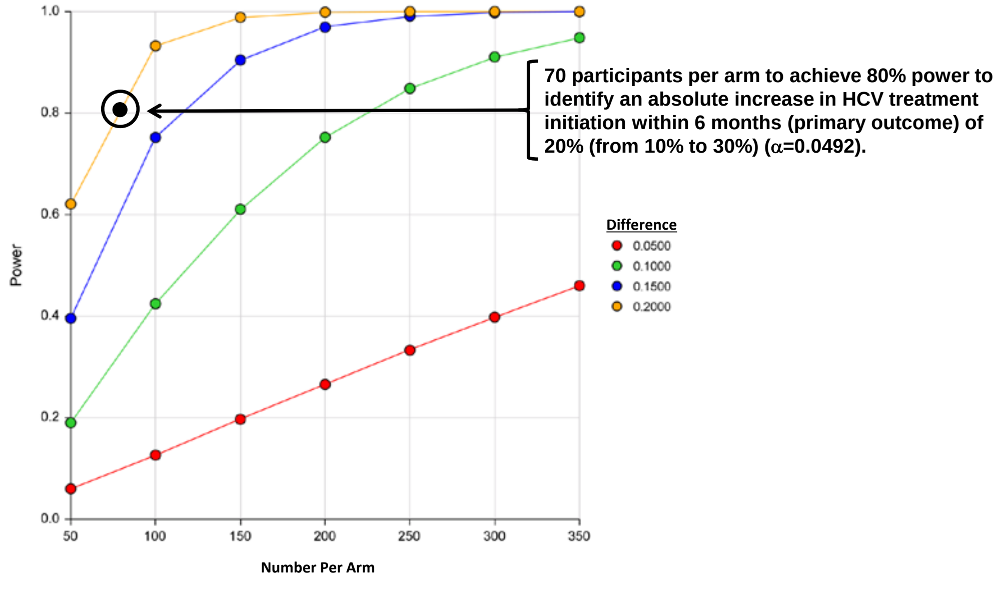

AIM 3 – "Cost Effectiveness"

A. Study Design

We will use program costs and trial results to compare screening and linkage strategies from the perspective of the institution while also projecting long-term costs and cost-effectiveness from a societal perspective using an established Monte Carlo transition-state model, the Hepatitis C Cost Effectiveness Model (**HEP-CE**).

B. Setting

Same as for Aims 1 and 2.

C. Population

Same as for Aims 1 and 2.

D. Interventions

Same as for Aims 1 and 2.

E. Data Collection and Research Procedures

During this aim, we will collect data from Aims 1 and 2 to populate HEP-CE in order to assess the cost effectiveness of screening and linkage strategies. The HEP-CE model is already populated with baseline parameters related to HCV fibrosis progression, liver-related mortality, quality of life, and costs of routine medical care (**Table 3**). These parameters generate realistic output that captures the life expectancy and clinical events of a cohort of HCV-infected individuals and form the foundation of several existing publications.[1,2,86,120] The HEP-CE modeling team reviews model parameters on a regular basis such that they reflect the most up-to-date and accurate data and any changes made to the model between now and the time of the cost-effectiveness analysis will be included in our simulation of the long-term outcomes from DETECT Hep C.

<u>HCV testing and linkage to care</u> – We will use DETECT trial data to inform the costs and effectiveness of the 2 HCV screening and 2 linkage strategies. Importantly, the rich data collected from both trials will allow us to leverage all of the detail in HEP-CE of screening and linkage to care to realistically simulate the trade-offs between the different strategies. <u>HCV therapy</u> – HEP-CE includes treatment parameters for every available DAA regimen. The data come from real-world effectiveness studies when possible or from randomized clinical trials.

<u>Costs of screening and linkage interventions</u> – We will prospectively measure the costs associated with the different screening and linkage strategies of the DETECT trial, using accounting records and <u>time-motion methods</u>. First, we will conduct key informant interviews with study and ED staff to confirm all components of resource use in screening and linkage, while leveraging our experiences with The HIV TESTED Trial; program costs will include: (1) startup (information technology, training); (2) personnel (administrative, ED staff, laboratory staff, and linkage-to-care staff, including linkage navigators); and (3) supplies and equipment (informational sheets, HCV test kits, blood draw supplies). Personnel costs will be determined by identifying position titles of those involved in each part of the interventions and collecting time-motion data using structured data collection. Time-motion data will be collected by trained research assistants during randomly selected time blocks, including nights and weekends, to estimate costs associated with these interventions in EDs. Time per

patient will be translated into time per month and per year, and by position, which will be linked to median salary data from the Bureau of Labor Statistics for each metropolitan statistical area.[121] Metrics of the HEP-CE model throughput will be measured in this aim. See **HEP-CE Model Data** above and **Appendix** for **Time Motion Data Collection Instrument**.

F. Outcome Measures

Outcomes for the economic evaluation will include new HCV diagnoses, rates of linkage to care and treatment initiation, and quality-adjusted life-years (**QALYs**).

G. Data Management

Time-motion data will be collected using the **Time Motion Data Collection Instrument** and then entered into an electronic database (REDCap, Vanderbilt University, TN).

H. Analytic Plan

To inform implementation at the institution level, we will report programmatic costs for each combination of HCV screening and linkage strategies, using total direct costs, newly-diagnosed HCV, linkage to care and initiation of HCV treatment as intermediate outcomes, using methods previously reported by our team.[122] We will then develop parameters for the simulation model (**Table 3**), using HEP-CE to assess the cost-effectiveness of HCV screening and linkage to care in EDs. We will define demographic and disease stage characteristics of the simulated patients using characteristics of the real-world trial participants, and will simulate the lifetime progression of the cohort assuming the current standard of care (no ED-based HCV screening), then assuming each of the following screening and linkage strategies: (1) standard of care + ED-based targeted screening + clinician referral; (2) standard of care + ED-based targeted screening + linkage navigation with clinician referral; (3) standard of care + ED-based nontargeted screening + clinician referral; and (4) standard of care + ED-based nontargeted screening + linkage navigation with clinician referral. The analysis will assume a payer perspective on costs, a lifetime time horizon, and a 3% annual discount rate for both costs and benefits.[123] Outcomes from the model will include numbers of cases of HCV identified, linked to care, initiating HCV treatment, and attaining SVR, and life expectancy, discounted quality-adjusted life expectancy, discounted lifetime medical costs, and incremental cost-effectiveness ratios (ICERs). We will calculate ICERs using standard methodology and will rank strategies in ascending order of lifetime medical cost and then calculate the ICER for each strategy as the additional cost divided by the QALYs gained compared to the next less costly strategy.[123-125] We will assume a societal willingness-to-pay of $100,000 per QALY, where strategies below that threshold are considered "cost-effective."[126]

Table 3. Hepatitis C Cost Effectiveness (HEP-CE) model inputs for The DETECT HCV Trial.

	Value	Source
**HCV Disease Progression**		
Time to cirrhosis (median)	25 years	(128, 129)
Time to liver-related event (median)	11 years	(130)
Mortality with cirrhosis	1.39 deaths/100PY	(130)
Mortality with end-stage liver disease	12 deaths/100PY	(130)
Annual progression rate F0→F1	0.128	(131)
Annual progression rate F1→F2	0.059	(131)
Annual progression rate F2→F3	0.078	(131)
Annual progression rate F3→F4	0.116	(131)
**HCV Screening and Linkage to Care**		
Targeted: Offer & accept	TBD	Aim 1
Targeted: HCV prevalence	TBD	Aim 1
Targeted: Costs	TBD	Aim 1
Nontargeted: Offer & accept	TBD	Aim 1
Nontargeted: HCV prevalence	TBD	Aim 1
Nontargeted: Costs	TBD	Aim 3
Clinician referral	TBD	Aim 2
Linkage navigation + clinician referral	TBD	Aim 2
Linkage: Costs	TBD	Aim 3
**Therapy**		
Initiation of therapy	TBD	Aim 2
SVR (%)	96-99*	(25, 27, 28)
**Non-HCV medical costs, $ per month**		
Background medical costs	$140-$1,050	(132)
**HCV medical costs, $ per month**		
No cirrhosis	$245	(133)
Mild to moderate cirrhosis	$440	(133)
Decompensated cirrhosis	$830	(133)
Cost multiplier after achieving SVR	0.50	(133)
**Quality of Life with HCV infection**		
No-to-moderate fibrosis	0.89	(134-136)
Cirrhosis	0.62	(134, 135)
Decompensated cirrhosis	0.48	(134, 135)

Abbrev: PY = patient years; SVR = sustained viral response; TBD = to be determined. *SVR depends on regimen type, disease genotype, treatment experience, and fibrosis level.

When we have completed the "base case" cost-effectiveness analysis, we will complete an exhaustive series of sensitivity analyses to investigate the degree to which uncertainty in parameter values could change the qualitative cost-effectiveness conclusions, as well as to identify those parameters which have the greatest impact on cost-effectiveness. First, we will conduct a series of one- and two-way deterministic sensitivity analyses. For one-way sensitivity analyses, we will systematically vary each of the parameters in the model across its feasible range and observe how the ICER of each strategy changes. For two-way sensitivity analyses, we will identify pairs of related parameters (e.g., prevalence of HCV among PWID and the probability of test offer to PWID) and simultaneously range both to observe their combined effects on cost-effectiveness conclusions.

See **Statistical Analysis Plan** for a consolidated description of all planned analyses.

I. Sample Size Estimation

Sample size for this aim will be principally driven by sample sizes for aims 1 and 2. However, we will collect approximately 30 observations, or until saturation occurs, for each screening and linkage process component to inform costs of activities.

Aim 4 – "Disparities and Social Determinants of Health"

A. Study Design

We will perform a prospective cohort study nested in the parent DETECT Hep C Screening and Linkage-to-Care Trials to understand SDoH among individuals identified with HCV in the ED and their associations with linkage-to-care, treatment initiation, and treatment completion/cure.

B. Setting

Same as for Aims 1 and 2.

C. Population

Patients eligible for this study will include those eligible for inclusion in the Linkage Trial at Denver Health or those eligible for the screening trial at JHH and UMMC and test positive for HCV antibodies and RNA. Exclusions will include: prisoners and individuals who live outside of the geographic region (given follow-up constraints), non-Spanish or English speaking (as the self-interview for participants will only be available in these languages), and those who are pregnant (because HCV treatment is not currently approved for pregnancy).

D. Intervention

Given the observational nature of this study, no intervention will be performed.

E. Data Collection & Research Procedures

During scheduled study enrollment, research assistants will offer an ACASI survey to all participants after the return of positive antibody results and enrollment in the Linkage-to-Care Trial at Denver Health or enrolling in the SDoH study at JHH or UMMC. The survey will be administered in English and Spanish utilizing. The survey will also include geo-demographic characteristics, housing status, substance abuse, HCV knowledge, willingness to engage in care, health insurance status, and past healthcare usage, as well as perceived stigma and anticipated barriers to care. We will also incorporate domains from the Health Belief Model as an established and validated construct of adherence to care. The survey will be constructed to allow completion in an ED setting, when applicable, and participants will be compensated with gift cards for their time spent completing the ACASI. Reponses will be linked to healthcare system variables, including linkage-to-care (clinician referral vs linkage navigator), site of planned HCV linkage, and duration of time between ED visit and scheduled visit, among others. Participants will be given a unique identifier and answers will be entered anonymously into the ACASI. In addition to the variables described above, the survey will include the following specific details related to SDoH: age, gender, race, ethnicity, sexual orientation, payer information, transportation access, history of incarceration, history of depression, anxiety, and mood disorder, employment, income, education, substance use, and social connectedness and support. Reponses will be linked to HCV treatment related outcomes including successful linkage-to-care, treatment initiation, and completion of HCV treatment using electronic health records. Other information relevant to their care including any linkage interventions will also be included. Participants will be given a unique identifier and responses will be entered anonymously into the ACASI. All results will be stored in REDCap and subsequently exported to SAS for analyses.

F. Outcome Measures

The primary outcome for this aim will include HCV care linkage within 12 months of ED diagnosis or ED visit. Secondary outcomes will include: (a) HCV treatment initiation within 12 months; and (b) SVR12 within 12 months. Individuals without evidence of a HCV-associated visit within 12 months from ED diagnosis or ED visit will be considered not linked to care. All outcome measures will be collected and verified via electronic medical record review by trained research assistants blinded to study allocation.

G. Data Management & Research Procedures

Using the same methods described in Aim 2, data will be entered into a secure electronic database (REDCap, Vanderbilt University, TN). They will then be transferred into native SAS and MPlus formats prior to performing analyses. The dataset will be locked and all analyses for this aim will be performed by Musheng Alishahi, MSc, a doctoral candidate in epidemiology, in conjunction with the principal investigators and the study biostatistician.

H. Analytic Plan

To evaluate disparities, we will include comparisons of all outcomes by study arm by gender. We will specifically report linkage, treatment adherence, and cure rates (with 95% CIs) by gender and use multivariable logistic regression to estimate the effect modification of linkage navigation on gender on outcomes. Multivariable logistic regression analyses will be used to estimate the independent associations between gender and study outcomes while adjusting for confounders, including but not necessarily limited to age, race, ethnicity, housing status, HCV knowledge, and willingness to engage in care. Additionally, effect modifiers (e.g., race, ethnicity and gender, age and gender) will be evaluated.
To evaluate social determinants of health, we propose two statistical approaches to gain a comprehensive understanding of associations between SDoH and HCV linkage to care and treatment, developing distinct models for each outcome. The first statistical approach is variable centered and will use hierarchical multivariable logistic regression to estimate associations between SDoH characteristics and linkage to care, treatment initiation, and SVR12. We will use a theoretical framework and prior studies to inform covariate selection to assess the relationships between SDoH and the outcomes. While this approach informs the overall associations of SDoH variables with the outcomes, it does not address variations in the strength of this relationship across patients and may not fully account for potentially complex inter-relatedness of the SDoH constructs. We will therefore supplement the regression approach with person-centered techniques that will provide a more thorough understanding of SDoH on the HCV care continuum, knowing that these relationships are complex and multidimensional.

Thus, in the second approach, we will perform latent class analysis (**LCA**), a more patient-centered approach. This type of analysis will allow us to identify constellations of patterns of SDoH within the study population by similarities and differences in self-reported SDoH metrics. LCA uses response patterns to identify subgroups or latent classes and is a commonly use method in social epidemiology. LCA typically requires iterations to estimate multiple models using an increasing number of classes. We will begin with a one-class model and increase the number of classes by one until a meaningful number of classes is identified, based on the model log likelihood value and class size (e.g., class size will not have less than 5% of the study population). We will choose a final class model based on statistical fit (i.e., using the lower values for Akaike's Information Criteria [**AIC**], Bayesian Information Criteria [**BIC**], Consistent Akaike's Information Criteria [**CAIC**], and the Approximate Weight of Evidence Criteria [**AWE**]) and meaningful theoretical interpretation. LCA will be performed using structural equation modeling software (MPlus Version 8.4, Muthen & Muthen, Los Angeles, CA).

From LCA, each study participant will be assigned modal class assignment, where class membership will be represented as a nominal variable corresponding to the number of classes (i.e., 1, 2, 3, etc.). This nominal variable will then be assessed in relationship to each outcome, separately, using logistic regression, and applying rigorous and theoretically sound methods for addressing classification imprecision. Other covariates in this model will include demographic information (e.g., age, gender, and race, ethnicity). We will then directly compare the results from the conventional multivariable logistic regression approach to the LCA approach through the prediction and estimated precision of the primary and secondary outcomes. However, we view the two approaches as complementary, so we are not focused on the direct comparison of the two methods in terms of outcome prediction, but rather to use of both methods to gain a more complete understanding of the role of SDoH in explaining the HCV care continuum. LCA is an empirically based technique that assumes heterogeneity of the study population. We anticipate sufficient heterogeneity in the SDoH indicators to support LCA; however, if the expected heterogeneity is not borne out by the data (i.e., a single class model provides superior fit), we will revert to the multivariable logistic regression models as sufficient for understanding the association between SDoH and the outcomes.

Lastly, we will attempt to replicate latent classes across all sites in an effort to capture homogenous classifications that may identify subpopulations experiencing differential HCV treatment outcomes. This approach will use an experimental method of applying empirical and theoretical driven model constraints to obtain model replication. The LCA modeling from the previous aim will inform model constraints in replication and cannot be identified *a priori*.

I. Sample Size Estimation

To achieve the aims proposed in the gender disparities portion of this project, we will target primary enrollment as described above but may enrich the sample to optimize balance between genders. Sample size calculations for LCA do not have a formal approach as in cases of more conventional statistical approaches (e.g., ANOVA or regression) and are largely reliant on simulation studies. Power analysis in LCA is important to identify the number of classes best suited for the data and will depend on the prevalence, number of indicator variables, and classes involved in the model. According to prior simulation studies, a sample size of 79 would be sufficient for a three class model with 10 measured variables to achieve 90% power. Other studies have found that a sample sizes between 250 and 500 are suited to identify necessary classes. Thus, for this aim, we will include 8 measured items into LCA and recruit approximately 400 patients to ensure heterogeneity of the population while also achieving sufficient statistical power. The anticipated sample size from JHH and UMMC will be collectively 250 with approximately 125 participants from each site. As previously stated, prior simulation studies have recommended samples between 250 and 500 is sufficient for identifying classes. Using this information, we anticipate having sufficient power to identify classes in the replication analysis.

7. HUMAN SUBJECTS PROTECTIONS

A. Institutional Review Board

The Colorado Multiple Institutional Review Board (**COMIRB**) will act as the single IRB of record (**SIRB**) for The DETECT Hep C Trial. DHMC/COMIRB will initiate reliance agreements with each site participating in the trial (Johns Hopkins Hospital and University of Mississippi Medical Center) through the SMART IRB mechanism. All sites will be responsible for conducting a local context review, to ensure that the protocol is appropriate and reasonable for their respective study populations, however the COMIRB approval will serve as the IRB approval of record for all sites and the entire study. COMIRB will provide a letter of support to serve as the SIRB, a templated letter of support for relying sites to complete, a draft protocol to be adapted for local context, FWA instructions for participating sites, as applicable, and, SMART IRB instructions for participating sites.

The protocol and consent form will be reviewed and approved by COMIRB and each participating center will perform a local context review prior to study initiation. Serious adverse experiences regardless of causality will be reported to the SIRB in accordance with the standard operating procedures and policies of the SIRB, and the study team will keep the SIRB informed as to the progress of the study. Any documents that the SIRB may need to fulfill its responsibilities (such as protocol, protocol amendments, consent forms, information concerning patient recruitment, payment or compensation procedures, or other pertinent information) will be submitted to the SIRB. The SIRB written unconditional approval of the study protocol and the informed consent form will be in the possession of the Investigator before the study is initiated. Enrollment will not begin at either site until SIRB approval of that site has been secured. The IRB unconditional approval statement will be transmitted by the primary site (DHMC) to each site investigator prior to the shipment of study supplies to the site. This approval must refer to the study by exact protocol title and number and should identify the documents reviewed and the date of review.

Protocol and/or informed consent modifications or changes may not be initiated without prior written SIRB approval except when necessary to eliminate immediate hazards to the patients or when the change(s) involves only logistical or administrative aspects of the study. Such modifications will be submitted to the SIRB and written verification that the modification was submitted and subsequently approved should be obtained. The SIRB must be informed of revisions to other documents originally submitted for review; serious and/or unexpected adverse experiences occurring during the study in accordance with the standard operating procedures and policies of the SIRB; new information that may affect adversely the safety of the patients of the conduct of the study; an annual update and/or request for re-approval; and when the study has been completed.

B. Descriptions, Risks, and Justification of Procedures

Recruitment Methods

Aim 1 – “Emergency Department Screening Trial”

Eligible patients will be offered rapid HCV screening using 1 of 2 screening interventions allocated in a 1:1 random sequence. Rapid HCV screening itself represents standard care in each of the EDs participating in this study, while the process by which the screening occurs represents the intervention. Because HCV testing will be performed as a part of routine medical care, a waiver of written informed research consent will be sought for this portion of the project, similar to the approach we took when using similar methodology to evaluate: (1) nontargeted opt-out rapid HIV screening in the ED at Denver Health from 2007-2009; and (2) 3 forms of HIV screening in the EDs at Denver Health Medical Center, Highland Hospital, Johns Hopkins Hospital, and the University of Cincinnati Medical Center from 2012-2016. Furthermore, because screening and consent mechanisms for rapid HCV testing will be integrated into routine ED care, this aspect of the study will be minimal risk, and obtaining written informed consent would not be feasible and likely would bias participation for the large number of patients planned for this study. We believe that the waiver of consent will not adversely affect the rights and welfare of the subjects involved in this project. Consent for the performance of rapid HCV testing will be documented as part of routine medical care.

Consent Procedures

Patients who present to the ED during this project will receive standard-of-care medical evaluation and treatment and may be asked questions about their risk for HCV infection, and may be offered, as voluntary and routine practice, rapid HCV testing using an opt-out consent mechanism. Consent for rapid HCV testing will be integrated into routine emergency medical care as is currently standard. The medical care for those patients who do not receive rapid HCV testing will not vary from those who do, except that the physician will know each patient’s HCV test result and may alter the medical evaluation

based on this additional information. Because rapid HCV testing is a standard of care and because this project will be evaluating 2 processes for performing HCV screening in this clinical setting (i.e., nontargeted vs. targeted screening), this potential change in care is consistent with current medical practice.

We will request a waiver of consent for everyone included as part of the evaluation of the 2 HCV screening strategies and as part of the longitudinal follow-up. This request for waiver is based on the following in accordance with 45 CFR 46.116(d)(1-4):

(1) This part of the study is minimal risk because the only risks include breach of confidentiality and possible risks of change from standard of care (although each screening method is consistent with recommended national or local standards of care, and all data collected in follow-up will be from medical records documenting standards of care for those who are linked into care for HCV infection). The most significant risk to patients will therefore be loss of confidentiality, which is viewed as minimal. All of the methods utilized in this study are currently accepted, recommended and utilized methods for HCV screening, locally or nationally. Furthermore, there is minimal difference in risk between the 2 screening interventions. While there is a potential risk associated with missed opportunities for HCV diagnosis, we believe that this potential risk is also minimal. We also believe that clinical equipoise exists in that there is no empiric evidence that 1 HCV screening method is superior to another despite policy statements (e.g., CDC and USPSTF) that recommend targeted screening; furthermore, the effectiveness of HCV screening is even more uncertain in ED settings. Patients who access testing services in routine health care settings are exposed to this same risk. Therefore, the risks involved in this study are no more than what the patient would experience in standard care. The principal investigators will assume full responsibility for the protection of all study-related documents and datasets, including those that contain protected health information. The principal investigators, project manager, and project coordinators will oversee all data collection. All electronic data will be stored using the protected network at each institution and each database will be password protected and stored in the project coordinators' networks using separate folders on mainframe networks of the institutions. These networks include firewall protection and the folder will only be accessed by members of the study team. All paper documents will be stored in a locked file cabinet in the project coordinators' offices. These data will be manually entered into the main electronic database. All data will ultimately be transferred to the DCC and the same data protection methods will be employed by the project manager/data coordinator. During this project, all data will be kept in secure locations either in the ED, the offices of the principal investigators, the office of the project coordinator, or in the laboratory;

(2) It does not violate patients' rights (the research will offer HCV testing by asking standard-of-care questions but the patient will still be able to decide whether HCV testing occurs). The risks involved in this study are no more than what the patient would experience in standard care and each patient will be able to decide whether HCV testing occurs**;**

(3) Separate written informed consent cannot practicably be carried out without a waiver because of the large number of consecutive patients included from each ED, and the potential for biased participation in the study. It is essential that we have 100% participation in this study in order to identify the rate of newly diagnosed HCV infection, which is the primary outcome for this study. We believe this waiver of consent will not adversely affect the rights and welfare of the subjects involved in this project, as they will only be subjected to current standards of care and they will all have the right to refuse HCV testing without adversely affecting their care; and

(4) Each patient will have an experience that does not differ significantly from usual care.

Risk Assessment Survey: A representative sample of patients will be approached by a research assistant to participate in this sub-study. We will administer a closed-response survey of those who verbally consent and will collect a series of de-identified risk behavior data. No patient identifiers will be collected as part of this sub-study. All research procedures, therefore, will be minimal risk.

Aim 2 – "Linkage-to-Care Trial"

Verbal informed research consent will be obtained from all patients enrolled in this trial. A principal goal of this trial is to evaluate the effectiveness of 2 different linkage-to-care strategies and will require collection of longitudinal research data, including information pertaining to stage of disease, linkage-to-care, initiation of treatment, and any hospitalizations or unscheduled medical visits for the 18 months following diagnosis. Study specific research assistants, who have completed IRB training, will screen for and enroll all patients in this trial.

Enrollment and obtaining informed consent will take place in a quiet and unhurried setting in the patient's room either in-person or over the phone. The patient will be given enough time to ask questions about study procedures, data collection, and follow-up. The patient's comprehension will be assessed by asking him or her to describe the procedures in his or her own words.

Certificate of Confidentiality (**CoC**): As an NIH-funded trial, this protocol has been issued a CoC from the federal government to help protect patient privacy. The CoC prohibits the researchers from disclosing patient name, or any identifiable information, document or biospecimen from the research. This certificate provides protections against disclosing research information in federal, state, or local civil, criminal, administrative, legislative or other proceedings. The CoC certifies that all information collected as part of research, including the ACASI and information disclosed as part of linkage navigation for those patients assigned to that arm of the trial, will be protected and disclosure of sensitive information to anyone outside of the research team without consent from the patient will be strictly prohibited and will not occur.

Verbal Consent Procedures: We will request a waiver of written documentation of informed consent. This request for waiver is based on the following in accordance with 45 CFR 46.117(c): This research presents no more than minimal risk of harm to subjects and involves no procedures for which written informed consent is required outside of the research context.

Verbal informed consent will be obtained either during a face-to-face interaction or over the phone if such interactions are restricted due to institutional policies for the conduct of research during the COVID-19 pandemic or if the patient is unable to be approached during his or her ED visit. If face-to-face interaction is not feasible then research staff will call the patient to make an introduction and to describe the study. A verbal consent script will be used to ensure that all of the elements of informed consent are used. The patient will be given enough time to ask questions about study procedures, data collection, and follow-up. The patient's comprehension will be assessed by asking the patient to describe the procedures in his or her own words and patients will be asked to affirm that they provide verbal consent to participate in the study.

In general, underserved and homeless communities are overrepresented among ED patients, and are particularly susceptible to HCV. These populations are also more likely to lack access to smartphones and other technologies that are often used for virtual or remote eConsent procedures. While eConsent procedures offer many benefits for conducting clinical research, widespread implementation of such

procedures may reduce equitable access across the socioeconomic spectrum, which is a particular concern when recruiting participants in EDs.[127] Additionally, given the nature of an around-the-clock care environment in EDs, potentially eligible patients often present during days and times that research staff are not able to approach in person. The use of verbal consent procedures would improve health equity by increasing access to this trial among individuals who do not present to the ED when research staff are available or do not have a smartphone or the technology needed to participate in a virtual/remote consent procedure.

Aim 3 – “Cost Effectiveness”

For the purposes of this aim, no individual patient-level data will be collected. The data collected will only represent time-motion data including amount of effort required for the 2 HCV screening strategies and rapid HCV testing procedures by ED and hospital personnel. Because no identifiable individual private information will be collected as a part of this aim, we do not believe the data collected meets the definition of human subjects’ research. There is no risk of loss of confidentiality as none of the data contains identifiable information.

Aim 4 – “Disparities and Social Determinants of Health”

Similar to Aim 2 at Denver Health, informed research consent will be obtained verbally at JHH and UMMC from all patients enrolled in this study in order to gather information on SDoH and to obtain additional longitudinal research data including information pertaining to stage of disease, linkage-to-care, initiation of treatment, and any hospitalizations or unscheduled medical visits, for the 1 year following diagnosis. During enrollment hours, study specific research assistants will screen for and enroll all antibody positive ED patients in this trial. They will have completed IRB training and will obtain verbal informed consent from all participants before administering the survey. This process will take place in a quiet and unhurried setting in the patient’s room. The patient will be given enough time to ask questions about study procedures, data collection, and follow-up. The patient’s comprehension will be assessed by asking the patient to describe the procedures in their own words.

Special Consent Issues: Vulnerable Populations
Populations considered vulnerable that will be included in this project are pregnant women and prisoners.

Pregnant women will not be specifically targeted for participation in this project. However, it is possible that a pregnant woman may be offered and accept rapid HCV testing as a part of this project. Inclusion in this project will not present more than minimal risk to pregnant women. The potential benefit of participation in this project is that women who are infected with HCV may be identified and linked-to-medical care, which will benefit not only the mother, but also potentially the fetus. Treatment for HCV is not currently approved during pregnancy, however if HCV infection is identified, closer prenatal monitoring can occur and efforts to prevent mother-to-child transmission during delivery can be initiated. Due to the fact that treatment for HCV is not approved during pregnancy, pregnant women will be excluded from Aim 2 and Aim 4 since they are not eligible for the primary outcome measure of this aim (HCV treatment within 12 months).

Prisoners will be included in Aim 1 but not Aim 2 or Aim 4. There is no issue related to the rights of human subjects in this study that would warrant the exclusion of prisoners. The screening portion of the study is standard-of-care, the follow-up portion consists only of medical record reviews and both portions are minimal risk. Including prisoners may provide them the benefit of knowing if they have chronic HCV infection. Participation of prisoners in this study will only include those prisoners who

present for medical care at each of the EDs involved in this trial - recruitment and research-related follow-up will not occur at a prison. The specific interventions being evaluated in this study relate only to how patients are identified for and offered HCV testing; all interventions are currently local or national standards-of-care. All of the testing procedures that occur after patients are identified for HCV testing (i.e., consent for test, performance of the test, results disclosure, linkage to care, etc.) are fully integrated into the clinical care and are standards-of-care, including processes that are in place for prisoners. The current clinical processes for prisoners who are newly identified with HCV vary across institutions; however, each site addresses the following: standard medical care, physician-directed results disclosure, and the appropriate steps towards linkage-to-care processes. Any specific procedures currently in place at the institutions involved with this trial will not change for prisoners who participate in the research.

For prisoners who are enrolled to Aim 1 and test HCV antibody positive, before discharge the result will be disclosed by provider to patient. The hospital discharge summary will include result and follow up information and be sent with prisoner/accompanying officer to the prison after discharge (**Appendix – HCV Preliminary Positive Discharge (AVS) Information**). This documentation will be submitted to medical officer or charge nurse at the facility. Further a member of the research team will monitor RNA confirmatory results which result within 3 - 5 days and will call the charge nurse/medical officer at the correctional facility to update the healthcare provider of the detainee's medical management needs and to assure adequate follow-up. Research staff will provide medical staff at correctional facilities with HCV information to distribute to prisoners who are being released and wish to follow up with HCV care (**HCV Information for RNA Positive Prisoners**). This will be the results disclosure procedure at Aim 1 sites who are enrolling prisoners.

In accordance with HSS regulations 45 CFR 46.305(c) and 46.306(a) COMIRB will certify to the Office for Human Research Protections (**OHRP**) on behalf of sites who are enrolling prisoners to Aim 1. Each site’s approval of involvement of prisoners in Aim 1 is contingent upon local IRB composition requirements set forth by 45 CFR 26 (b). This requires that at least one member of the local IRB must be a prisoner representative with appropriate background and experience to serve in that capacity. Those sites whose local IRB do not meet these requirements will exclude prisoners from enrollment in Aim 1.

This study is minimal risk and there is no reason to exclude prisoners as: (1) participating does not provide them with any advantages that are so great as to impair their ability to weigh the risks and benefits of participating; (2) the risks of participating are equal to non-prisoner patients; (3) selection of participants will not occur within prisons and will only occur if a prisoner presents to the ED to receive urgent/emergency medical care; therefore selection will be free from intervention by prison authorities or other prisoners; (4) there is no control group in this study (both intervention arms are either local or national standards-of-care); (5) the parole board will not take into account prisoner's participation in the study when considering parole because randomization into the 2 different screening arms (and individuals identified as HCV positive as a result of the randomization) has no bearing on a decision for parole; (6) the follow-up portion of this study consists only of obtaining medical records (no actual contact with the prisoner is necessary) and no follow-up treatment is necessary as a result of participating in the research (linkage-to-care services and medical care will continue as necessary for patients identified as HCV positive, however all prisoners receive this standard-of-care, regardless of whether they are research participants or not). Prisoners will not be enrolled in the Linkage Trial.

<u>Children</u> (i.e., individuals <18 years of age) will not be included in this project.

<u>Authorization Procedures</u>

Aim 1 – "Emergency Department Screening Trial"

We are requesting a waiver of HIPAA authorization for all patients included in this study. Authorization for the collection and use of protected health information (**PHI**) will be obtained, however, by using the routine authorization forms utilized in the ED at each institution as part of standard medical care. Data collected as part of this project will be no more than a minimal risk of harm to privacy, and HIPAA authorization cannot be practicably carried out without a waiver due to the large number of patients planned for inclusion and because its requirement may bias participation. In addition, this research cannot be performed without specific requested PHI. This project meets the requirements for a waiver of HIPAA authorization for the same reasons that requirements for a waiver of consent are met.

Risk Assessment Survey: We are requesting a waiver from authorization for patients who participate in the Risks Assessment survey. The survey will be de-identified and represents no more than minimal risk to participants.

Aim 2 – "Linkage-to-Care Trial"

We are requesting a Waiver of HIPAA Authorization with our request to obtain verbal informed consent. This request is based on the following in accordance with 45 CFR 164.152(i)(2)(ii):

A. The use or disclosure of PHI involves no more than minimal risk to the privacy of individuals, and:
   1. There is an adequate plan to protect the identifiers from improper use and disclosure – Data will be entered into REDCap with access granted only to those on the research team and stored in a password protected file in a restricted access folder on the institutions secured network;
   2. There is an adequate plan to destroy the identifiers at the earliest opportunity consistent with the conduct of the research, unless there is a health or research justification for retaining the identifiers or such retention is otherwise required by law – Identifiers will be maintained in order to complete chart abstraction for longitudinal outcomes; once the all data are cleaned and the data set is locked it will be stripped of all identifiers; and
   3. PHI will not be reused or disclosed to another person or entity, except as required by law, for authorized oversight of the research project, or for other research for which the use or disclosure of PHI would be permitted by this subpart.

B. This research could not be practicably conducted without a waiver of HIPAA authorization. In response to the restrictions placed on research due to the COVID-19 pandemic it has become increasingly difficult to have face-to-face interactions with research participants. While eConsent procedures are an option, these technologies are difficult to utilize in a clinical care environment when direct patient contact is limited as much as possible in order to prevent unnecessary exposures to SARS-CoV-2. In addition, there are inequities to access and understanding of the technologies and devices often utilized for eConsent procedures. As described in Verbal Consent Procedures, conducting research in EDs presents unique challenges due to the patient populations often cared for (i.e., underserved, homeless, socioeconomic vulnerable, access to and understanding of technology) and the 24/7 nature of the care environment. Often there are potentially eligible patients that present to the ED when research staff are not available to approach and enroll participants. Given the minimal risk nature of this project, obtaining approval to use verbal consent and to waive HIPAA authorization procedures will provide more equitable opportunities to participation in the Aim 2 Linkage Trial; and

C. This research could not be practicably carried out without access to and use of PHI as it is needed to obtain the data needed for the primary outcome of the Aim 2 Linkage Trial.

<u>Aim 3</u> – “Cost Effectiveness”

Not applicable.

<u>Aim 4</u> – “Disparities and Social Determinants of Health”

We are requesting a waiver of HIPAA authorization with the justification and procedures for Aim 2 as described above.

<u>Data Protection</u>

Each study site will follow the same data collection and transfer procedures to ensure maximal confidentiality of all patients. At each site, investigators will compile the following data, either directly from the ED information systems or from individual data collection instruments designed specifically for this study: (1) patient visit information (name, medical record number, acuity level, mode of arrival, and date/time of visit); (2) demographics (age, sex, race, ethnicity, primary language); (3) payer information (commercial, Medicare, Medicaid, self, or state-sponsored); (4) details of randomization, including the intervention assigned and results of risk screening, if applicable; (5) whether a patient was offered, accepted, and completed rapid HCV testing; (6) results from all rapid HCV tests; and for all patients with a reactive HCV test (7) confirmatory test results, whether they were successfully linked into care, and details of follow-up care and disease progression. Each site database will then be cleaned and have discrepancies resolved prior to replacing names and medical record numbers with unique patient identifiers.

Once each site database has been cleaned and stripped of patient identifiers, it will be transferred electronically to Denver Health Medical Center (i.e., the DCC) using an SFTP. All electronic databases will be stored in Excel (Microsoft Corporation, Redmond, WA) that is password protected and stored on a firewall-protected and highly secure server. Paper records will be stored in locked file cabinets at each individual site. All study-related materials (electronic files or paper records) will be accessible only by study investigators or authorized study personnel.

Procedures will be implemented to assure patient confidentiality in gathering and recording all study-related data, and close oversight of all collection, transfer, and data storage by the Principal Investigator, Project Managers, Data Coordinator, and site investigators. We will also invoke Research Use Agreements between the three study sites and Denver Health. In order to further minimize the risk of breach of confidentiality, study data will not be placed or stored on a laptop computer or other portable storage unit (e.g., “jump drive”). All collected data will follow Denver Health and Hospital Authority security regulations for additional protection. Once complete, the database will be completely de-identified and all study-related documents with protected health information will be destroyed. No external data safety monitoring board will be used for this study.

<u>Special Considerations for Performing Consent in the Setting of COVID-19</u>

Given the unpredictability of the COVID-19 pandemic and the potential risk associated with direct patient contact, procedures to safely and effectively enroll study participants is important. The following outlines modified procedures to maintain enrollment in The DETECT Hep C Trial, while minimizing risk to research staff, participants, or clinical staff.

<u>Aim 1</u> – "Emergency Department Screening Trial"

This trial is fully integrated into standard emergency medical care and does not require adjunctive research personnel to facilitate enrollment; as such, no modifications to enrollment will occur in the context of COVID-19.

<u>Risk Assessment Survey</u>

See "Aim 2" for procedures related to enrollment of participants and data collection.

<u>Aim 2</u> – "Linkage-to-Care Trial"

This trial requires research staff to identify, approach, offer, and enroll participants in the ED. Research staff will adhere to all departmental and institutional policy regarding use of personal protective equipment (**PPE**). We have identified three tiers of patient interaction depending on a patient's SARS-CoV-2 status and potential symptoms related to COVID-19.

> <u>Tier 1</u> (SARS-CoV-2 polymerase chain reaction (**PCR**) positive or symptoms potentially related to COVID-19): Potential eligible patients in this category will not be approached for enrollment.
>
> <u>Tier 2</u> (Symptoms not consistent with COVID-19 and SARS-CoV-2 unknown): Potential eligible patients in this category may be approached, although if approached in person, research staff will use PPE as stipulated by institutional/departmental policy, or virtually by use of telephone, and verbal consent methods as described in **Verbal Consent Procedures** (see **7. HUMAN SUBJECTS PROTECTIONS** – **B. Descriptions, Risks, and Justification of Procedures**).
>
> <u>Tier 3</u> (SARS-CoV-2 PCR negative): Potential eligible patients in this category may be approached in person using protective methods stipulated by institutional/departmental policy, or virtually by use of telephone, and verbal consent methods as described in **Verbal Consent Procedures** (see **7. HUMAN SUBJECTS PROTECTIONS** – **B. Descriptions, Risks, and Justification of Procedures**).

<u>Aim 3</u> – "Cost Effectiveness"

Research staff will be present in the ED for purposes of time motion data collection. However, no direct patient care contact will be made. Research staff will wear face masks at all times while in patient care areas, and conform to all institutional standards related to use of PPE.

<u>Aim 4</u> – "Disparities and Social Determinants of Health"

See "Aim 2" for procedures related to enrollment of participants and data collection.

C. Estimated Duration of the Study

It is estimated that this study will take up to 6 years to complete (up to 5 years study duration and up to 18-month follow-up). For patients enrolled in the "Screening Trial", the estimated duration of their participation will be the length of time spent in the ED. All patients will be followed for up to 18 months from the time of diagnosis to evaluate effectiveness of linkage navigation and clinical referral (**Figure 5**).

D. Number and Distribution of Subjects

The “Screening Trial” will require approximately 50,000 randomized patients across all sites with expected 16,667 randomized patients per site. This will result in an estimated 13,965 completed HCV tests and an estimated 706 confirmed newly diagnosed HCV-infected patients.

Risk Assessment Survey: We will include 450 total participants (150 participants per site).

The “Linkage Trial” will require 432 participants.

The “Disparities and Social Determinants of Health” aim will include all participants from the Linkage-to-Care Trial in addition to 250 participants, split between JHH and UMMC.

E. Examinations, Laboratory Tests, Procedures, and Follow-up Visits

All patients will receive standard medical care during their ED visits. Patients who test preliminarily positive for HCV will receive standard medical care for HCV. This research in no way will change or alter standards of care for those who participate.

All patients who are offered and agree to rapid HCV testing will have blood drawn and sent to the institution’s laboratory. Each patient’s blood sample used for rapid HIV testing will be labeled with the patient’s name, medical record and encounter number.

All rapid HCV testing will be performed using valid and Food and Drug Administration (FDA)-approved HCV testing methods. All of these tests are performed as a part of standard of care. All rapid HCV testing, will be performed by the hospital laboratory with an approximate total turn-around time of 20 – 40 minutes, which includes preparation time, processing time, and reporting time. All HCV preliminary testing results will be provided to the patients during their ED visit.

At Denver Health, patients who test positive will be asked to provide verbal informed consent to enroll into the Linkage Trial (see “**Clinical Referral Data Collection Instrument”, “Screening Enrollment Data Collection Instrument”,** “**ACASI Instrument” or “Linkage Navigation Data Collect Instrument”** in the **Appendix**).

At John’s Hopkins Hospital and University of Mississippi Medical Center, patients who test positive will be asked to provide verbal informed consent to enroll into Aim 4. See **Appendix** for “**ACASI Short Form Survey Instrument**”.

Strengths of this study will be the inclusion of sites that use different implementation methods of HCV testing and linkage-to-care. Several stipulations, however, will be required of each site: (1) implementation and full integration of the interventions and HCV testing processes into the EDs; (2) use of streamlined opt-out consent (but in accordance with state statutes); (3) use of Food and Drug Administration (FDA)-approved rapid HCV testing (e.g., OraQuick® HCV Test, OraSure Technologies, Inc., Bethlehem, PA) to ensure highly accurate and timely result reporting; (4) a process for additional clinician-directed diagnostic HCV testing for those patients not screened, who were identified as low risk during screening, or who declined testing when offered during either of the screening interventions; and (5) performance of RNA testing and appropriate linkage-to-care for those who test positive for HCV antibodies. Given its “pragmatic” nature, other operational features (e.g., which rapid test is used, who performs linkage-to-care, etc.) will not be explicitly stipulated for the sites not participating in the linkage study described in Aim 2. Each institution, however, has extensive experience performing rapid HCV and HIV testing in the ED and has optimized their methods to ensure excellent patient care.

Targeted Screening Risk Assessment will be integrated into each institution's EMR and administered by nurses to all patients who meet criteria for inclusion and are randomized the targeted screening arm. Patients will be given the opportunity to opt-out of the HCV rapid testing as mentioned above. The

Longitudinal Outcomes and HCV Care Continuum Data Collection Instrument will be completed at 18 months following the date of ED diagnosis.

Aim 2 – "Linkage-to-Care Trial"

In addition to the Clinical Referral instruments, participants enrolled to this study will have the following forms completed: **Screening Enrollment Data Collect Instrument**, **ACASI**, and the **Linkage Navigation Data Collection Instrument**.

F. Protected Health Information

Protected health information will be included on most documents used in this study. Such information will include each patient's names, medical record and encounter number, dates of birth, sex, race, ethnicity, date of visit, registration time, and discharge or hospitalization time, and the result of the rapid HCV test, if performed. In addition, for patients who test positive for HCV infection additional information will be collected, including laboratory results, whether patients with positive HCV antibody tests were successfully linked into care, and components of the HCV Care Cascade.

G. Risks

Subjects

The most significant risk to patients will be loss of confidentiality, which is considered minimal. All of the methods utilized in this study are currently accepted, recommended and utilized methods for HCV screening, locally or nationally. All medical centers in Colorado, including DHMC, are required by state law to report all diagnoses of HCV infection to the state health department. Patients who access testing services in routine health care settings are exposed to this same risk. Therefore, the risks involved in this study are no more than what the patient would experience in standard care. The Principal Investigator will assume full responsibility for the protection of all study-related documents, including those that contain protected health information.

Investigators/Institutions

There are no risks to the institutions or investigators.

Aim 1 – "Emergency Department Screening Trial"

The primary risks to patients included in this trial will be breach of confidentiality as all study procedures, including those related to the 2 rapid HCV screening strategies, will be performed as routine medical care in the EDs or as follow-up for those who test positive for HCV antibodies. Results of all HCV tests will be recorded in the patients' medical records as part of standard medical care. Consent for the performance of rapid HCV testing will be integrated into the general ED medical consent for evaluation and treatment. We will seek a waiver of consent for research and will therefore not obtain written informed research consent from patients eligible for screening for the following

Figure 5. The DETECT HCV Trial project timeline (updated: April 15, 2020).

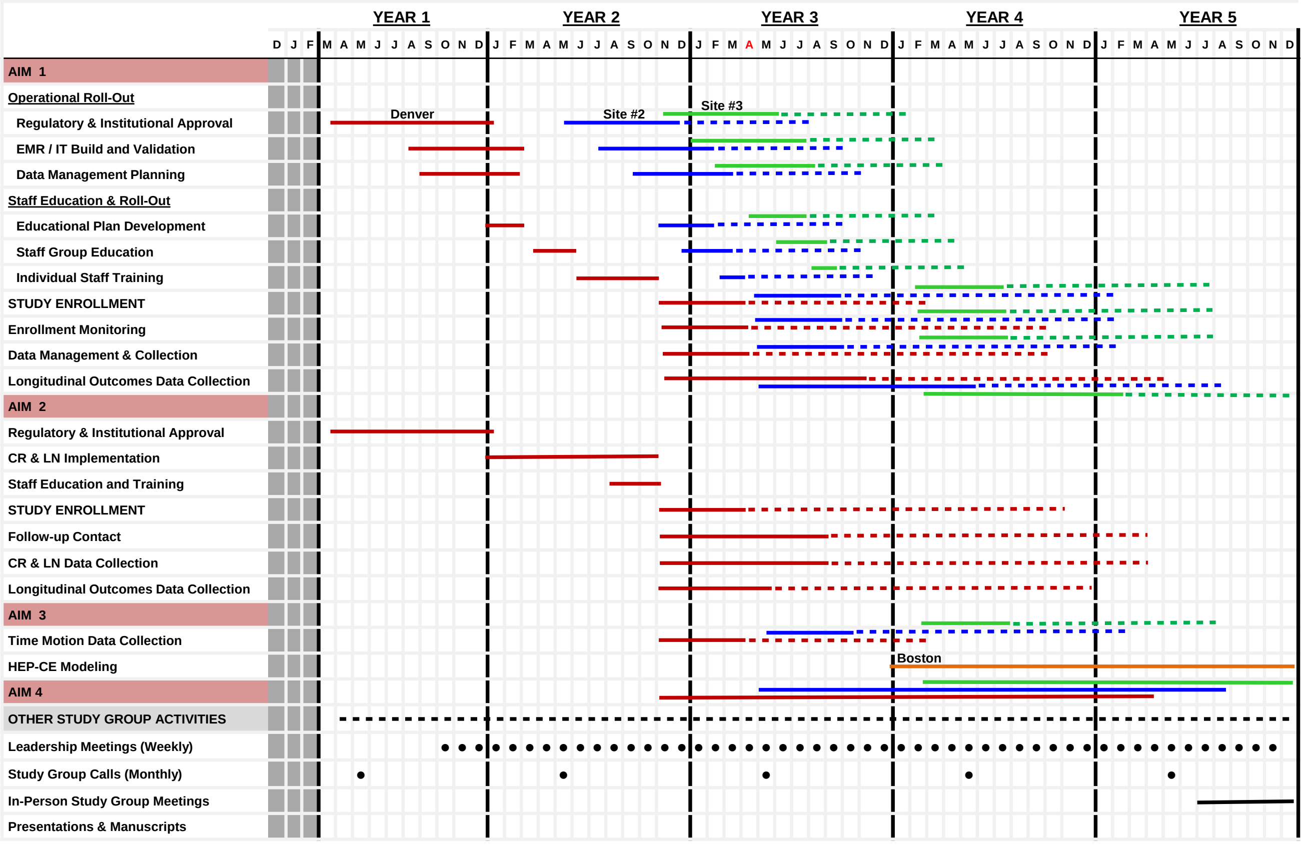

reasons: (1) screening mechanisms will be integrated into routine ED care; (2) this aspect of the study will be minimal risk; (3) written informed consent will not be feasible for such a large number of anticipated patients; and (4) obtaining written informed consent will significantly bias participation in the study where we are evaluating a public health screening intervention. We will also seek a waiver of consent for research and will therefore not obtain written informed research consent from patients who test preliminarily positive for HCV in the ED in order to obtain data related to standard-of-care follow-up and evaluation of disease progression for a period of 1 year following diagnosis. All study procedures related to follow-up will only involve structured retrospective collection of data from medical records from participating institutions; all data will be collected only for purposes of care and this part of the study, therefore, is also considered minimal risk.

Our foremost concern during this study will be to protect patient confidentiality. To ensure this, each patient will be assigned a unique study identification number after electronic and hand collected data are transferred into the study database. The site principal investigators will maintain a list of patients and their unique study identification numbers until data analyses are complete and results have been reported. This list will be kept in a password protected file and only accessible by the study investigators.

Aim 2 – “Linkage-to-Care Trial”

The primary risks to patients included in Aim 2 will be breach of confidentiality similar to Aim 1. In addition to the data collected in Aim 1, identifiable data related to HCV antibody and RNA results, detailed patient contact information, linkage navigation information, and longitudinal health outcome data will be collected via paper data collection instruments and case report forms. Paper data will be stored in a locked filing cabinet in the Project Coordinator's locked office. All study-related materials will be accessible only by study investigators or authorized study personnel. All risks of participation will be clearly outlined in the informed consent document and discussed with patients during the informed consent process.

<u>Aim 3</u> – "Cost Effectiveness"

While the data collected as part of this aim is not considered human subjects research, it is possible that these data could be compromised in some fashion. There is no risk of loss of confidentiality as none of the data contain identifiable information. All institutional and research regulations pertaining to the protection of electronic and paper data will be followed.

<u>Aim 4</u> – "Disparities and Social Determinants of Health"

Similar to Aims 1 and 2, the primary risks to participants included in Aim 4 will be breach of confidentiality. In addition to the data collected for Aim 1, identifiable data related to HCV antibody and RNA results, detailed patient contact information, SDoH information, and longitudinal health outcome data will be collected using electronic instruments (REDCap, Vanderbilt, TN). All study-related materials will be accessible only by study investigators or authorized study personnel. All risks of participation will be clearly outlined in the informed consent document and discussed with patients during the informed consent process.

H. Benefits

The DETECT Hep C Trial will provide direct clinical benefit to patients. HCV is common (~3.5 million infected in the U.S.) and with a large proportion of individuals who remain undiagnosed (~50%); further, HCV-related deaths are more common than deaths from all other reportable infectious diseases combined, and data from the HCV care cascade shows that linkage-to-care and treatment is one of the largest variances in the cascade. Participants may directly benefit from this study by learning their HCV status. In addition, those identified with HCV may benefit from earlier identification, linkage into care, and initiation of treatment, which leads to cure for >95% of individuals. Moreover, others may benefit indirectly from this study because those identified with HCV may modify their behaviors, thus reducing potential for transmission of the virus.

I. Limitations

<u>Aim 1</u> – "Emergency Department Screening Trial"

The primary difficulties with this aim relate to implementation of the HCV screening interventions in 3 high-volume EDs, the large number of included patients, and the consistent and accurate acquisition of data. Our research team has extensive experience evaluating a wide range of HIV and HCV screening methods and acquiring large quantities of valid patient-level data from pragmatic trials. Dr. Haukoos and members of the study team have experience overseeing and successfully completing large prospective ED-based HIV screening and multi-center studies. The research team will hold regular conference calls and will visit each study site prior to beginning enrollment to ensure all procedures are being appropriately implemented. An additional potential difficulty relates to the within-nurse variability of screening approaches used during this trial. Although variability

may attenuate the effect of the interventions, this trial was specifically designed as a pragmatic effectiveness evaluation, thus allowing for flexibility in how screening is performed to maximize external validity and generalizability.

Aim 2 – "Linkage-to-Care Trial"

The ability to contact HCV positive patients after discharge from the ED will be important for provision of RNA results and for ongoing engagement with the linkage navigator. If patients are diagnosed, enrolled to Aim 2 and assigned to Linkage Navigation arm during non-business study hours, the linkage navigator will make contact the next business day, as is the case with our well-established HIV linkage program and consistent with practical clinical practice. Although the research assistant will attempt to obtain comprehensive contact information for patients, difficulty reaching patients will still be possible. In these instances the linkage navigator will attempt to contact patients through their emergency contact list or by sending a letter to their residence. We will use standardized methods developed by Hollander *et al.* to maximize the ability to follow-up high-risk patients.[119] Also, it may be challenging to evaluate linkage-to-care for individuals who receive primary care outside Denver Health, though many of them may still elect to receive specialty care at Denver Health. Obtaining outcome measures for patients assigned to linkage navigation will be principally obtained via the navigator and optimized by the patient's relationship with the navigator; however, for patients assigned to clinician referral only, determining successful linkage in other systems may be difficult and will involve accessing records through Epic and CORHIO when possible. Thus, we will obtain consent to obtain details related to interval care, and obtain permission for release of health information in the event it is required. Finally, we anticipate using random block sampling given that the trial in Aim 1 will enroll 24 hours per day. We will structure the research assistant's schedule to maximize the potential for enrollment into Aim 2 while using random time blocks to obtain a representative ED sample, and if enrollment in the former trial concludes before enrollment in the latter, we will extend HCV screening in the ED to achieve enrollment targets for this trial. To further optimize enrollment opportunity to Aim 2, patients whose confirmatory RNA result returns positive will be offered the opportunity to return to the Denver Health Emergency Department to participate in Aim 2.

Aim 3 – "Cost Effectiveness"

The primary difficulties for this aim reflect data collection as part of the screening and linkage-to-care trials, and any inherent limitations of the economic modeling.

Aim 4 – "Disparities and Social Determinants of Health"

We plan to optimize enrollment in Aim 2 of this trial to maximize the number of women and attempt to balance the ratio between men and women. If imbalance remains after enrollment, we may need to selectively enroll women to optimize the number of participants and to ensure robust analyses are performed. Because this trial will not start enrollment until 2019, there will be no complications merging data collected as a result of the administrative supplement with previously collected data. All data will be collected in a uniform manner when enrollment begins. The relationships between gender and linkage to HCV care, treatment adherence, and cure are largely unknown, which prevents us from better understanding their potential causal relationship. As such, we anticipate this aim will be exploratory and hypothesis generating, although we also believe important understanding of the effect of gender on the HCV care continuum will result from these additional aims.

Convenience sampling of participants will be used and participants will be enrolled who are ultimately determined to be HCV RNA negative. These latter participants will not be included in the final analyses as they, by definition, will not benefit from linkage to care or treatment for HCV. Each site will implement processes to optimize notification of true HCV positive patients by building a notification system via Epic to identify patients who may have been missed during regular enrollment hours.

J. Data Monitoring Plan

This study is not a Phase I, II, or III clinical trial, and therefore will not specifically use a Data and Safety Monitoring Board (**DSMB**).

The study investigators will make every effort to keep each patient's data safe and confidential. The Principal Investigators and Project Manager will oversee all data collection and will regularly monitor data collection and integrity. All electronic data queried from the electronic health record systems at each institution will be kept in password protected Excel (Microsoft Corporation, Redmond, WA) files using protected networks. The files will be stored on password-protected computers in the Project Coordinators' offices for each site prior to transfer to the DCC. Additional data will be collected from patients who test positive for HCV infection, as described above, and who consent for participation in the follow-up aspect of this project. These data will be recorded on closed-response data collection instruments, and stored in a locked file cabinet in the Project Coordinators' offices. These data will be manually entered into the main electronic REDCap database. During all aspects of this project, all data will be kept in secure locations either in the offices of the principal investigators, the office of the project manager, or the offices of the project coordinators. The principal investigators will take full responsibility for the protection of all documents. The electronic database will be stored on a password-protected computer in a locked room and accessible only by study investigators for either data entry or data analysis. In order to further minimize the risk of breach of confidentiality, study data will not be stored on a laptop computer or other portable storage unit (e.g., "jump or flash drive"). All collected data will follow institutional security regulations for additional protection. Once complete, the concatenated database will be completely de-identified and all study-related documents with protected health information will be destroyed.

Because of the "pragmatic" nature of the clinical trial for Aim 1, and because all study procedures are considered minimal risk (see above), we do not anticipate requiring a data safety and monitoring board (DSMB) for this study. According to the National Institutes of Health Standard Operating Procedures for human subjects' protection, "[i]f you are conducting a multisite clinical trial involving interventions that pose more than minimal risk to participants, you must have a…DSMB to monitor the trial".

K. Summary of Knowledge to be Gained

This project will help inform public health practices of how best to identify patients with undiagnosed HCV infection in EDs and other high-volume clinical settings in the United States, and provide important information related to improving the timeliness of diagnosis of HCV infection and linkage-to-care. This project's multi-centered design will contribute to the generalizability and external validity of the findings, and the knowledge gained by this study will offset the potential risks to participants.

8. STATISTICAL ANALYSES PLANS FOR THE CLINICAL TRIALS

All statistical analyses will be performed using SAS Enterprise Guide 7.1 (SAS Institute, Inc., Cary NC) or Stata Version 16 (StataCorp LLC, College Station, TX), or their most current versions, and conducted by the study's principal investigators and biostatistician after locking the datasets.

Continuous data will be reported as means with standard deviation (SDs) or medians with interquartile ranges (IQRs), depending on their distributions, and categorical data as proportions or percentages with 95% confidence intervals (CIs). Bivariate statistical tests (e.g., Student's t, Wilcoxon rank sum test, chi-square, Fisher's exact test, etc.) will be used to compare variables between study groups. All statistical tests will be performed using two-sided testing and with $P<0.05$ defining statistical significant. All precision estimates will include adjustment for institution-level clustering.

A. Aim 1 – "Emergency Department Screening Trial"

The primary analysis will be performed using the intention-to-treat principle and no interim analyses are planned given the pragmatic trial approach and minimal risk to subjects. The primary analysis will be performed using the intention-to-treat principle. Patient-level data will be reported for all patient-level variables (e.g., age, gender), although the primary unit of analysis will be patient visits. Given the randomized design, the primary comparison will include an unadjusted risk ratio (RR) for newly-identified HCV cases (primary outcome) with 95% CIs, specifically comparing nontargeted HCV screening to targeted HCV screening (primary hypothesis), while using a random effect hierarchical model to account for institution-level clustering, if needed. Statistical significance for the primary analysis will be defined as $p<0.05$ based on two-tailed statistical testing, which includes a lower 95% confidence limit of the RR >1.0. Absolute differences with 95% CIs will also be calculated while also accounting for institution-level clustering. Secondary comparisons will include all other outcomes by study arm, and further stratified by age, gender, race, ethnicity, income, education level, and institution. Although no formal interim analyses are planned for the Screening Trial, although the study team may perform preliminary analyses for purposes of presentation at scientific meetings; these instances, if they occur, will be explicitly qualified as such and described as preliminary.

B. Aim 2 – "Linkage-to-Care Trial"

The primary analysis will be performed using the intention-to-treat principle. Bivariate statistical tests will be used to compare variables, including results from the ACASI surveys between study groups. Given the randomized design, the primary comparison will include the absolute percentage difference and RR with 95% CIs for initiation of HCV treatment (primary outcome), and tested using chi-square or Fisher's exact test. Kaplan-Meier and Cox proportional hazard regression will also be used to estimate associations between the interventions and outcomes when modeled using time-to-event. One interim effectiveness analyses is planned and will be performed by the study's biostatistician while maintaining blinding. Using the approach by O'Brien-Fleming, the interim analysis will occur at the study's halfway point, after 140 total patients (approximately 35 per arm) have been enrolled and outcomes data collected, and with an effectiveness threshold of $p<0.0054$ for the primary outcome. A non-binding futility threshold of $p>0.5$ will be used for the primary outcome. If the trial is not stopped after the interim analysis, it will proceed to enroll the full sample (280) with a significance effectiveness threshold of $p<0.0492$. Statistical significance will be defined as $p<0.05$ at the final analysis, which includes a lower 95% confidence limit of the absolute difference of >0. The unit of analysis will be the patient. Secondary comparisons will include all other outcomes by study arm and with subgroup analyses for the stratum of individuals <40 years of age or PWID, age, gender, race, ethnicity, income, and education level.

9. REFERENCES

1. Linas BP, Barter DM, Leff JA, et al. The cost-effectiveness of improved hepatitis C virus therapies in HIV/hepatitis C virus coinfected patients. *Aids.* Jan 28 2014;28(3):365-376.
2. Linas BP, Barter DM, Leff JA, et al. The hepatitis C cascade of care: identifying priorities to improve clinical outcomes. *PLoS One.* 2014;9(5):e97317.
3. Denniston MM, Jiles RB, Drobeniuc J, et al. Chronic hepatitis C virus infection in the United States, National Health and Nutrition Examination Survey 2003 to 2010. *Ann Intern Med.* Mar 4 2014;160(5):293-300.
4. Yehia BR, Schranz AJ, Umscheid CA, Lo Re V, 3rd. The treatment cascade for chronic hepatitis C virus infection in the United States: a systematic review and meta-analysis. *PLoS One.* 2014;9(7):e101554.
5. Chak E, Talal AH, Sherman KE, Schiff ER, Saab S. Hepatitis C virus infection in USA: an estimate of true prevalence. *Liver Int.* Sep 2011;31(8):1090-1101.
6. Fattovich G, Giustina G, Degos F, et al. Morbidity and mortality in compensated cirrhosis type C: a retrospective follow-up study of 384 patients. *Gastroenterology.* Feb 1997;112(2):463-472.
7. Ly KN, Xing J, Klevens RM, Jiles RB, Ward JW, Holmberg SD. The increasing burden of mortality from viral hepatitis in the United States between 1999 and 2007. *Ann Intern Med.* Feb 21 2012;156(4):271-278.
8. Allison RD, Tong X, Moorman AC, et al. Increased incidence of cancer and cancer-related mortality among persons with chronic hepatitis C infection, 2006-2010. *J Hepatol.* May 1 2015.
9. Kanwal F, Hoang T, Kramer JR, et al. Increasing prevalence of HCC and cirrhosis in patients with chronic hepatitis C virus infection. *Gastroenterology.* Apr 2011;140(4):1182-1188 e1181.
10. Chen SL, Morgan TR. The natural history of hepatitis C virus (HCV) infection. *Int J Med Sci.* 2006;3(2):47-52.
11. Armstrong GL, Wasley A, Simard EP, McQuillan GM, Kuhnert WL, Alter MJ. The prevalence of hepatitis C virus infection in the United States, 1999 through 2002. *Ann Intern Med.* May 16 2006;144(10):705-714.
12. Mahajan R, Xing J, Liu SJ, et al. Mortality among persons in care with hepatitis C virus infection: the Chronic Hepatitis Cohort Study (CHeCS), 2006-2010. *Clinical infectious diseases : an official publication of the Infectious Diseases Society of America.* Apr 2014;58(8):1055-1061.
13. Wong JB, McQuillan GM, McHutchison JG, Poynard T. Estimating future hepatitis C morbidity, mortality, and costs in the United States. *Am J Public Health.* Oct 2000;90(10):1562-1569.
14. Kim AY, Onofrey S, Church DR. An epidemiologic update on hepatitis C infection in persons living with or at risk of HIV infection. *J Infect Dis.* Mar 2013;207 Suppl 1:S1-6.
15. Viner K, Kuncio D, Newbern EC, Johnson CC. The continuum of hepatitis C testing and care. *Hepatology.* Mar 2015;61(3):783-789.
16. Rowan SE, Blum J, Spielmann KW, et al. Optimization of the HCV Testing and Care Continuum in an Urban Federally Qualified Health Center. Paper presented at: ID Week2014; Philadelphia.
17. Wyles DL, Ruane PJ, Sulkowski MS, et al. Daclatasvir plus Sofosbuvir for HCV in Patients Coinfected with HIV-1. *N Engl J Med.* Aug 20 2015;373(8):714-725.
18. Feld JJ, Jacobson IM, Hezode C, et al. Sofosbuvir and Velpatasvir for HCV Genotype 1, 2, 4, 5, and 6 Infection. *N Engl J Med.* Dec 31 2015;373(27):2599-2607.

19. Feld JJ, Kowdley KV, Coakley E, et al. Treatment of HCV with ABT-450/r-ombitasvir and dasabuvir with ribavirin. *N Engl J Med.* Apr 24 2014;370(17):1594-1603.
20. Afdhal N, Reddy KR, Nelson DR, et al. Ledipasvir and sofosbuvir for previously treated HCV genotype 1 infection. *N Engl J Med.* Apr 17 2014;370(16):1483-1493.
21. Afdhal N, Zeuzem S, Kwo P, et al. Ledipasvir and sofosbuvir for untreated HCV genotype 1 infection. *N Engl J Med.* May 15 2014;370(20):1889-1898.
22. Foster GR, Afdhal N, Roberts SK, et al. Sofosbuvir and Velpatasvir for HCV Genotype 2 and 3 Infection. *N Engl J Med.* Dec 31 2015;373(27):2608-2617.
23. Rowan SE, Rogers M, Bayer J, et al. Treatment of Hepatitis C Virus in HIV-Coinfected Individuals in Real-world Clinical Settings: Results From 2 Large HIV Care Clinics. *Clin Infect Dis.* Oct 1 2016;63(7):994-995.
24. Department of Health & Human Services U. Action plan for the prevention, care, & treatment of viral hepatitis. 2016. https://www.aids.gov/pdf/viral-hepatitis-action-plan.pdf. Accessed November 1, 2016.
25. Recommendations for prevention and control of hepatitis C virus (HCV) infection and HCV-related chronic disease. Centers for Disease Control and Prevention. *MMWR Recomm Rep.* Oct 16 1998;47(RR-19):1-39.
26. Smith BD, Morgan RL, Beckett GA, et al. Recommendations for the identification of chronic hepatitis C virus infection among persons born during 1945-1965. *MMWR Recomm Rep.* Aug 17 2012;61(RR-4):1-32.
27. Infections IoMotNA-CotPaCoVH. *Hepatitis and Liver Cancer: A National Strategy for Prevention and Control of Hepatitis B and C.* Washington D.C.2010.
28. Moyer VA, Force USPST. Screening for hepatitis C virus infection in adults: U.S. Preventive Services Task Force recommendation statement. *Ann Intern Med.* Sep 3 2013;159(5):349-357.
29. Hepatitis C guidance: AASLD-IDSA recommendations for testing, managing, and treating adults infected with hepatitis C virus. *Hepatology.* Sep 2015;62(3):932-954.
30. Coyle C, Viner K, Hughes E, et al. Identification and Linkage to Care of HCV-Infected Persons in Five Health Centers - Philadelphia, Pennsylvania, 2012-2014. *MMWR Morb Mortal Wkly Rep.* May 8 2015;64(17):459-463.
31. Foundation THJKF. The Kaiser Commission on Medicaid and the Uninsured. 2015; http://files.kff.org/attachment/fact-sheet-key-facts-about-the-uninsured-population.
32. Bodenheimer T, Pham HH. Primary care: current problems and proposed solutions. *Health affairs.* May 2010;29(5):799-805.
33. Pitts SR, Carrier ER, Rich EC, Kellermann AL. Where Americans get acute care: increasingly, it's not at their doctor's office. *Health affairs.* Sep 2010;29(9):1620-1629.
34. Galbraith JW. Hepatitis C Virus Screening: An Important Public Health Opportunity for United States Emergency Departments. *Ann Emerg Med.* Jan 2016;67(1):129-130.
35. Abraham MK, Perkins J, Vilke GM, Coyne CJ. Influenza in the Emergency Department: Vaccination, Diagnosis, and Treatment: Clinical Practice Paper Approved by American Academy of Emergency Medicine Clinical Guidelines Committee. *The Journal of emergency medicine.* Jan 4 2016.
36. Lacroix S, Vrignaud B, Avril E, et al. Impact of rapid influenza diagnostic test on physician estimation of viral infection probability in paediatric emergency department during epidemic period. *Journal of clinical virology : the official publication of the Pan American Society for Clinical Virology.* Nov 2015;72:141-145.
37. Bernstein SL, Shayne P. Ebola, physicians in training, and the duty to treat. *Academic emergency medicine : official journal of the Society for Academic Emergency Medicine.* Jan 2015;22(1):88-90.

38. Meyers L, Frawley T, Goss S, Kang C. Ebola virus outbreak 2014: clinical review for emergency physicians. *Annals of emergency medicine.* Jan 2015;65(1):101-108.
39. Rothman RE, Lyons MS, Haukoos JS. Uncovering HIV infection in the emergency department: a broader perspective. *Academic emergency medicine : official journal of the Society for Academic Emergency Medicine.* Jul 2007;14(7):653-657.
40. Haukoos JS. Rethinking How We Perform HIV Testing in the Emergency Department. *Ann Emerg Med.* Jul 2011;58 Suppl 1:S160-163.
41. Haukoos JS, Hopkins E. Understanding HIV screening in the emergency department: is perception reality? *Academic emergency medicine : official journal of the Society for Academic Emergency Medicine.* Mar 2013;20(3):309-312.
42. Haukoos JS, Hopkins E, Bucossi MM. Routine opt-out HIV screening: more evidence in support of alternative approaches? *Sex Transm Dis.* Jun 2014;41(6):403-406.
43. Haukoos JS, Lyons MS. Idealized models or incremental program evaluation: translating emergency department HIV testing into practice. *Acad Emerg Med.* Nov 2009;16(11):1044-1048.
44. Haukoos JS, Lyons MS, White DA, Hsieh YH, Rothman RE. Acute HIV infection and implications of fourth-generation HIV screening in emergency departments. *Annals of emergency medicine.* Nov 2014;64(5):547-551.
45. Haukoos JS, Mallory M, Witt M, Coil CJ, Lewis RJ. Do patient perceptions influence complaince with outpatient HIV testing referrals from the emergency department? An application of the health belief model. *Society for Academic Emergency Medicine-Annual Meeting*. Orlando, Florida2004.
46. Haukoos JS, Mehta SD, Harvey L, Calderon Y, Rothman RE. Research priorities for human immunodeficiency virus and sexually transmitted infections surveillance, screening, and intervention in emergency departments: consensus-based recommendations. *Acad Emerg Med.* Nov 2009;16(11):1096-1102.
47. Kelen GD, Green GB, Purcell RH, et al. Hepatitis B and hepatitis C in emergency department patients. *N Engl J Med.* May 21 1992;326(21):1399-1404.
48. Kelen GD, DiGiovanna T, Bisson L, Kalainov D, Sivertson KT, Quinn TC. Human immunodeficiency virus infection in emergency department patients. Epidemiology, clinical presentations, and risk to health care workers: the Johns Hopkins experience. *Jama.* Jul 28 1989;262(4):516-522.
49. Kelen GD, Fritz S, Qaqish B, et al. Unrecognized human immunodeficiency virus infection in emergency department patients. *N Engl J Med.* Jun 23 1988;318(25):1645-1650.
50. Centers for Disease Control and Prevention. National Hospital Ambulatory Medical Care Survey: 2017 Emergency Department Summary Tables. http://www.cdc.gov/nchs/data/nhamcs/web_tables/2017_ed_web_tables-508.pdf. Accessed November 20, 2020.
51. Jenkins TC, Gardner EM, Thrun MW, Cohn DL, Burman WJ. Risk-based human immunodeficiency virus (HIV) testing fails to detect the majority of HIV-infected persons in medical care Settings. *Sex Transm Dis.* May 2006;33(5):329-333.
52. Centers for Disease Control and Prevention. Revised guidelines for HIV counseling, testing, and referral. *MMWR Recomm Rep.* Nov 9 2001;50(RR-19):1-57; quiz CE51-19a51-CE56-19a51.
53. Hall HI, An Q, Tang T, et al. Prevalence of Diagnosed and Undiagnosed HIV Infection--United States, 2008-2012. *MMWR Morb Mortal Wkly Rep.* Jun 26 2015;64(24):657-662.
54. Haukoos JS. The impact of nontargeted HIV screening in emergency departments and the ongoing need for targeted strategies. *Archives of internal medicine.* Jan 9 2012;172(1):20-22.

55. Branson BM, Handsfield HH, Lampe MA, et al. Revised recommendations for HIV testing of adults, adolescents, and pregnant women in health-care settings. *MMWR Recomm Rep.* Sep 22 2006;55(RR-14):1-17; quiz CE11-14.

56. Haukoos JS, Hopkins E, Byyny RL, et al. Design and implementation of a controlled clinical trial to evaluate the effectiveness and efficiency of routine opt-out rapid human immunodeficiency virus screening in the emergency department. *Academic emergency medicine : official journal of the Society for Academic Emergency Medicine.* Aug 2009;16(8):800-808.

57. Haukoos JS, Hopkins E, Bender B, Sasson C, Al-Tayyib AA, Thrun MW. Comparison of enhanced targeted rapid HIV screening using the Denver HIV risk score to nontargeted rapid HIV screening in the emergency department. *Annals of emergency medicine.* Mar 2013;61(3):353-361.

58. Rothman RE, Hsieh YH, Harvey L, et al. 2009 US Emergency Department HIV Testing Practices. *Ann Emerg Med.* Jul 2011;58 Suppl 1:S3-S9 e4.

59. White DA, Anderson ES, Pfeil SK, Trivedi TK, Alter HJ. Results of a Rapid Hepatitis C Virus Screening and Diagnostic Testing Program in an Urban Emergency Department. *Ann Emerg Med.* Jan 2016;67(1):119-128.

60. Galbraith JW, Franco RA, Donnelly JP, et al. Unrecognized chronic hepatitis C virus infection among baby boomers in the emergency department. *Hepatology.* Mar 2015;61(3):776-782.

61. Donnelly JP, Franco RA, Wang HE, Galbraith JW. Emergency Department Screening for Hepatitis C Virus: Geographic Reach and Spatial Clustering in Central Alabama. *Clin Infect Dis.* Nov 26 2015.

62. Assoumou SA, Huang W, Horsburgh CR, Jr., Drainoni ML, Linas BP. Relationship between hepatitis C clinical testing site and linkage to care. *Open Forum Infect Dis.* Mar 2014;1(1):ofu009.

63. Craw JA, Gardner LI, Marks G, et al. Brief strengths-based case management promotes entry into HIV medical care: results of the antiretroviral treatment access study-II. *J Acquir Immune Defic Syndr.* Apr 15 2008;47(5):597-606.

64. Gardner LI, Metsch LR, Anderson-Mahoney P, et al. Efficacy of a brief case management intervention to link recently diagnosed HIV-infected persons to care. *Aids.* Mar 4 2005;19(4):423-431.

65. Laffoon BT, Hall HI, Surendera Babu A, et al. HIV Infection and Linkage to HIV-Related Medical Care in Large Urban Areas in the United States, 2009. *J Acquir Immune Defic Syndr.* Aug 1 2015;69(4):487-492.

66. Trooskin SB, Reynolds H, Kostman JR. Access to Costly New Hepatitis C Drugs: Medicine, Money, and Advocacy. *Clin Infect Dis.* Dec 15 2015;61(12):1825-1830.

67. Turner BJ, Taylor BS, Hanson JT, et al. Implementing hospital-based baby boomer hepatitis C virus screening and linkage to care: Strategies, results, and costs. *J Hosp Med.* Aug 2015;10(8):510-516.

68. Rowan SE SK, Blum J, Vaughn S, Gardner EM, Burman WJ Optimization of the HCV Testing and Care Continuum in an Urban Federally Qualified Health Center. Paper presented at: ID Week2014; Philadelphia.

69. Centers for Disease Control and Prevention. *Surveillance for Viral Hepatitis - United States, 2015*2016.

70. Colorado Department of Public Health and Environment. *2016 Surveillance Report.* https://www.colorado.gov/pacific/cdphe/hepatitis-data September 2017 2017.

71. Curry MP, Forns X, Chung RT, et al. Sofosbuvir and ribavirin prevent recurrence of HCV infection after liver transplantation: an open-label study. *Gastroenterology.* Jan 2015;148(1):100-107 e101.

72. Kowdley KV, Gordon SC, Reddy KR, et al. Ledipasvir and sofosbuvir for 8 or 12 weeks for chronic HCV without cirrhosis. *N Engl J Med.* May 15 2014;370(20):1879-1888.

73. Naggie S, Cooper C, Saag M, et al. Ledipasvir and Sofosbuvir for HCV in Patients Coinfected with HIV-1. *N Engl J Med.* Aug 20 2015;373(8):705-713.

74. Read P, Lothian R, Chronister K, et al. Delivering direct acting antiviral therapy for hepatitis C to highly marginalised and current drug injecting populations in a targeted primary health care setting. *Int J Drug Policy.* Sep 2017;47:209-215.

75. Mah A, Hull MW, DeBeck K, et al. Knowledge of hepatitis C and treatment willingness amongst people who inject drugs in an era of direct acting antivirals. *Int J Drug Policy.* Sep 2017;47:137-143.

76. Patrick SW, Bauer AM, Warren MD, Jones TF, Wester C. Hepatitis C Virus Infection Among Women Giving Birth - Tennessee and United States, 2009-2014. *MMWR Morb Mortal Wkly Rep.* May 12 2017;66(18):470-473.

77. Watts T, Stockman L, Martin J, Guilfoyle S, Vergeront JM. Increased Risk for Mother-to-Infant Transmission of Hepatitis C Virus Among Medicaid Recipients - Wisconsin, 2011-2015. *MMWR Morb Mortal Wkly Rep.* Oct 27 2017;66(42):1136-1139.

78. Koneru A, Nelson N, Hariri S, et al. Increased Hepatitis C Virus (HCV) Detection in Women of Childbearing Age and Potential Risk for Vertical Transmission - United States and Kentucky, 2011-2014. *MMWR Morb Mortal Wkly Rep.* Jul 22 2016;65(28):705-710.

79. Krans EE, Rothenberger SD, Morrison PK, et al. Hepatitis C Virus Knowledge Among Pregnant Women with Opioid Use Disorder. *Matern Child Health J.* Aug 2018;22(8):1208-1216.

80. Concato J, Shah N, Horwitz RI. Randomized, controlled trials, observational studies, and the hierarchy of research designs. *N Engl J Med.* Jun 22 2000;342(25):1887-1892.

81. Grebely J, Matthews GV, Hellard M, et al. Adherence to treatment for recently acquired hepatitis C virus (HCV) infection among injecting drug users. *J Hepatol.* Jul 2011;55(1):76-85.

82. Lin Y, Zhu M, Su Z. The pursuit of balance: An overview of covariate-adaptive randomization techniques in clinical trials. *Contemp Clin Trials.* Nov 2015;45(Pt A):21-25.

83. Coffin PO, Scott JD, Golden MR, Sullivan SD. Cost-effectiveness and population outcomes of general population screening for hepatitis C. *Clin Infect Dis.* May 2012;54(9):1259-1271.

84. McGarry LJ, Pawar VS, Panchmatia HR, et al. Economic model of a birth cohort screening program for hepatitis C virus. *Hepatology.* May 2012;55(5):1344-1355.

85. Rein DB, Smith BD, Wittenborn JS, et al. The cost-effectiveness of birth-cohort screening for hepatitis C antibody in U.S. primary care settings. *Ann Intern Med.* Feb 21 2012;156(4):263-270.

86. Linas BP, Barter DM, Morgan JR, et al. The cost-effectiveness of sofosbuvir-based regimens for treatment of hepatitis C virus genotype 2 or 3 infection. *Ann Intern Med.* May 5 2015;162(9):619-629.

87. Schackman BR, Leff JA, Barter DM, et al. Cost-effectiveness of rapid hepatitis C virus (HCV) testing and simultaneous rapid HCV and HIV testing in substance abuse treatment programs. *Addiction.* Jan 2015;110(1):129-143.

88. Bauman LJ, Braunstein S, Calderon Y, et al. Barriers and facilitators of linkage to HIV primary care in New York City. *J Acquir Immune Defic Syndr.* Nov 1 2013;64 Suppl 1:S20-26.

89. Haukoos JS, Hopkins E, Eliopoulos VT, et al. Development and implementation of a model to improve identification of patients infected with HIV using diagnostic rapid testing in the emergency department. *Acad Emerg Med.* Dec 2007;14(12):1149-1157.

90. Haukoos JS, Hopkins E, Conroy AA, et al. Routine opt-out rapid HIV screening and detection of HIV infection in emergency department patients. *Jama.* Jul 21 2010;304(3):284-292.

91. Haukoos JS, Hopkins E, Bender B, et al. Use of kiosks and patient understanding of opt-out and opt-in consent for routine rapid human immunodeficiency virus screening in the emergency department. *Academic emergency medicine : official journal of the Society for Academic Emergency Medicine.* Mar 2012;19(3):287-293.

92. Hsieh YH, Holtgrave DR, Peterson S, Gaydos CA, Rothman RE. Novel emergency department registration kiosk for HIV screening is cost-effective. *AIDS Care.* Oct 18 2015:1-4.

93. Lyons MS, Lindsell CJ, Ledyard HK, Frame PT, Trott AT. Emergency department HIV testing and counseling: an ongoing experience in a low-prevalence area. *Ann Emerg Med.* Jul 2005;46(1):22-28.

94. Lyons MS, Lindsell CJ, Ruffner AH, et al. Randomized Comparison of Universal and Targeted HIV Screening in the Emergency Department. *J Acquir Immune Defic Syndr.* Jul 10 2013.

95. White DA, Tran T, Dideum PJ, et al. Physician-Initiated Rapid HIV Testing in an Urban Emergency Department: Comparison of Testing Using a Point-of-Care Versus a Laboratory Model. *Ann Emerg Med.* Jul 2011;58 Suppl 1:S53-59.

96. White DA, Scribner AN, Vahidnia F, et al. HIV Screening in an Urban Emergency Department: Comparison of Screening Using an Opt-In Versus an Opt-Out Approach. *Ann Emerg Med.* Jul 2011;58 Suppl 1:S89-95.

97. White DA, Scribner AN, Schulden JD, Branson BM, Heffelfinger JD. Results of a Rapid HIV Screening and Diagnostic Testing Program in an Urban Emergency Department. *Ann Emerg Med.* Nov 4 2009;54(1):56-64.

98. White DA, Scribner AN, Huang JV. A comparison of patient acceptance of fingerstick whole blood and oral fluid rapid HIV screening in an emergency department. *J Acquir Immune Defic Syndr.* Sep 1 2009;52(1):75-78.

99. White DA, Sadoun T, Tran T, Alter HJ. Increased Acceptance Rates of HIV Screening Using Opt-Out Consent Methods in an Urban Emergency Department. *J Acquir Immune Defic Syndr.* Aug 26 2011.

100. White DA, Cheung PT, Scribner AN, Frazee BW. A comparison of HIV testing in the emergency department and urgent care. *J Emerg Med.* Jun 20 2009;20:20.

101. White DA, Anderson ES, Pfeil SK, Trivedi TK. Hepatitis C Virus Antibody Testing: Result Availability at Time of Discharge for Emergency Department Patients. *J Acquir Immune Defic Syndr.* Nov 2 2015.

102. Haukoos J, Rowan SE, Hopkins EH, Bucossi M. HCV screening in an integrated healthcare system: missed opportunities2016.

103. Investigators. THTT. The HIV Testing using Enhanced Screening Techniques in Emergency Departments (TESTED) Trial. 2014; http://www.denveremresearch.org/hiv-projects/. Accessed June 23, 2014.

104. Haukoos JS, Lyons MS, Lindsell CJ, et al. Derivation and validation of the Denver Human Immunodeficiency Virus (HIV) risk score for targeted HIV screening. *American journal of epidemiology.* Apr 15 2012;175(8):838-846.

105. Haukoos JS, Hopkins E, Bucossi MM, et al. Brief report: Validation of a quantitative HIV risk prediction tool using a national HIV testing cohort. *J Acquir Immune Defic Syndr.* Apr 15 2015;68(5):599-603.

106. Zwarenstein M, Treweek S, Gagnier JJ, et al. Improving the reporting of pragmatic trials: an extension of the CONSORT statement. *BMJ.* 2008;337:a2390.

107. Grembowski D. *The Practice of Health Program Evaluation*. Thousand Oaks: Sage Publications, Inc.; 2001.

108. Thorpe KE, Zwarenstein M, Oxman AD, et al. A pragmatic-explanatory continuum indicator summary (PRECIS): a tool to help trial designers. *Journal of clinical epidemiology.* May 2009;62(5):464-475.

109. Campbell DT, Stanley JC, Gage NL. *Experimental and quasi-experimental designs for research*. Dallas: Houghton Mifflin; 1963.

110. Gilbert EH, Lowenstein SR, Koziol-McLain J, Barta DC, Steiner J. Chart reviews in emergency medicine research: Where are the methods? *Annals of emergency medicine.* Mar 1996;27(3):305-308.

111. Rothman KJ, Greenland S, Lash TL. *Modern Epidemiology*. 3rd ed. Philadelphia: Lippincott Williams & Wilkins; 2008.

112. Snijders T, Bosker R. *Multilevel Analysis: An Introduction to Basic and Advanced Multilevel Modeling*. Thousand Oaks: SAGE Publications, Inc.; 2002.

113. Harris PA, Taylor R, Thielke R, Payne J, Gonzalez N, Conde JG. Research electronic data capture (REDCap)--a metadata-driven methodology and workflow process for providing translational research informatics support. *J Biomed Inform.* Apr 2009;42(2):377-381.

114. Gabow P, Eisert S, Wright R. Denver Health: A Model for the Integration of a Public Hospital and Community Health Centers. *Ann Intern Med.* January 21, 2003 2003;138(2):143-149.

115. Gardner LI, Marks G, Metsch LR, et al. Psychological and behavioral correlates of entering care for HIV infection: the Antiretroviral Treatment Access Study (ARTAS). *AIDS Patient Care STDS.* Jun 2007;21(6):418-425.

116. Craw J, Gardner L, Rossman A, et al. Structural factors and best practices in implementing a linkage to HIV care program using the ARTAS model. *BMC Health Serv Res.* 2010;10:246.

117. Islam MM, Topp L, Conigrave KM, et al. The reliability of sensitive information provided by injecting drug users in a clinical setting: clinician-administered versus audio computer-assisted self-interviewing (ACASI). *AIDS Care.* 2012;24(12):1496-1503.

118. Williams ML, Freeman RC, Bowen AM, et al. A comparison of the reliability of self-reported drug use and sexual behaviors using computer-assisted versus face-to-face interviewing. *AIDS Educ Prev.* Jun 2000;12(3):199-213.

119. Weber JE, Shofer FS, Larkin GL, Kalaria AS, Hollander JE. Validation of a brief observation period for patients with cocaine-associated chest pain. *N Engl J Med.* Feb 6 2003;348(6):510-517.

120. Linas BP, Wong AY, Schackman BR, Kim AY, Freedberg KA. Cost-effective screening for acute hepatitis C virus infection in HIV-infected men who have sex with men. *Clin Infect Dis.* Jul 2012;55(2):279-290.

121. National Bureau of Labor Statistics. Overview of Wage Data by Area and Occupation. 2016; http://www.bls.gov/bls/blswage.htm. Accessed November 1, 2016.

122. Haukoos JS, Campbell JD, Conroy AA, et al. Programmatic cost evaluation of nontargeted opt-out rapid HIV screening in the emergency department. *PLoS One.* 2013;8(12):e81565.

123. Gold MR, Siegel JE, Russell LB, Weinstein MC. *Cost-Effectiveness in Health and Medicine*. New York: Oxford University Press, Inc.; 1996.

124. Drummond M, McGuire A. *Economic Evaluation in Health Care: Merging Theory with Practice*. New York: Oxford University Press; 2001.

125. Drummond MF, Sculpher MJ, Torrance GW, O'Brien BJ, Stoddart GL. *Methods for the Economic Evaluation of Health Care Programmes*. Third ed. New York: Oxford University Press; 2005.

126. Neumann PJ, Cohen JT, Weinstein MC. Updating cost-effectiveness--the curious resilience of the $50,000-per-QALY threshold. *N Engl J Med.* Aug 28 2014;371(9):796-797.

127. Jaton E, Stang J, Biros M, et al. The Use of Electronic Consent for COVID-19 Clinical Trials: Lessons for Emergency Care Research During a Pandemic and Beyond. *Acad Emerg Med.* Nov 2020;27(11):1183-1186.
